# Supplementary material for: Endotoxemia and circulating bacteriome in severe COVID-19 patients
Source: Intensive Care Med Exp. 2020 Dec 7;8:72. doi: 10.1186/s40635-020-00362-8 (PMC7719737; doi:10.1186/s40635-020-00362-8)
Supplement: Supplementary file 1 — Additional file 1. EAA laboratory specification manual. [file 40635_2020_362_MOESM1_ESM.pdf]

EAA™

CE

ENDOTOXIN ACTIVITY ASSAY

For In Vitro Diagnostic Use Only
Pour diagnostic in vitro uniquement
Nur Für Die In-vitro-Diagnostik
Pour diagnostic in vitro uniquement
Только для диагностики In Vitro
Yalnızca In Vitro Teşhis Kullanımı İçindir

EN

FR

DE

IT

RU

TR

SPECTRAL Medical INC

Endotoxin Activity Assay (EAA™)

EN

For In Vitro Diagnostic Use Only

1. Intended Use

The EAA™ is a rapid in vitro diagnostic test that utilizes a specific monoclonal antibody to measure the endotoxin activity in EDTA whole blood specimens.

The information, when used in conjunction with microbial cultures and other relevant diagnostic tests (i.e., ultrasound, bronchoscopy or CAT scan) and clinical information (clinical signs, and/or radiographic test results) can:

- Aid in the risk assessment of patients for progression to severe sepsis.
- Direct the use of specific anti-endotoxin therapy

2. Endotoxin Activity Assay – Introduction

The EAA test assesses the level of endotoxin activity in the blood specimen of a specific patient in a semi-quantitative way, distinguishing 3 groups – low, intermediate and high endotoxin activity.

Endotoxin or lipopolysaccharide (LPS) is a major cell wall constituent of Gram-negative bacteria and the primary Gram-negative bacterial product responsible for severe sepsis[1-2]. Elevated endotoxin levels cause changes in the expression of more than 300 genes and activated macrophages, neutrophils, endothelial cells and the coagulation cascade producing the sepsis cascade[3].

This assay uses the biological response of the neutrophils in a patient's blood to an immunological complex of endotoxin and exogenous antibody as a measure of the endotoxin activity in the patient[4].

The assay reacts specifically with the Lipid A moiety of LPS of Gram-negative bacteria and does not cross-react with cell wall constituents of Gram-positive bacteria and other microorganisms.

3. Contents

3.1 EAA™ Kit

Name	Quantity
EAAST-20	1
EAA™ Trays	20
EAA™ Quality Control Trays	1
EAA™ Solution Vials	21
Instructions for Use	1

3.2 EAA™-Trays

Name	Quantity	Description
Tube 1	2	Stabilizers and luminol-zymosan
Tube 2	2	Murine monoclonal anti-endotoxin antibody, stabilizers and luminol-zymosan
Tube 3	2	Murine monoclonal anti-endotoxin antibody, stabilizers and luminol-zymosan
LPS Max Tube	1	Endotoxin (E. coli O55:B5) with stabilizers
Aliquot Tube	1	Empty tube

3.3 EAA™ Quality Control Trays

Name	Quantity	Description
Tube 1	2	Stabilizers and luminol-zymosan
Low Control Tube	1	Murine monoclonal antibody, stabilizers and luminol-zymosan
High Control Tube	1	Murine monoclonal anti-endotoxin antibody, stabilizers and luminol-zymosan
Tube 3	1	Murine monoclonal anti-endotoxin antibody, stabilizers and luminol-zymosan
QC LPS Max Tube	1	Endotoxin (E. coli "055:B5") with stabilizers
QC Aliquot Tube	1	Empty tube

EN

3.4 EAA™ Solution

Name	Quantity	Description
EAA™ Solution	8 mL/Vial	HBSS and Heparin Solution

3.5 Accessories

Not supplied with the kit.

- Combipipettor
- 500 µL micropipette
- Sterile combipipette tips capable of delivering 40uL and 1000uL
- 1000uL micropipette tips
- EDTA blood collection tubes
- Timer
- Luminometer (Berthold Smartline TL Tube or equivalent)
- Incubating shaker capable of incubating samples at 37°C and agitating at 100 rpm
- Vortexer

4. Precautions

- Use general laboratory precautions when handling and storing the unopened assay materials.
- The EAA™ contains lyophilized beaded reagents in borosilicate glass test tubes. Under certain conditions (damaged packaging, improper sealing of the pouches or improper storage) the beads may shrink in the presence of excess humidity. Do not use tubes that contain shrunken beads as test results may be inaccurate.
- For manual pipetting of samples and controls, use individual pipette tips to eliminate carryover of residual.
- Do not use after the expiry date specified on the label.
- Use recommended universal precautions[5] for handling used solutions and human specimens.

EN

- Handle or dispose of used EAA™ solution and all human blood products using universal precautions.
- Do not pipette solutions by mouth.
- Clean up spills immediately with a 0.5% sodium hypochlorite solution.

5. Principle

A basal activity measurement (Tube 1) in the absence of the specific anti-endotoxin antibody measures the non-specific oxidative burst of the patient's neutrophils. An additional control measurement including the specific anti-endotoxin antibody and an excess of exogenous endotoxin (Tube 3) measures the maximum oxidative burst of the patient's neutrophils. The test measurement (Tube 2) includes the specific antibody to measure the actual level of endotoxin activity. The EAA result is calculated by normalizing the chemiluminescence in the test sample (Tube 2) against the maximum chemiluminescence (Tube 3), correcting both measurements for the basal activity chemiluminescence (Tube 1).

5.1 Specimen Collection and Preparation

Only use EDTA anti-coagulated blood collection tubes to obtain whole blood samples for this procedure. Guidelines recommended by the NCCLS[6] should be followed when collecting, transporting and processing patient samples. Samples must be collected in blood collection tubes containing EDTA anti-coagulant by venipuncture or through an indwelling catheter. If sampling through an indwelling arterial or venous cannula or catheter, please flush the lines per your facility's policy prior to collecting blood in the EDTA anti-coagulated tube. Blood samples may be stored for up to 180 minutes at an ambient temperature range of 18°C to 25°C prior to analysis. Blood must be thoroughly mixed for at least 20-30 seconds by gentle inversion immediately prior to analysis.

A minimum of 1.5 mL of patient whole blood is suggested to run the patient assay (1.0 mL required). A minimum of 1.5 mL of whole blood is suggested to run the QC assay (1.0 mL required). Blood for QC test may be an independent whole blood sample.

5.2 Instruction for use with Berthold Smartline TL Tube Luminometer

NOTE: Prior to starting the EAA™ or EAA™ QC Test, ensure that Berthold SmartLine TL Tube Luminometer is “ON”, and the heating incubator shaker is “ON”, and warmed to the setting 37°C ± 1.0°C.

5.2.1 EAA™ Test Procedure

Good laboratory practice suggests that the EAA be performed in duplicate for each patient. The average of the duplicates is used to report the EAA™ result. Each laboratory should develop and apply acceptable CV’s for the acceptance of replicates. You can perform up to 3 patient samples in one run.

1. Set up the EAA™ tubes for each patient sample in the supplied tube racks and remove the caps.
2. Combipipette 1.0 mL of the EAA™ Solution into Tubes 1, 2 and 3 (in duplicate).
3. After mixing the patient blood sample by gentle inversion, pipette 0.5 mL aliquots directly into the Aliquot Tube and the LPS MAX Tube. Vortex the LPS MAX tube.
4. Place the rack(s) in the incubating shaker, close the lid and incubate for 10 minutes.
5. Following the 10-minute incubation, open the lid, vortex the Aliquot tube then combipipette 40 µL of blood into Tubes 1 and 2 (in duplicate) using a sterile combipipette tip.
6. Vortex the LPS Max Tube, then combipipette 40 µL of blood into Tubes 3 (in duplicate) using a sterile combipipette tip.
7. Vortex Tubes 1, 2 and 3 and place back into the rack(s) in the incubating shaker, close the lid and start the motion (set at 100 rpm) and the timer (set at 14 minutes).
8. Insert the EAA labeled chip card into the luminometer and press Start.
9. Following the 14-minute incubation, follow the instructions on the luminometer display for reading the EAA tubes in the proper order.
10. Vortex each tube gently, open the sample drawer and place the tube in the sample holder, close the sample drawer and wait for an RLU reading. Repeat for the next tube.
11. After all tubes are read, the EAA results are printed automatically on the attached printout.
12. Repeat for each patient sample if applicable.

5.2.1.1 Interpretation of Results

Range (EAA™ result)	Interpretation of Results
0.0 to 0.39	Low endotoxin activity level represents a low risk for progression to severe sepsis.
0.40 to 0.59	Intermediate endotoxin activity level represents an elevated risk for severe sepsis.
≥ 0.60	High endotoxin activity level represents a high risk for developing severe sepsis

5.2.2 EAA™ QC Test Procedure

It is recommended that the QC test be run once per EAA™ kit box. However, each laboratory may produce their own QC testing regimen according to local standards.

1. Place the QC tubes into a supplied rack and remove the caps.
2. Combipipette 1.0 mL of the EAA™ Solution into each of the tubes.
3. Pipette 0.5 mL whole blood aliquots directly into the QC Aliquot Tube and the QC LPS MAX Tube. Vortex the QC LPS MAX tube.
4. Place the rack in the incubating shaker, close the lid and incubate for 10 minutes.
5. Following the 10-minute incubation, open the lid, vortex the QC Aliquot tube then combipipette 40 µL of blood into both Tube 1’s and the Low Control Tube using a sterile combipipette tip.
6. Vortex the QC LPS MAX Tube, then combipipette 40 µL of blood

EN

EN

into the High Control Tube first and then both Tube 3's using a sterile combipipette tip.

7. Vortex all tubes and place back into the tube rack in the incubating shaker and start the motion (set at 100 rpm) and the timer (set at 14 minutes).
8. Insert the labeled EAA QC chip card into the luminometer and press Start.
9. Following the 14-minute incubation, follow the instructions on the luminometer display for reading the EAA QC tubes in the proper order.
10. Vortex each tube gently, open the sample drawer and place the tube in the sample holder, close the sample drawer and wait for an RLU reading. Repeat for the next tube.
11. After all tubes are read, the EAA™ QC results are printed automatically on the attached printout.

5.2.2.1 Interpretation of Results

EAA™ Solution QC is confirmed with the following results:

Interpretation of Results	EAA™ Result
EAA Low QC	0.00 – 0.20
EAA High QC	0.80 – 1.50

If the QC test results for low or high control do not provide the desired results, please repeat the test. If the repeated QC test results remain outside acceptable limits, subsequent EAA results may be invalid. Please notify your EAA™ representative as soon as possible.

5.3 Limitations

5.3.1 Analytical

- As with all immunoassays or immunochemical reactions, careful technique is required.

EN

- The user should be alert to the possible effects on results of potential interferences from medications or unknown endogenous substances. See Interfering Substances below.

5.3.2 Clinical

- The results of the EAA™ should be evaluated in context of all laboratory findings and the total clinical status of the patient. In cases where the laboratory results do not agree with the clinical picture or history, additional tests should be performed.
- The findings of positive endotoxin results in patients without severe sepsis have been reported previously in published studies.
- Results obtained on patients outside of the critical care setting should be interpreted with caution as they may not be of a significant clinical value.

5.4 Clinical Performance

The Multi-Center Endotoxin Detection in Critical Illness (MEDIC) trial was a multi-center, prospective observational study, performed in 10 Intensive Care Units (ICUs) of academic hospitals in North America and Europe. The presence of endotoxemia was evaluated on the first day of the patients' ICU stay to determine the odds of developing severe sepsis within 24 hours of ICU admission. The target population for the risk assessment study included all eligible patients enrolled in the MEDIC trial on first day of ICU admission who had evaluable samples, N=857. Patient demographics are shown in the following table.

Demographics and Baseline Features of Analyzed Patients

Characteristic	
Age: mean (SD) median [IQR]* years	60 (±17) 62 [49, 74]
Gender (% male)	58.9%
Race (% Non-Caucasian)	15.8%
APACHEII: mean (SD) median [IQR]*	15.2 (±9,5) 14 [8, 21]

EN

Characteristic	
Days in ICU: mean (SD) median [IQR]*	5.4 (±10,9) 2.0 [1, 5]
Days in Hospital: mean (SD) median [IQR]*	23.3 (±27,0) 14.0 [7, 29]
ICU Mortality	13.3%
Hospital Mortality	19.9%

The critical care patient population is the most appropriate clinical environment for EAA™ testing. The utility of the EAA™ test has only been established for the critical care population. The relationship between endotoxin levels and the risk for developing severe sepsis in critically ill patients is intended as a supplement to currently available clinical information to establish risk for severe sepsis on the first day of ICU stay.

5.5 Performance Characteristics

5.5.1 Interfering Substances

- Triglyceride levels up to 1000 mg/dL do not interfere with EAA measurements. At triglyceride concentrations greater than 1500 mg/dL, a decrease in EAA results has been demonstrated. Grossly lipemic samples should be avoided for use in the EAA™.
- Hemoglobin at 100 mg/dL does not interfere with EAA measurements, while levels of 500 mg/dL attenuated low EAA™ measurements by less than EAA result of 0.03 and attenuated elevated EAA readings by an average of 15%. Grossly hemolyzed samples should be avoided for use in the EAA™.
- Exogenous infusion of endotoxin-free albumin into patients at levels which increase plasma albumin by more than 10 g/L above the upper limit of normal may lower EAA results. Samples from patients with hyper-protein anomalies or receiving albumin therapies should have protein determinations performed to ensure that elevated albumin levels are not a source of interference with the EAA™.

EN

5.5.2 Precision

Whole blood samples, neat and with added exogenous endotoxin, were analyzed in eight replicates on each of three instruments. Both the within-run and total precision was calculated according to NCCLS procedures in EP5-A.

Sample	N	Mean EAA™	Within Run		Total Precision	
			1SD	%CV	1SD	%CV
1	24	0.11	0.015	14	0.023	22
2	24	0.20	0.030	15	0.029	14
3	24	0.30	0.043	15	0.042	14
4	24	0.50	0.059	12	0.064	13
5	24	0.52	0.036	7	0.034	6
6	24	0.59	0.050	8	0.046	8

5.5.3 Analytical Sensitivity

A low EAA patient sample was used to estimate the sensitivity of the EAA method. Twenty-four EAA determinations were made on a single patient sample with a low EAA level; eight measurements on each of three instruments. The mean EAA level of the patient was 0.11 with a SD of 0.023. Sensitivity, by the two standard deviation method, is thus estimated at EAA result of 0.046.

5.5.4 Analytical Specificity

Endotoxin from the Gram-negative bacteria, E. coli O55:B5, P. aeruginosa, K. pneumoniae, S. enterides, E. coli 0127:B8 and S. marcescens, and S. flexnevi induced an increase in EAA™ levels of 0.10 to 0.52 when 100 pg/mL of the endotoxin was added to a blood sample with EAA result of 0.33. In addition, preparations of V. cholerae LPS produced statistically significant increases in EAA results. No reactivity is seen with up to 2000 pg/mL lipoteichoic acid extracts from the following strains of Gram-positive bacteria: S. mutans, S. pyogenes,

EN

S. sanguis, S. faecalis, S. aureus and B. subtilis. Similarly, there was no reactivity of yeast mannan up to 2000 pg/mL and cell wall extracts of C. albicans and Aspergillus fumigatus.

5.5.5 Linearity of Results

The assay has been optimized to be highly sensitive to low levels of endotoxin and to resist a high dose hook effect induced by grossly high endotoxin levels. Therefore, the curve is curvilinear in shape. When patient samples contain low levels of endotoxin, small increases in endotoxin will result in significant increases in EAA results. Dilutions are not required for samples that have generated high EAA results.

6. Storage and Stability

Store the EAA™ kit and its components (in the original sealed package) at 2-25°C. Do not use the EAA™ kit beyond the expiration date.

7. References:

1. Van Deventer SJH, et al. Endotoxemia: an early predictor of septicemia in febrile patients. The Lancet (March 19) 1988; 605-609.
2. Parrillo JE, Parker MM, Natanson C, et al. Septic shock in humans: advances in the understanding of pathogenesis, cardiovascular dysfunction, and therapy. Ann Int Med 1990; 113: 227-242.
3. Zhao B, Bowden RAS, Stavchansky SA, et al. Human endothelial cell response to gram-negative lipopolysaccharide assessed with cDNA microarrays. Am J Cell Physiol 2001; 281: C1587-C1595.
4. Romaschin AD, et al. A rapid assay of endotoxin in whole blood using autologous neutrophil dependent chemiluminescence. J. of Immunology. Methods 1998; 212: 169-185.
5. NCCLS (National Committee for Clinical Laboratory Standards, 711 East Lancaster Avenue, Villanova, PA 19085) Document # M29-A Protection of Laboratory Worker from Instrument Biohazards and Infectious Disease Transmitted by Blood, Body Fluids and Tissues; Approved Guideline, 1997.

EN

6. NCCLS (National Committee for Clinical Laboratory Standards, 711 East Lancaster Avenue, Villanova, PA 19085) Document # H3-#a Procedures for the Collection of Diagnostic Blood Specimens by Venipuncture – Third Edition; Approved Standard, 1991.

FOR IN VITRO DIAGNOSTIC USE ONLY

CE

Manufactured by:

Spectral Medical Inc.
135 The West Mall
Toronto, ON, Canada
M9C 1C2
+ 1.416.626.3233
FAX: + 1.416.626.7383
www.spectraldx.com

Authorized Representative for Europe:

Dr. Rolf Keck
Ritterstraße 25
D-77977 Rust/Baden
Germany
+ 49.782.286.5111
FAX: + 49.782.286.5113

Endotoxin Activity Assay (EAA™) is a registered trademark of Spectral Medical Inc.

Dosage de l'activité de l'endotoxine (EAA™)

Pour diagnostic in vitro uniquement

FR

1. Utilisation Prévue

Le dosage EAA™ est un test diagnostique in vitro rapide qui utilise un anticorps monoclonal spécifique pour mesurer l'activité de l'endotoxine dans des échantillons de sang total humain prélevé sur EDTA.

Ces informations, employées en association avec des cultures microbiennes et d'autres tests diagnostiques pertinents (par ex. échographie, bronchoscopie ou tomodensitométrie), ainsi que les informations cliniques (signes cliniques et résultats d'examens radiographiques) peuvent :

- Contribuer à l'évaluation chez les patients des risques de développement d'une sepsie grave.
- Orienter l'utilisation d'un traitement anti-endotoxine spécifique.

2. Dosage de l'activité de l'endotoxine - Introduction

Le dosage EAA permet de mesurer le niveau d'activité de l'endotoxine dans l'échantillon de sang d'un patient de manière semi-quantitative, en distinguant 3 groupes : activité de l'endotoxine faible, intermédiaire et élevée.

L'endotoxine ou lipopolysaccharide (LPS) est un constituant majeur de la paroi cellulaire des bactéries Gram négatives et elle constitue le principal produit bactérien Gram négatif responsable de la sepsie grave[1-2]. Des niveaux élevés d'endotoxine provoquent des modifications de l'expression de plus de 300 gènes, l'activation des macrophages, des neutrophiles, des cellules endothéliales et de la cascade de coagulation, qui mènent à la cascade de la sepsie[3].

Ce dosage utilise la réponse biologique des neutrophiles présents dans le sang d'un patient vis-à-vis d'un complexe immunologique constitué d'endotoxine et d'un anticorps exogène comme mesure de l'activité de l'endotoxine chez ce patient[4]. Le dosage réagit de manière spécifique à la chaîne de lipide A de la LPS des bactéries à Gram négatif et ne provoque pas de réaction croisée avec les composants de la paroi cellulaire des bactéries à Gram positif et autres micro-organismes.

FR

3. Contenus

3.1 Kit EAA™

Nom	Quantité
EAAST-20	1
Plateaux EAA™	20
Plateaux de contrôle qualité EAA™	1
Flacons de solution EAA™	21
Instructions d'utilisation	1

3.2 Plateaux EAA™

Nom	Quantité	Description
Tube 1	2	Stabilisateurs et luminol-zymosan
Tube 2	2	Anticorps murin anti-endotoxine monocloral, stabilisateurs et luminol-zymosan
Tube 3	2	Anticorps murin anti-endotoxine monocloral, stabilisateurs et luminol-zymosan
Tube max LPS	1	Endotoxine (E. coli O55:B5) avec stabilisateurs
Tube aliquote	1	Tube vide

FR

3.3 Plateaux de Contrôle Qualité EAA™

Nom	Quantité	Description
Tube 1	2	Stabilisateurs et luminol-zymosan
Tube de contrôle faible	1	Anticorps murin monocloral, stabilisateurs et luminol-zymosan
Tube de contrôle élevé	1	Anticorps murin anti-endotoxine monocloral, stabilisateurs et luminol-zymosan
Tube 3	2	Anticorps murin anti-endotoxine monocloral, stabilisateurs et luminol-zymosan
Tube max LPS CQ	1	Endotoxine (E. coli 055:B5) avec stabilisateurs
Tube aliquote CQ	1	Tube vide

3.4 Solution EAA™

Nom	Quantité	Description
Solution EAA™	8 mL/flacon	Solution saline équilibrée de Kank et solution d'héparine

3.5 Accessoires

Non fournis avec le kit.

- Combipipette
- Micropipette 500 µL
- Embouts de pipette stériles pouvant délivrer 40 uL et 1000 uL
- Embouts de pipette 1000 uL
- Tubes EDTA de prélèvement sanguin
- Chronomètre
- Luminomètre (Berthold Smartline TL Tube ou équivalent)
- Agitateur incubateur pouvant incuber des échantillons à 37 °C et agiter à 100 t/min
- Agitateur-mélangeur vortex

FR

4. Précautions

- Suivre les précautions générales de laboratoire pour la manipulation et le stockage du matériel de dosage non ouvert.
- Le kit EAA™ contient des perles de réactifs lyophilisés dans des tubes à essai en verre borosilicaté. Dans certaines conditions (emballage abîmé, mauvaise étanchéité des pochettes ou mauvais stockage), les perles peuvent rétrécir sous l'effet d'une humidité excessive. Ne pas utiliser les tubes contenant des perles rétrécies car les résultats des tests pourraient être erronés.
- Utiliser des embouts de pipette individuels afin d'éliminer les contaminations lors du pipetage manuel des échantillons ou des contrôles des résidus.
- Ne pas utiliser après la date d'expiration indiquée sur l'étiquette.
- Suivre les précautions universelles recommandées[5] pour la manipulation des solutions usagées et des échantillons humains.
- Manipuler et éliminer les solutions EAA™ usagées et tous les produits sanguins humains en suivant les précautions universelles.
- Ne pas pipeter les solutions avec la bouche.
- Nettoyer immédiatement les éclaboussures avec une solution d'hypochlorite de sodium à 0,5 %.

5. Principe

Une mesure de l'activité initiale (Tube 1) en l'absence de l'anticorps anti-endotoxine spécifique permet de connaître la stimulation non spécifique du métabolisme oxydatif des neutrophiles du patient. Une mesure de contrôle supplémentaire comportant l'anticorps anti-endotoxine spécifique et un excès d'endotoxine exogène (Tube 3) permet de connaître la stimulation maximum du métabolisme oxydatif des neutrophiles du patient. La mesure test (Tube 2) comporte l'anticorps spécifique pour mesurer le niveau réel de l'activité de l'endotoxine. Les résultats d'EAA sont calculés en normalisant la chimiluminescence des échantillons (Tube 2) par rapport à la chimiluminescence maximale (Tube 3), en corrigeant les deux mesures selon la chimiluminescence de l'activité basale (Tube 1).

FR

5.1 Prélèvement et Préparation des Échantillons

Utiliser uniquement des tubes de prélèvement sanguin contenant un anti-coagulant (EDTA) pour obtenir des échantillons de sang total pour cette procédure. Les directives recommandées par le NCCLS[6] doivent être respectées lors du prélèvement, du transport et du traitement des échantillons de patient. Les échantillons doivent être recueillis dans des tubes à prélèvement sanguin contenant un anti-coagulant, l'EDTA, par ponction veineuse ou via un cathéter à demeure. Si le prélèvement se fait au moyen de canules ou cathéters artériels ou veineux, rincer les tuyaux conformément à la politique de votre établissement avant de prélever le sang dans un tube contenant un anti-coagulant (EDTA). Les échantillons de sang peuvent être conservés jusqu'à 180 minutes à température ambiante entre 18 et 25 °C avant analyse. Le sang doit être soigneusement mélangé par inversion pendant au moins 20 à 30 secondes immédiatement avant l'analyse.

Il est recommandé de prélever chez le patient au minimum 1,5 mL de sang total pour effectuer l'examen du patient (1,0 mL requis). Il est recommandé d'utiliser au minimum 1,5 mL de sang total pour effectuer l'examen de CQ (1,0 mL requis). Le sang utilisé pour le test de CQ peut provenir d'un échantillon de sang total indépendant.

5.2 Mode d'emploi avec Luminomètre Berthold Smartline TL Tube

Remarque: Avant de commencer le dosage EAA™ ou le test de CQ EAA™, assurez-vous que le luminomètre Berthold SmartLine TL Tube est allumé (« ON ») et que l'agitateur incubateur est allumé (« ON ») et chauffé à 37 °C ± 1,0 °C.

5.2.1 Procédure de test EAA™

Les bonnes pratiques de laboratoire recommandent d'exécuter deux fois le dosage EAA pour chaque patient. La moyenne des deux dosages est utilisée pour le rapport des résultats EAA™. Chaque laboratoire doit développer et appliquer des CV acceptables pour déterminer l'acceptation des mesures en double. Vous pouvez traiter jusqu'à 3 échantillons de patient en même temps.

FR

1. Placez les tubes EAA™ de chaque échantillon de patient dans le portoir de tubes fourni et enlevez les bouchons.
2. À l'aide d'une combipipette, placez 1,0 mL de la solution EAA™ dans les tubes 1, 2 et 3 (en double).
3. Après avoir mélangé l'échantillon sanguin du patient par inversion, pipetez des aliquotes de 0,5 ml directement dans le tube Aliquot et le tube LPS MAX Tube. Agitez le tube LPS MAX au vortex.
4. Placez le(s) portoir(s) dans l'agitateur incubateur, fermez le couvercle et incubez pendant 10 minutes.
5. Après une incubation de 10 minutes, ouvrez le couvercle, agitez le tube Aliquot au vortex puis, à l'aide d'une combipipette, placez 40 µl de sang dans les tubes 1 et 2 (en double) en utilisant un embout pour combipipette stérile.
6. Agitez le tube LPS MAX au vortex, puis à l'aide d'une combipipette, placez 40 µl de sang dans les tubes 3 (en double) en utilisant un embout pour combipipette stérile.
7. Agitez les tubes 1, 2 et 3 au vortex et replacez-les dans le(s) portoir(s) dans l'agitateur incubateur, fermez le couvercle et lancez le mouvement (réglé sur 100 t/mn) et le chronomètre (réglé sur 14 minutes).
8. Insérez la carte à puce étiquetée EAA dans le luminomètre et appuyez sur Démarrer.
9. Après une incubation de 14 minutes, suivez les instructions qui s'affichent sur l'écran du luminomètre afin de lire les tubes EAA dans le bon ordre.
10. Agitez chaque tube doucement au vortex, ouvrez le tiroir des échantillons et placez le tube dans le porte-échantillon, fermez le tiroir des échantillons et attendez la lecture RLU. Répétez l'opération pour le tube suivant.
11. Après lecture de tous les tubes, les résultats du dosage EAA sont imprimés automatiquement sur la sortie papier jointe.
12. Répétez l'opération pour chaque échantillon de patient si nécessaire.

FR

5.2.1.1 Interprétation des Résultats

Intervalle (résultat EAA™)	Interprétation des résultats
0,0 à 0,39	Un niveau d'endotoxine faible représente un faible risque de développement d'une sepsie grave.
0,40 à 0,59	un niveau intermédiaire d'activité de l'endotoxine indique un risque élevé de progression vers une sepsie grave.
≥ 0,60	Un niveau élevé d'activité de l'endotoxine indique un risque élevé de développement d'une sepsie grave.

5.2.2 Procédure de test de CQ EAA™

Il est recommandé d'effectuer un test de CQ pour chaque kit EAA™. Cependant, chaque laboratoire doit établir son propre schéma de test de CQ en fontion des normes locales.

1. Placez les tubes CQ dans le portoir fourni et enlevez les bouchons.
2. À l'aide d'une combipipette, placez 1,0 mL de la solution EAA™ dans chaque tube.
3. Pipetez des aliquotes de 0,5 ml de sang total directement dans les tubes Aliquot CQ et LPS MAX CQ. Agitez le tube LPS MAX CQ au vortex.
4. Placez le portoir dans l'agitateur incubateur, fermez le couvercle et incubez pendant 10 minutes.
5. Après une incubation de 10 minutes, ouvrez le couvercle, agitez le tube Aliquot CQ au vortex puis, à l'aide d'une combipipette, placez 40 µl de sang dans le tube 1 et le tube Contrôle Bas en utilisant un embout pour combipipette stérile.
6. Agitez le tube LPS MAX CQ au vortex, puis, à l'aide d'une combipipette, placez 40 µl de sang tout d'abord dans le tube Contrôle Haut puis dans le tube 3 en utilisant un embout pour combipipette stérile.
7. Agitez tous les tubes au vortex et replacez-les dans le portoir dans l'agitateur incubateur et lancez le mouvement (réglé sur 100 t/mn) et le chronomètre (réglé sur 14 minutes).

8. Insérez la carte à puce étiquetée EAA CQ dans le luminomètre et appuyez sur Démarrer.
9. Après une incubation de 14 minutes, suivez les instructions qui s'affichent sur l'écran du luminomètre afin de lire les tubes EAA CQ dans le bon ordre.
10. Agitez chaque tube doucement au vortex, ouvrez le tiroir des échantillons et placez le tube dans le porte-échantillon, fermez le tiroir des échantillons et attendez la lecture RLU. Répétez l'opération pour le tube suivant.
11. Après lecture de tous les tubes, les résultats du dosage EAA™ CQ sont imprimés automatiquement sur la sortie papier jointe.

5.2.2.1 Interprétation des Résultats

Le CQ de la solution EAA™ est confirmé avec les résultats suivants:

Interprétation des résultats	Résultat EAA™
CQ Bas EAA	0,00 – 0,20
CQ Haut EAA	0,80 – 1,50

Si le résultat du test de CQ pour un contrôle faible ou élevé ne fournit pas les résultats escomptés, il convient de répéter le test. Si les résultats du nouveau test de CQ demeurent en dehors des limites acceptables, il se peut que les résultats EAA consécutifs ne soient pas valables. Veuillez en informer votre représentant EAA™ dès que possible.

5.3 Limites

5.3.1 Analytiques

- Comme pour tous les immunodosages ou réactions immunochimiques il convient de procéder avec le plus grand soin.
- L'utilisateur doit être avertit des effets possibles sur les résultats des interférences potentielles dues à des médicaments ou à des substances endogènes inconnues. Voir Substances interférentes ci-dessous.

FR

5.3.2 Cliniques

- Les résultats du dosage EAA™ doivent être évalués dans le contexte des résultats biologiques du patient et en fonction de son état clinique global. Dans le cas où les résultats biologiques sont en désaccord avec le tableau clinique ou les antécédents du patient, des tests supplémentaires doivent être mis en œuvre.
- Des observations de résultats positifs vis-à-vis de l'endotoxine chez des patients ne présentant pas de sepsie grave ont déjà été rapportées dans des études publiées.
- Les résultats obtenus sur des patients en dehors du cadre des soins intensifs doivent être interprétés avec prudence car ils peuvent ne pas être d'une valeur clinique significative .

5.4 Performances Cliniques

L'essai Multi-Center Endotoxin Detection in Critical illness (MEDIC) est une étude observationnelle, multicentrique, prospective, réalisée dans 10 services de soins intensifs (ICU) d'hôpitaux universitaires en Amérique du Nord et en Europe. La présence d'une endotoxémie a été évaluée lors du premier jour de séjour des patients dans le service de soins intensifs afin de déterminer les chances de développer une sepsie grave au cours des 24 heures qui suivent l'admission dans le service de soins intensifs. La population cible de l'Étude d'évaluation des risques comportait tous les patients éligibles enrôlés dans l'essai MEDIC lors du premier jour d'admission dans le service de soins intensifs admission qui disposaient d'échantillons évaluables, N=857. Les données démographiques des patients sont présentées dans le tableau suivant.

Données démographiques et caractéristiques initiales des patients analysés

Caractéristiques	
Âge : moyenne (E-T) médiane	60 (±17) 62 [49, 74]
Sexe (% masculin)	58,9%
Race (% non caucasien)	15,8%
APACHEII : moyenne (E-T)	15,2 (±9,5) 14 [8, 21]
Durée du séjour dans l'ICU : moyenne (E-T)	5,4 (±10,9) 2,0 [1, 5]
Durée du séjour à l'hôpital : moyenne	23,3 (±27,0) 14,0 [7, 29]
Mortalité dans l'ICU	13,3%
Mortalité à l'hôpital	19,9%

La population des patients en soins intensifs constitue l'environnement clinique le plus approprié pour l'évaluation du dosage EAA™. L'utilité du dosage EAA™ n'a été validée que pour la population des patients en soins intensifs. La relation entre les niveaux d'endotoxine et le risque de développement d'une sepsie grave chez les patients gravement malades est destinée à apporter un complément aux informations cliniques actuellement disponibles afin d'établir le risque de sepsie grave au cours du premier jour d séjour dans les services de soins intensifs.

5.5 Caractéristiques de Performance

5.5.1 Substances Interférentes

- Des taux de triglycérides jusqu'à 1000 mg/dl n'interfèrent pas avec le dosage EAA. En cas de concentrations de triglycérides supérieures à 1500 mg/dL, une diminution des résultats EAA a été démontrée. Les échantillons fortement lipémiques ne doivent pas être utilisés pour les dosages EAA™.
- Un taux d'hémoglobine de 100 mg/dL n'interfère pas avec les mesures EAA tandis qu'un taux de 500 mg/dL atténue les mesures EAA™ faibles de moins de 0,03 % et les mesures EAA élevées de 15 % en moyenne. Il convient d'éviter l'utilisation d'échantillons grossièrement hémolysés dans le cadre de l'EAA™.
- Une perfusion exogène d'albumine exempte d'endotoxine chez des patients à des niveaux qui font augmenter la concentration

FR

plasmatique en albumine de plus de 10 g/l au-dessus de la limite normale supérieure sont susceptibles de faire diminuer les valeurs du dosage EAA. Les échantillons provenant de patients porteurs d'anomalies hyperprotéiniques ou recevant des traitements par l'albumine doivent faire l'objet d'une mesure des protéines afin de s'assurer que les concentrations élevées en albumine ne constituent par une source d'interférences avec le dosage EAA™.

5.5.2 Précision

Les échantillons de sang total, nets et additionnés d'endotoxine endogène, ont été analysés à huit reprises chacun sur trois instruments La répétabilité et la reproductibilité ont été calculées conformément aux procédures du NCCLS, EP5-A.

Échantillon	N	Moyenne EAA™	Dans	Opérer	Totale Précision	
			1SD	%CV	1E-T	%CV
1	24	0,11	0,015	14	0,023	22
2	24	0,20	0,030	15	0,029	14
3	24	0,30	0,043	15	0,042	14
4	24	0,50	0,059	12	0,064	13
5	24	0,52	0,036	7	0,034	6
6	24	0,59	0,050	8	0,046	8

5.5.3 Sensibilité Analytique

Un échantillon de patient présentant un faible niveau d'EAA a été utilisé pour estimer la sensibilité de la méthode EAA. Vingt-quatre déterminations EAA™ ont été réalisées sur un échantillon de patient unique présentant un niveau faible de EAA™; huit mesures sur chacun des trois instruments. Le niveau moyen du dosage EAA™ du patient était de 0,11 avec un E-T de 0,023. Le sensibilité, calculée par la méthode des deux écart- types, a été ainsi estimée à un résultat EAA de 0,046.

FR

5.5.4 Spécificité Analytique

L'endotoxine des bactéries à Gram négatif, E. coli O55:B5, P. aeruginosa, K. pneumoniae, S. enterides, E. coli 0127:B8 et S. marcescens, et S. flexnevi, a entraîné une augmentation des niveaux d'EAA™ de 0,10 à 0,52 lorsque 100 pg/mL de l'endotoxine étaient ajoutés à un échantillon sanguin présentant un résultat d'EAA de 0,33. De plus, les préparations de LPS de V. cholerae ont entraîné des augmentations statistiques considérables dans les résultats EAA. Aucune réactivité n'a été observée jusqu'à 2000 pg/m d'extraits d'acide lipotéichoïque provenant des souches suivantes de bactéries Gram positives : S. mutans, S. pyogenes, S. sanguis, S. faecalis, S. aureus et B. subtilis. De même, il n'a été observé aucune réactivité avec le mannane des levures jusqu'à 2000 pg/ml et avec les extraits de paroi cellulaire de C. albicans et A. fumagatis.

5.5.5 Linéarité des Résultats

Le dosage a été optimisé pour être hautement sensible à de faibles niveaux d'endotoxine et pour résister à un effet crochet à haute dose induit par des niveaux extrêmement élevés d'endotoxine.
Par conséquent, la courbe présente un aspect curvilinéaire.
Lorsque les échantillons de patients présentent de faibles niveaux d'endotoxine, de légères augmentations de l'endotoxine entraînent des augmentations considérables dans les résultats EAA. Il n'est pas nécessaire de procéder à des dilutions pour les échantillons qui ont généré des résultats élevées du dosage EAA.

6. Stockage et Stabilité

Conserver le kit EAA™ et ses composants (dans l'emballage d'origine) à une température comprise entre 2 et 25 °C. Ne pas utiliser le kit EAA™ après la date d'expiration.

FR

7. References:

1. Van Deventer SJH, et al. Endotoxemia: an early predictor of septicemia in febrile patients. The Lancet (March 19) 1988; 605-609.
2. Parrillo JE, Parker MM, Natanson C, et al. Septic shock in humans: advances in the understanding of pathogenesis, cardiovascular dysfunction, and therapy. Ann Int Med 1990; 113: 227-242.
3. Zhao B, Bowden RAS, Stavchansky SA, et al. Human endothelial cell response to gram-negative lipopolysaccharide assessed with cDNA microarrays. Am J Cell Physiol 2001; 281: C1587-C1595.
4. Romaschin AD, et al. A rapid assay of endotoxin in whole blood using autologous neutrophil dependent chemiluminescence. J. of Immunology. Methods 1998; 212: 169-185.
5. NCCLS (National Committee for Clinical Laboratory Standards, 711 East Lancaster Avenue, Villanova, PA 19085) Document # M29-A Protection of Laboratory Worker from Instrument Biohazards and Infectious Disease Transmitted by Blood, Body Fluids and Tissues; Approved Guideline, 1997.
6. NCCLS (National Committee for Clinical Laboratory Standards, 711 East Lancaster Avenue, Villanova, PA 19085) Document # H3-#a Procedures for the Collection of Diagnostic Blood Specimens by Venipuncture – Third Edition; Approved Standard, 1991.

POUR DIAGNOSTIC IN VITRO UNIQUEMENT

CE

FR

Fabriqué par:

Spectral Medical Inc.
135 The West Mall
Toronto, ON, Canada
M9C 1C2
+ 1.416.626.3233
FAX: + 1.416.626.7383
www.spectraldx.com

Représentant agréé pour l'Europe:

Dr. Rolf Keck
Ritterstraße 25
D-77977 Rust/Baden
Allemagne
+ 49.782.286.5111
FAX: + 49.782.286.5113

EAA™ (dosage de l'activité de l'endotoxine) est une marque commerciale de Spectral Medical Inc.

Endotoxin Activity Assay (EAA™)

Nur Für Die In-vitro-Diagnostik

DE

1. Verwendungszweck

Der EAA™ ist ein in-vitro-diagnostischer Schnelltest, der mit einem spezifischen monoklonalen Antikörper die Endotoxinaktivität in EDTA-Vollblutproben misst.

Diese Informationen können bei Verwendung in Verbindung mit mikrobiellen Kulturen und anderen relevanten diagnostischen Untersuchungen (z.B. Ultraschall, Bronchoskopie oder CT) und klinischen Informationen (klinische Anzeichen und/oder röntgenologische Ergebnisse):

- bei der Risikoanalyse von Patienten für die Entwicklung einer schweren Sepsis helfen.
- die Anwendung einer bestimmten Anti-Endotoxin-Therapie steuern.

2. Endotoxin Activity Assay - Einleitung

Der EAA-Test dient zur semi-quantitativen Beurteilung der Endotoxinaktivität in der Blutprobe eines bestimmten Patienten, wobei zwischen 3 Gruppen unterschieden wird: niedrige, intermediäre und hohe Endotoxinaktivität.

Endotoxin bzw. Lipopolysaccharid (LPS) ist ein Hauptbestandteil der Zellwand von gramnegativen Bakterien und das wichtigste Produkt gramnegativer Bakterien, das für die schwere Sepsis verantwortlich ist.[1-2] Erhöhte Endotoxinspiegel verursachen Veränderungen in der Expression von mehr als 300 Genen und aktivieren Makrophagen, Neutrophile, Endothelzellen und die Gerinnungskaskade, was schließlich zur Auslösung der Sepsiskaskade führt.[3]

Dieser Test nutzt die biologische Reaktion der Neutrophilen im Patientenblut auf den immunologischen Komplex von Endotoxin und exogenem Antikörper zur Bestimmung der Endotoxinaktivität des Patienten.[4] Der Test reagiert speziell mit dem Lipid-A-Anteil des LPS gramnegativer Bakterien und findet keine Kreuzreaktion mit den Zellwandbestandteilen grampositiver Bakterien und anderer Mikroorganismen.

3. Inhalt

3.1 EAA™ Kit

Name	Anzahl
EAAST-20	1
EAA™-Trays	20
EAA™--Qualitätskontroll-Trays	1
EAA™--Lösungsfläschchen	21
Gebrauchsanleitung	1

3.2 EAA™-Trays

Name	Anzahl	Beschreibung
Röhrchen 1	2	Stabilisatoren und Luminol-Zymosan
Röhrchen 2	2	Muriner monoklonaler Anti-Endotoxin-Antikörper, Stabilisatoren und Luminol-Zymosan
Röhrchen 3	2	Muriner monoklonaler Anti-Endotoxin-Antikörper, Stabilisatoren und Luminol-Zymosan
LPS-Max-Röhrchen	1	Endotoxin (E. coli O55:B5) mit Stabilisatoren
Aliquot-Röhrchen	1	Leeres Röhrchen

DE

3.3 EAA™- Qualitätskontroll Trays

Name	Anzahl	Beschreibung
Röhrchen 1	2	Stabilisatoren und Luminol-Zymosan
Röhrchen Low Control	1	Muriner monoklonaler Antikörper, Stabilisatoren und Luminol-Zymosan
Röhrchen High Control	1	Muriner monoklonaler Anti-Endotoxin-Antikörper, Stabilisatoren und Luminol-Zymosan
Röhrchen 3	2	Muriner monoklonaler Anti-Endotoxin-Antikörper, Stabilisatoren und Luminol-Zymosan
QC LPS-Max-Röhrchen	1	Endotoxin (E. coli „055:B5“) mit Stabilisatoren
QC Aliquot-Röhrchen	1	Leeres Röhrchen

3.4 EAA™- Lösung

Name	Anzahl	Beschreibung
EAA™ Lösung	8 mL/Fläschchen	HBSS und Heparin-Lösung

3.5 Zubehör

Nicht im Kit inbegriffen.

- Kombipipette
- 500-µL-Mikropipette
- Sterile Kombipipettenspitzen mit Pipettierkapazität 40 uL und 1000 uL
- 1000-uL-Mikropipettenspitzen
- EDTA-Blutentnahme-Röhrchen
- Zeitmesser
- Luminometer (Berthold SmartLine TL Tube oder äquivalent)
- Inkubationsschüttler, mit dem Proben bei 37 °C inkubiert und bei 100 U/min agitiert werden können
- Vortexmischer

4. Vorsichtsmaßnahmen

- Bei der Handhabung und Lagerung der ungeöffneten Assaymaterialien sind die allgemeinen laborüblichen Vorsichtsmaßnahmen zu beachten.
- Der EAA™ enthält lyophilisierte Reagenzkügelchen in Reagenzröhrchen aus Borosilikatglas. Unter bestimmten Bedingungen (beschädigte Verpackung, unsachgemäß verschlossene Beutel oder unsachgemäße Lagerung) können die Kügelchen bei übermäßiger Feuchtigkeit schrumpfen. Röhrchen, die geschrumpfte Kügelchen enthalten, dürfen nicht verwendet werden, da dies zu falschen Testergebnissen führen kann.
- Beim manuellen Pipettieren für jede Probe und Kontrolle eine neue Pipettenspitze verwenden, um eine Verschleppung von Restsubstanzen zu vermeiden.
- Nicht nach dem auf dem Etikett angegebenen Verfallsdatum verwenden.
- Bei der Handhabung gebrauchter Lösungen und Humanproben sind die empfohlenen allgemeingültigen Vorsichtsmaßnahmen[5] zu beachten.
- Gebrauchte EAA™-Lösung und alle menschlichen Blutprodukte sind unter Anwendung der allgemeingültigen Vorsichtsmaßnahmen zu handhaben und zu entsorgen.
- Lösungen nicht mit dem Mund pipettieren.
- Spritzer sofort mit einer 0,5%-igen Natriumhypochloritlösung aufwischen.

DE

DE

5. Testprinzip

Eine Messung der Basalaktivität (Röhrchen 1) ohne den spezifischen Anti-Endotoxin-Antikörper misst den unspezifischen oxidativen Burst der Neutrophilen des Patienten. Eine zusätzliche Kontrollmessung mit dem spezifischen Anti-Endotoxin-Antikörper und einem Überschuss an exogenem Endotoxin (Röhrchen 3) misst den maximalen oxidativen Burst der Neutrophilen im Patientenblut. Die Testmessung (Röhrchen 2) mit dem spezifischen Antikörper misst die Netto-Endotoxinaktivität. Das EAA-Ergebnis wird durch Normalisierung der Chemilumineszenz in der Testprobe (Röhrchen 2) gegen die maximale Chemilumineszenz (Röhrchen 3) berechnet, wobei beide Messungen um die Chemilumineszenz der Basalaktivität (Röhrchen 1) korrigiert werden.

5.1 Probengewinnung und – Vorbereitung

Es dürfen nur EDTA-antikoagulierte Blutentnahmeröhrchen zur Entnahme von Vollblutproben für dieses Verfahren benutzt werden. Entnahme, Transport und Bearbeitung der Patientenproben sollten die Richtlinien der NCCLS[6] beachtet werden. Die Proben müssen durch Venenpunktion oder über einen Verweilkatheter in Blutentnahmeröhrchen, die EDTA-Antikoagulans enthalten, entnommen werden. Wird die Probe durch eine Arterien- oder Venenverweilkanüle bzw. einen Verweilkatheter entnommen, spülen Sie bitte den Zugang den Vorschriften Ihrer Institution entsprechend vor der Entnahme von Blut in das EDTA-antikoagulierte Röhrchen. Die Blutproben können vor der Analyse bis zu 180 Minuten bei einem Umgebungstemperaturbereich von 18-25 °C gelagert werden. Unmittelbar vor der Analyse muss das Blut für mindestens 20-30 Sekunden durch vorsichtiges Umdrehen des Röhrchens gründlich gemischt werden.

Für die Durchführung des Patientenassays wird ein Minimum von 1,5 mL Patienten-Vollblut empfohlen (1,0 mL sind Vorschrift). Für die Durchführung des QK-Tests wird ein Minimum von 1,5 mL Vollblut empfohlen (1,0 mL sind Vorschrift). Das Blut für den QK-Test kann eine unabhängige Vollblutprobe sein.

5.2 Gebrauchsanleitung mit dem Berthold SmartLine TL Tube Luminometer

<u>Hinweis:</u> Vor Beginn des EAA™ oder EAA™ -QK-Tests ist darauf zu achten, dass das Berthold SmartLine TL Tube Luminometer eingeschaltet ist und dass der Inkubatorschüttler eingeschaltet und auf 37 °C ± 1,0 °C erhitzt ist.

5.2.1 EAA™-Testverfahren

Gemäß der guten Laborpraxis sollte der EAA für jeden Patienten zweifach durchgeführt werden. Der Mittelwert des Duplikats wird zur Berichterstattung des Ergebnisses des EAA™ verwendet. Jedes Labor sollte akzeptable VKs für die Akzeptanz von Mehrfachbestimmungen ermitteln und anwenden. Es können bis zu 3 Patientenproben in einem Durchlauf analysiert werden.

1. Die EAA™ Röhrchen für jede Patientenprobe in die bereitgestellten Röhrchenracks stellen und die Verschlusskappen abnehmen.
2. Mit der Kombipipette 1,0 mL der EAA™-Lösung in die Röhrchen 1, 2 und 3 pipettieren (zweifach).
3. Nach Durchmischen der Patientenprobe durch vorsichtiges Umdrehen 0,5-ml-Aliquots direkt in das Aliquot-Röhrchen und das LPS-MAX-Röhrchen pipettieren. Das LPS-MAX-Röhrchen vortexen.
4. Das (die) Rack(s) in den Inkubationsschüttler stellen, den Deckel schließen und 10 Minuten inkubieren.
5. Nach der 10-minütigen Inkubation den Deckel öffnen, das Aliquot-Röhrchen vortexen und anschließend mit der Kombipipette und einer sterilen Pipettenspitze 40 µl Blut in die Röhrchen 1 und 2 (jeweils doppelt) pipettieren.
6. Das LPS-MAX-Röhrchen vortexen, anschließend mit der Kombipipette und einer sterilen Pipettenspitze 40 µl Blut in Röhrchen 3 (doppelt) pipettieren.
7. Die Röhrchen 1, 2 und 3 vortexen und wieder in das(die) Rack(s) im Inkubationsschüttler stellen, den Deckel schließen und den

DE

Schüttler (eingestellt auf 100 rpm) und den Zeitmesser (eingestellt auf 14 Minuten) starten.

8. Die EAA-Chipkarte in das Luminometer einführen und Start drücken.
9. Nach der 14-minütigen Inkubation die Anweisungen auf dem Luminometer-Display zur Messung der EAA-Röhrchen in der richtigen Reihenfolge beachten.
10. Jedes Röhrchen vorsichtig vortexen, die Probenschublade öffnen und das Röhrchen in den Probenhalter einsetzen, die Probenschublade schließen und warten, bis eine RLU-Messung durchgeführt wird. Den Vorgang mit dem nächsten Röhrchen wiederholen.
11. Wenn alle Röhrchen gemessen sind, werden die EAA-Testergebnisse automatisch ausgedruckt.
12. Die Analyse für jede weitere Patientenprobe wiederholen.

DE

5.2.1.1 Interpretation der Ergebnisse

Bereich (EAA™- Ergebnis)	Interpretation der Ergebnisse
0,0 bis 0,39	Niedrige Endotoxinaktivität bedeutet ein niedriges Risiko für die Entwicklung einer schweren Sepsis.
0,40 bis 0,59	Intermediäre Endotoxinaktivität erhöhtes Risiko für schwere Sepsis.
≥ 0,60	Hohe Endotoxinaktivität hohes Risiko für die Entwicklung einer schweren Sepsis.

5.2.2 EAA™- QK-Testverfahren

Es wird empfohlen, den QK-Test einmal pro EAA™ Kit-Box durchzuführen. Jedes Labor kann jedoch seine eigene QK-Testregelung gemäß den lokalen Richtlinien festlegen.

1. Die QK-Röhrchen in ein bereitgestelltes Rack stellen und die Verschlusskappen abnehmen.
2. Mit der Kombipipette 1,0 mL der EAA™ -Lösung in jedes Röhrchen pipettieren.
3. 0,5-ml-Vollblut-Aliquots direkt in das QC-Aliquot-Röhrchen und das Röhrchen QC LPS MAX pipettieren. Das Röhrchen QC LPS MAX vortexen.
4. Das Rack in den Inkubationsschüttler stellen, den Deckel schließen und 10 Minuten inkubieren.
5. Nach der 10-minütigen Inkubation den Deckel öffnen, das QC-Aliquot-Röhrchen vortexen und anschließend mit der Kombipipette und einer sterilen Pipettenspitze 40 µl Blut in beide Tube-1-Röhrchen und das Röhrchen Low Control pipettieren.
6. Das Röhrchen QC LPS MAX vortexen, anschließend mit der Kombipipette und einer sterilen Pipettenspitze 40 µl Blut zuerst in das Röhrchen High Control und anschließend in beide Tube-3-Röhrchen pipettieren.
7. Alle Röhrchen vortexen und wieder in das Rack im Inkubationsschüttler stellen und den Schüttler (eingestellt auf 100 rpm) und den Zeitmesser (eingestellt auf 14 Minuten) starten.
8. Die EAA-QC-Chipkarte in das Luminometer einführen und Start drücken.
9. Nach der 14-minütigen Inkubation die Anweisungen auf dem Luminometer-Display zur Messung der EAA™-QK-Röhrchen in der richtigen Reihenfolge beachten.
10. Jedes Röhrchen vorsichtig vortexen, die Probenschublade öffnen und das Röhrchen in den Probenhalter einsetzen, die Probenschublade schließen und warten, bis eine RLU-Messung durchgeführt wird. Den Vorgang mit dem nächsten Röhrchen wiederholen.
11. Wenn alle Röhrchen gemessen sind, werden die EAA™-QK-Ergebnisse automatisch ausgedruckt.

DE

5.2.2.1 Interpretation der Ergebnisse

EAA™-Lösungs-QK wird mit den folgenden Ergebnissen bestätigt:

Interpretation der Ergebnisse	EAA™-Ergebnis
EAA Niedrige QK	0,00-0,20
EAA Hohe QK	0,80-1,50

Falls die wiederholten QK-Testergebnisse weiterhin außerhalb der akzeptablen Grenzen liegen, können die anschließenden EAA™-Ergebnisse ungültig sein. Verständigen Sie bitte so bald wie möglich Ihren EAA™ Kundenberater.

DE

5.3 Grenzen

5.3.1 Analytisch

- Wie bei allen Immunoassays oder immunchemischen Reaktionen ist eine sorgfältige Technik erforderlich.
- Der Anwender sollte auf die möglichen Auswirkungen potenzieller Störungen durch Medikamente oder unbekannte endogene Substanzen auf die Ergebnisse achten. Siehe Abschnitt Interferierende Substanzen.

5.3.2 Klinisch

- Die Ergebnisse des EAA™ sollten im Zusammenhang mit allen Laborbefunden und dem klinischen Gesamtzustand des Patienten beurteilt werden. Falls die Laborergebnisse nicht mit dem klinischen Bild oder Verlauf übereinstimmen, sollten weitere Tests durchgeführt werden.
- In publizierten Studien wurden positive Endotoxin-Ergebnisse bei Patienten ohne schwere Sepsis berichtet.
- Ergebnisse außerhalb der kritischen Pflege Einstellung auf Patienten erhalten sollten mit Vorsicht interpretiert werden, da sie nicht von einem signifikanten klinischen Wert sein kann.

5.4 Klinische Leistungsfähigkeit

Die Multi-Center Endotoxin Detection in Critical Illness (MEDIC) Studie war eine multizentrische, prospektive Beobachtungsstudie, die in 10 Intensivstationen von Universitätskliniken in Nordamerika und Europa durchgeführt wurde. Das Vorliegen einer Endotoxämie wurde am ersten Tag des Aufenthalts der Patienten auf der Intensivstation untersucht, um die Wahrscheinlichkeit der Entwicklung einer schweren Sepsis innerhalb von 24 Stunden nach Aufnahme in die Intensivstation zu bestimmen. Die Zielpopulation für die Studie zur Risikoabschätzung beinhaltete alle in Frage kommenden Patienten, die am ersten Tag ihrer Aufnahme in die Intensivstation in die MEDIC-Studie aufgenommen wurden und auswertbare Proben hatten (n=857). Die demografischen Merkmale der Patienten sind in der folgenden Tabelle dargestellt.

DE

Demografische und Ausgangsmerkmale der analysierten Patienten

Merkmal	
Alter: Mittelwert (SD) Median	60 (±17) 62 [49, 74]
Geschlecht (% männlich)	58,9%
Ethnische Zugehörigkeit (% Nichtkaukasier)	15,8%
APACHE-II: Mittelwert (SD) Median	15,2 (±9,5) 14 [8, 21]
Tage auf der ICU: Mittelwert (SD)	5,4 (±10,9) 2,0 [1, 5]
Tage im Krankenhaus: Mittelwert	23,3 (±27,0) 14,0 [7, 29]
ICU-Mortalität	13,3%
Krankenhausmortalität	19,9%

Die Population der Intensivpatienten ist die zweckmäßigste klinische Umgebung für die Durchführung des EAA™-Tests. Der Nutzen des EAA™-Tests wurde nur für die Population der Intensivpatienten nachgewiesen. Der Zusammenhang zwischen den Endotoxinspiegeln und dem Risiko der Entwicklung einer schweren Sepsis bei

Intensivpatienten soll als Ergänzung zu den derzeit verfügbaren klinischen Informationen zur Bestimmung des Risikos einer schweren Sepsis am ersten Tag des Aufenthalts auf der Intensivstation dienen.

5.5 Leistungsdaten

5.5.1 Interferierende Substanzen

- Triglyceridspiegel bis zu 1000 mg/dl interferieren nicht mit den EAA-Messungen. Bei Triglyceridspiegeln über 2500 mg/dL wurde eine Abnahme der EAA-Ergebnisse nachgewiesen. Stark lipämische Proben sollten für den EAA™-Test nicht verwendet werden.
- Ein Hämoglobinspiegel von 100 mg/dL interferiert nicht mit den EAA-Messungen, während Hämoglobinspiegel von 500 mg/dL niedrige EAA™-Messungen um weniger als ein EAA-Ergebnis von 0,03 abschwächen und erhöhte EAA-Werte um durchschnittlich 15% abschwächen. Die Verwendung von stark hämolysierten Proben sollte für den EAA™ vermieden werden.
- Die exogene Infusion von endotoxinfreiem Albumin bei Patienten in Konzentrationen, die das Plasmaalbumin um mehr als 10 g/l über die obere Grenze des Normalbereichs erhöhen, kann die EAA-Werte senken. In Proben von Patienten mit Hyperproteinämien oder Patienten, die eine Albumintherapie erhalten, sollten Proteinbestimmungen durchgeführt werden, um sicherzustellen, dass keine erhöhten Albuminspiegel als Störquelle des EAA™ vorliegen.

5.5.2 Präzision

Vollblutproben wurden unbehandelt und versetzt mit exogenem Endotoxin in acht Replikaten auf je drei Geräten analysiert. Es wurde sowohl die Within-Run-Präzision als auch die Gesamtpräzision gemäß den im Dokument EP5-A beschriebenen NCCLS-Verfahren berechnet.

DE

Probe	N	Mittlerer EAA-Wert	Innerhalb Laufen*		Gesamt Präzision*	
			1SD	%CV	1SD	%CV
1	24	0,11	0,015	14	0,023	22
2	24	0,20	0,030	15	0,029	14
3	24	0,30	0,043	15	0,042	14
4	24	0,50	0,059	12	0,064	13
5	24	0,52	0,036	7	0,034	6
6	24	0,59	0,050	8	0,046	8

5.5.3 Analytische Sensitivität

Eine Patientenprobe mit niedrigem EAA-Wert wurde zur Einschätzung der Sensitivität der EAA-Methode verwendet. Vierundzwanzig EAA-Bestimmungen wurden mit einer einzelnen Patientenprobe mit niedrigem EAA-Wert durchgeführt, und zwar acht Messungen auf je drei Geräten. Der mittlere EAA-Wert des Patienten betrug 0,11 mit einer SD von 0,023. Anhand der Methode Standardabweichung mal zwei wird die Sensitivität auf ein EAA-Ergebnis von 0,046 eingeschätzt.

5.5.4 Analytische Spezifität

Endotoxin aus den gramnegativen Bakterien E. coli O55:B5, P. aeruginosa, K. pneumoniae, S. enterides, E. coli 0127:B8 und S. marcescens sowie S. flexnevi induzierte eine Steigerung des EAA™-Spiegels von 0,10 auf 0,52, als 100 pg/mL des Endotoxins zu einer Blutprobe mit einem EAA-Wert von 0,33 hinzugegeben wurden. Außerdem führten Präparate von V. cholerae LPS zu statistisch signifikanten Steigerungen der EAA-Ergebnisse. Bei bis zu 2000 pg/ml Lipoteichonsäureextrakten der folgenden Stämme von grampositiven Bakterien ist keine Reaktivität zu beobachten: S. mutans, S. pyogenes, S. sanguis, S. faecalis, S. aureus und B. subtilis. Auch Hefe-Mannan in einer Konzentration von bis zu 2000 pg/ml und Zellwandextrakte von C. albicans und A. fumigatus zeigten keine Reaktivität.

DE

5.5.5 Linearität der Ergebnisse

Der Test wurde so optimiert, dass er hoch sensitiv auf niedrige Spiegel von Endotoxin ist und keinem High-Dose-Hook-Effekt durch stark erhöhte Endotoxinspiegel unterliegt. Daher ist die Kurve krummlinig. Wenn Patientenproben niedrige Endotoxinspiegel enthalten, führen geringfügige Anstiege des Endotoxins zu starken Anstiegen der EAA-Werte. Verdünnungen sind für Proben, die hohe EAA-Werte erzeugen, nicht erforderlich.

DE

6. Lagerung und Stabilität

Das EAA™-Kit und seine Bestandteile (im original verschlossenen Verpackung) bei 2-25 °C lagern. Das EAA™-Kit darf nicht über sein Verfallsdatum hinaus benutzt werden.

7. Literatur:

1. Van Deventer SJH, et al. Endotoxemia: an early predictor of septicemia in febrile patients. The Lancet (March 19) 1988; 605-609.
2. Parrillo JE, Parker MM, Natanson C, et al. Septic shock in humans: advances in the understanding of pathogenesis, cardiovascular dysfunction, and therapy. Ann Int Med 1990; 113: 227-242.
3. Zhao B, Bowden RAS, Stavchansky SA, et al. Human endothelial cell response to gram-negative lipopolysaccharide assessed with cDNA microarrays. Am J Cell Physiol 2001; 281: C1587-C1595.
4. Romaschin AD, et al. A rapid assay of endotoxin in whole blood using autologous neutrophil dependent chemiluminescence. J. of Immunology. Methods 1998; 212: 169-185.
5. NCCLS (National Committee for Clinical Laboratory Standards, 711 East Lancaster Avenue, Villanova, PA 19085) Document # M29-A Protection of Laboratory Worker from Instrument Biohazards and Infectious Disease Transmitted by Blood, Body Fluids and Tissues; Approved Guideline, 1997.
6. NCCLS (National Committee for Clinical Laboratory Standards, 711 East Lancaster Avenue, Villanova, PA 19085) Document # H3-#a Procedures for the Collection of Diagnostic Blood Specimens by Venipuncture – Third Edition; Approved Standard, 1991.

Nur Für Die In-vitro-Diagnostik

CE

Hergestellt von:

Spectral Medical Inc.
135 The West Mall
Toronto, ON, Kanada
M9C 1C2
+ 1.416.626.3233
FAX: + 1.416.626.7383
www.spectraldx.com

Bevollmächtigter in Europa:

Dr. Rolf Keck
Ritterstraße 25
D-77977 Rust/Baden
Deutschland
+ 49.782.286.5111
FAX: + 49.782.286.5113

Endotoxin Activity Assay (EAA™) ist eine eingetragene Handelsmarke der Spectral Medical Inc.

DE

Solo Per Uso Diagntostico In Vitro

1. Destinazione d'uso

EAA™ e´ un test rapido, di diagnostica in vitro, che utilizza un anticorpo monoclonale specifico per misurare l'attività dell'endotossina in un campione di sangue intero in provetta EDTA.

Il risultato, Se usato in associazione a colture microbiche e altri test diagnostici pertinenti (come ecografia, broncoscopia o TAC) ed altre formazioni cliniche (sintomi, segni vitali, risultati radiografici) può:

- Supportare la stratificazione del rischio di sviluppare una sepsi per un paziente.
- Guidare l'utilizzo di terapie specifiche anti-endotossina

2. Endotoxin Activity Assay - Introduzione

Il test EAA misura il livello dell'attività endotossinica in un campione di sangue prelevato da un paziente specifico in maniera semi quantitativa, distinguendo in 3 gruppi di alta, media e bassa attività endotossinica.

L'endotossina o lipopolisaccaride (LPS) è un importante componente della parete cellulare dei batteri Gram negativi responsabile della sepsi severa[1-2]. I livelli elevati di endotossina possono causare cambiamenti nell'espressione di più di 300 geni e macrofagi attivati, neutrofili, cellule endoteliali e dare origine alla cascata coagulativa che produce la cascata settica[3].

Questo dosaggio sfrutta la risposta biologica dei neutrofili nel sangue del paziente a un complesso immunologico di endotossina e anticorpo esogeno come misura dell'attività endotossinica nel paziente[4]. Il test reagisce in maniera specifica con il Lipide A, componente comune dell'LPS dei batteri Gram negative senza interagire con component della membrana cellulare dei batteri Gram positivi o di altri microorganismi.

3. Contenuto

3.1 EAA™ Kit

Nome	Quantità
EAAST-20	1
EAA™ confezione singola	20
EAA™ Controllo Qualitá confezione singola	1
EAA™ Soluzione fiale	21
Istruzioni per l'uso	1

3.2 EAA™ - Confezione Singola

Nome	Quantità	Descrizione
Tubo 1	2	Stabilizzanti e luminol-zymosan
Tubo 2	2	Monoclonale murino stabilizzatori di anticorpi contro endotossine e luminol-zymosan
Tubo 3	2	Monoclonale murino stabilizzatori di anticorpi contro endotossine e luminol-zymosan
LPS Max Tubo	1	Endotossine (E. coli O55:B5) con stabilizzatori
Aliquota Tubo	1	Tubo vuoto

IT

3.3 EAA™ Controllo Qualitá Confezione Singola

Nome	Quantità	Descrizione
Tubo 1	2	Stabilizzanti e luminol-zymosan
Low Controllo Tubo	1	Monoclonale murino stabilizzatori di anticorpi contro endotossine e luminol-zymosan
Alta Controllo Tubo	1	Monoclonale murino stabilizzatori di anticorpi contro endotossine e luminol-zymosan
Tubo 3	2	Monoclonale murino stabilizzatori di anticorpi contro endotossine e luminol-zymosan
QC LPS Max Tubo	1	Endotossine (E. coli O55:B5) con stabilizzatori
QC Aliquota Tubo	1	Tubo vuoto

3.4 EAA™ Soluzione

Nome	Quantità	Descrizione
EAA™ Soluzione	8 mL/fiale	Soluzione HBSS ed eparina

3.5 Accessori

Non forniti assieme al kit.

- Pipetta a volume variabile (combinata) – in grado di erogare volumi di 40 µl e 1000 µl e una
- micropipetta in grado di erogare un volume di 500 µl
- Puntali sterili per pipetta combinata e micropipetta delivering 40uL e 1000uL
- Provette per prelievi di sangue in EDTA
- Timer
- Luminometro (Berthold Smartline TL Tube or equivalent)
- Incubating shaker capace di incubare campioni a 37°C ed agitarli a 100 rpm
- Vortexer

IT

4. Precauzioni

- Utilizarre cura e attenzioni tipiche da laboratorio nel maneggiare e conservare I Componenti del test confezionati.
- Il test EAA™ utilizza sferette di reagent liofilizzati contenuti in provette di vetro borosilicato. In alcune situazioni (confezioni danneggiate, non perfettamente chiuse, o a seguito di mantenimento in magazzini inadeguati) le sfere possono deformarsi a causa dell' umidità. Non usare il test se le sfere di reafente appaiono deformate.
- In caso di pipettatura manuale di campiono e controlli, usare puntali di pipetta separati per eliminare le contaminazioni reciproche.
- Non usare dopo la data di scadenza specificata sull'etichetta.
- Ultillizzare le "recommended universal precautions"[5] nel maneggiare campioni biologici umani e rifuti.
- Maneggiare con cura i campioni residui di EAA™ solution e di sangue umano usando le precedure correnti in materia.
- Non pipettare le soluzioni con la bocca.
- Pulire immediatamente gli spandimenti con una soluzione allo 0.5% di ipoclorito di sodio.

5. Principio di Funzionamento

Una misurazione dell'attività basale (provetta 1) in assenza dello specifico anticorpo anti-endotossina misura il burst ossidativo non specifico dei neutrofili del paziente. Una misurazione di controllo supplementare includente lo specifico anticorpo anti-endotossina e un eccesso di endotossina esogena (provetta 3) misura il burst ossidativo massimo dei neutrofili del paziente. La misurazione di prova (provetta 2) include l'anticorpo specifico per misurare il livello esatto non diluito di attività endotossinica. Il risultato EAA viene calcolato normalizzando la chemi-luminescenza nel campione del test (provetta 2) rispetto alla chemi-luminescenza massima (provetta 3), correggendo entrambe le misure per la chemi-luminescenza ad attività basale (provetta 1).

IT

5.1 Raccolta del Campione e Preparazione

Utilizzare solamente provette EDTA anti-coagulanti per raccolta di sangue intero. Seguire le direttive raccomandate dallo NCCLS[6] per il prelievo, il trasporto e il trattamento dei campioni dei pazienti. I campioni devono essere raccolti in provette per prelievi di sangue contenenti anticoagulante (EDTA). If sampling through an indwelling arterial or venous cannula or catheter, please flush the lines per your facility's policy prior to collecting blood in the EDTA anti-coagulated tube. I campioni di sangue possono essere conservati per un massimo di 180 minuti a una temperatura ambiente tra 18°C e 25°C prima dell'analisi. Prima dell'analisi, il sangue deve essere miscelato accuratamente per almeno 20-30 secondi capovolgendo la provetta senza agitare meccanicamente.

IT

Si suggerisce di prelevare una quantita minima di 1.5 mL di sangue del paziente per eseguire il test (essendo 1.0 mL in minimo teorico richiesto). Si suggerisce di prelevare una quantita minima di 1.5 mL di sangue del paziente per eseguire il QC test (essendo 1.0 mL in minimo teorico richiesto). Non e' necessario che il sangue usato per il QC test provvenga dallo stesso paziente.

5.2 Istruzioni per L'uso del Test sul Luminometro Berthold Smartline TL

NOTA: Prima di eseguire I test EAA™ o EAA™ QC Test, assicurarsi che il luminometro Berthold SmartLine sia acceso "ON", e che l'heating incubator shaker sia acceso e abbia raggiunto la temperatura di lavoro che deve essere settata a 37°C ± 1.0°C.

5.2.1 EAA™ Procedura di Prova

La buona pratica di laboratorio (GLP) suggerisce che l' EAA™ sia eseguito in duplicato per ciascun paziente. La media dei duplicati rappresenta il risultato EAA™ . Ogni laboratorio deve sviluppare e applicare CV accettabili per l'accettazione delle repliche. È possibile eseguire fino a 3 campioni dii pazienti in un singolo run.

1. Preparare le provette EAA™ per ogni campione di paziente nel portaprovette in dotazione e rimuovere i tappi.
2. Pipettare 1.0 mL della soluzione EAA™ nelle provette 1, 2, e 3 (in duplicato).

3. Dopo aver miscelato il campione di sangue capovolgendo senza agitare, pipettare 0.5 mL di aliquota direttamente nella provetta aliquota e nella provetta LPS MAX. Agitare la provetta LPS MAX.
4. Mettere i protaprovette nell'agitatore incubatore, chiudere il coperchip e incubare per 10 minuti.
5. Trascorsi i 10 minuti d'incubazione, aprire il coperchio, agitare la provetta aliquota poi pipettare 40µL di sangue nelle provetta 1 e 2 (in duplicato) usando un puntale sterile per pipetta.
6. Agitare la provetta LPS Max, poi pipettare 40 µL di sangue nella provetta 3 (in duplicato) usando un puntale sterile per pipetta.
7. Agitare le provette 1, 2 e 3 e rimetterle nei portaprovette nell'agitatore incubatore, chiudere il coperchio e avviare il moto (impostato a 100 rpm) e il timer (impostato a 14 minuti).
8. Inserire la scheda con chip etichettata EAA nel luminometro e premere Avvio.
9. Trascorsi i 14 minuti d'incubazione, seguire le istruzioni sul display del luminometro per leggere le provette EAA™ nel giusto ordine.
10. Agitare delicatamente ogni provetta, aprire il cassetto dei campioni e inserire la provetta nel portacampioni, chiudere il cassetto e attendere il risultato RLU. Ripetere per la provetta successiva.
11. Una volta lette tutte le provette, i risultati EAA™ vengono stampati automaticamente dal dispositivo di stampa integrato.
12. Ripetere per ogni campione paziente, se applicabile.

IT

5.2.1.1 Interpretazione dei Risultati

Gamma (valori EAA™)	Interpretazione dei risultati
0.0 a 0.39	un livello basso di attività endotossinica rappresenta un rischio basso di sepsi severa.
0.40 a 0.59	un livello intermedio di attività endotossinica rappresenta un rischio elevato di sepsi severa.
≥ 0.60	un livello elevato di attività endotossinica rappresenta un rischio elevato di sviluppo di sepsi severa.

5.2.2 Procedura per EAA™ QC Test (controllo qualita')

Si raccomanda di eseguire un QC test per ogni confezione di EAA™. Ogni laboratorio può elaborare tuttavia un proprio regime di controlli secondo gli standard locali.

1. Inserire le provette QC in uno dei portaprovette in dotazione e togliere i tappi.
2. Pipettare 1.0 mL di "EAA™ Soultion" in ogni provetta.
3. Pipettare 0.5 mL di aliquote di sangue intero direttamente nella provetta "QC aliquote" e nella provetta QC LPS MAX. Agitare la provetta QC LPS MAX.
4. Mettere il portaprovette nell'agitatore incubatore, chiudere il coperchio e incubare per 10 minuti.
5. Trascorsi i 10 minuti d'incubazione, aprire il coperchio, agitare la provetta CQ aliquota poi pipettare 40 μL di sangue sia nella provetta 1 che nella provetta controllo basso usando un puntale sterile per pipetta.
6. Agitare la provetta QC LPS MAX, poi pipettare 40 μL di sangue prima nella provetta controllo alto e poi nella provetta 3 usando un puntale sterile per pipetta.
7. Agitare tutte le provette e rimetterle nei portaprovette nell'agitatore incubatore, poi avviare il moto (impostato a 100 rpm) e il timer (impostato a 14 minuti).
8. Inserire la scheda con chip etichettata EAA QC nel luminometro e premere Avvio.
9. Trascorsi i 14 minuti d'incubazione, seguire le istruzioni sul display del luminometro per leggere le provette EAA™ QC nel giusto ordine.
10. Agitare delicatamente ogni provetta, aprire il cassetto dei campioni e inserire la provetta nel portacampioni, chiudere il cassetto e attendere il risultato RLU. Ripetere per la provetta successiva.
11. Una volta lette tutte le provette, i risultati del QC EAA™ vengono stampati automaticamente dal dispositivo di stampa integrato.

5.2.2.1 Interpretazione dei Risultati

La EAA™ Solution QC funziona correttamente se si ottengono I seguenti valori:

Interpretazione dei Risultati	EAA™ Risultato
EAA QC basso	0.00 – 0,20
EAA QC alto	0.80 – 1.50

Se i risultati del test di QC per controllo alto o basso non danno i risultati desiderati, ripetere il test. Se anche ripetendo il test di QC i risultati rimangono al di fuori dei limiti accettabili, i risultati successivi dei test EAA™ potrebbero non essere validi. Segnalare il fatto al vostro rappresentante EAA™ al più presto.

5.3 Limiti

5.3.1 Analitici

- Come per tutti gli immuno-dosaggi o le reazioni immunochimiche, si richiede una tecnica accurata.
- L'utilizzatore deve prestare attenzione per i possibili effetti sui risultati delle interferenze potenziali dovute a farmaci o sostanze endogene sconosciute. Vedere Sostanze interferenti più avanti.

5.3.2 Clinici

- I risultati dell'EAA™ dovrebbero essere valutati nel contesto di tutti gli esami di laboratorio e dello stato clinico complessivo del paziente. Nei casi in cui i risultati di laboratorio non concordano con il quadro clinico o l'anamnesi, si dovranno eseguire esami ulteriori.
- Alcuni studi pubblicati in precedenza hanno riferito riscontri di risultati positivi per l'endotossina in pazienti senza sepsi severa.
- I risultati di Test EAA ottenuti su pazienti non ricoverati in terapia intensiva vanno interpretati con cautela visto che non ne è mai stato valutato il significato clinico.

5.4 Performance Clinica

Il trial “Rilevamento multicentrico dell’endotossina nelle patologie critiche” (MEDIC) è uno studio d’osservazione multicentrico e prospettico svolto in 10 unità di cura intensiva (ICU) in ambiente clinico universitario in America del nord e in Europa. La presenza di endotossinemia è stata valutata il primo giorno di ricovero in ICU dei pazienti per determinare la probabilità di sviluppo di una sepsi severa entro 24 ore dall’ammissione all’ICU. La popolazione target per lo studio di valutazione del rischio includeva tutti i pazienti eleggibili arruolati nel trial MEDIC al primo giorno di ammissione all’ICU che avevano campioni valutabili, N=857. La demografia dei pazienti è riportata nella tabella seguente.

IT

Demografia e caratteristiche di baseline dei pazienti analizzati

Caratteristica	
Età: media (SD) mediana [IQR]* anni	60 (±17) 62 [49, 74]
Genere (% maschi)	58.9%
Razza (% non caucasici)	15.8%
APACHEII: media (SD) mediana [IQR]*	15.2 (±9,5) 14 [8, 21]
Giorni in ICU: media (SD)	5.4 (±10,9) 2.0 [1, 5]
Giorni in ospedale: media (SD)	23.3 (±27,0) 14.0 [7, 29]
Mortalità ICU	13.3%
Mortalità ospedale	19.9%

L’utilità dei test EAA™ è stata accertata solo per la popolazione in cura critica. Il rapporto tra i livelli di endotossina e il rischio di sviluppo di sepsi severe nei pazienti con patologie critiche è inteso come supplemento alle informazioni cliniche disponibili correntemente per stabilire il rischio di sepsi severa nella prima giornata di ricovero in ICU.

5.5 Caratteristiche della Performance

5.5.1 Sostanze Interferenti

- I livelli di trigliceridi fino a 1000 mg/dL non interferiscono con le misurazioni EAA™. viceversa e’ stato dimostrato che concentrazioni maggiori di 1500 mg/dL tendono a ridurre il valore EAA. Si dovrebbe evitare l’uso di campioni fortemente lipemici nell’EAA™.
- Emoglobina libera fino a concentrazioni di 100 mg/dL non interferisce con I risultati EAA™ mentre a livelli molto alti come 500 mg/dL tende ad attenuare significativamente il segnale su pazienti con bassa attivita’ endotossinica EAA < 0.3 e ridurre in generale di circa 15% i valori alti di EAA. Si dovrebbe evitare l’uso di campioni fortemente emolizzati nel test EAA™.
- L’infusione esogena di albumina esente da endotossina nei pazienti a livelli che aumentano l’albumina nel plasma di oltre il 10 g/L al di sopra del limite superiore del normale può abbassare i valori EAA. I campioni da pazienti con anomalie iper-proteiche o che ricevono terapie albuminiche devono essere sottoposti a determinazioni proteiche per accertare che gli elevati livelli di albumina non siano una fonte di interferenze con l’EAA™.

IT

5.5.2 Precisione

Campioni di sangue intero, non diluiti e con aggiunta di endotossina esogena, sono stati analizzati in otto replicati su ciascuno di tre strumenti. È stata calcolata la precisione sia durante l’esecuzione che totale secondo le procedure NCCLS in EP5-A.

Campione	N	Medio EAA™	Entro	Gestire	Totale Precisione*	
			1SD	%CV	1SD	%CV
1	24	0.11	0.015	14	0.023	22
2	24	0.20	0.030	15	0.029	14
3	24	0.30	0.043	15	0.042	14
4	24	0.50	0.059	12	0.064	13
5	24	0.52	0.036	7	0.034	6
6	24	0.59	0.050	8	0.046	8

5.5.3 Sensibilità Analitica

La sensibilita del test e' stata valutata usando campioni di sangue a bassa attivita' endotossinica. Sono state eseguite ventiquattro determinazioni EAA™ su un singolo campione paziente con basso livello EAA™; otto misurazioni su ciascuno di tre strumenti. Il livello EAA™ medio del paziente era di 0.11 con una SD di 0.023. La Sensitivita' del test, usando il metodo delle 2 deviazioni standard e' di 0.046 EAA units.

5.5.4 Specificità Analitica

Endotossina proveniente da batteri diversi dal E. coli O55:B5, come P. aeruginosa, K. pneumoniae, S. enterides, E. coli 0127:B8, S. marcescens, e S. flexnevi inducono aumenti dei valori EAA™ da 0.10 a 0.52 quando 100 pg/mL dell'endotossina specifica vengono aggiunti ad un campione di sangue che abbia una attivita' endotossinica originale di EAA = 0.33.L'aggiunta di endotossina di V. cholerae, inoltre, genera un aumento statisticamente significativo dei livelli EAA. Non si è vista alcuna reattività fino a 2000 pg/mL di estratti dell'acido lipoteicoico dai seguenti ceppi di batteri Gram positivi: S. mutans, S. pyogenes, S. sanguis, S. faecalis, S. aureus e B. subtilis. Analogamente, non si è riscontrata alcuna reattività del mannano del lievito fino a 2000 pg/mL e degli estratti di parete cellulare di C. albicans e A. fumagatis.

5.5.5 Linearità dei Risultati

Il dosaggio è stato ottimizzato per essere altamente sensibile a bassi livelli di endotossina e per resistere all'effetto gancio ad alte dosi indotto da livelli di endotossina evidentemente elevati. Pertanto la curva ha andamento curvilineo. Piccolo aumenti della concentrazione di endotossina producono incrementi di EAA maggiori su campioni a bassa concentrazione che su campioni ad alta concentrazione. Non e' necessario diluire i campioni per misurare alti livelli di EAA.

6. Stabilita' e Conservazione delle Confezioni

Il kit EAA™ ed I suoi componenti devono essere conservati (sigillati nel package originale) tra 2-25°C, e non devono essere utilizzati oltre la data di scadenza.

IT

7. Bibliografia:

1. Van Deventer SJH, et al. Endotoxemia: an early predictor of septicemia in febrile patients. The Lancet (March 19) 1988; 605-609.
2. Parrillo JE, Parker MM, Natanson C, et al. Septic shock in humans: advances in the understanding of pathogenesis, cardiovascular dysfunction, and therapy. Ann Int Med 1990; 113: 227-242.
3. Zhao B, Bowden RAS, Stavchansky SA, et al. Human endothelial cell response to gram-negative lipopolysaccharide assessed with cDNA microarrays. Am J Cell Physiol 2001; 281: C1587-C1595.
4. Romaschin AD, et al. A rapid assay of endotoxin in whole blood using autologous neutrophil dependent chemiluminescence. J. of Immunology. Methods 1998; 212: 169-185.
5. NCCLS (National Committee for Clinical Laboratory Standards, 711 East Lancaster Avenue, Villanova, PA 19085) Document # M29-A Protection of Laboratory Worker from Instrument Biohazards and Infectious Disease Transmitted by Blood, Body Fluids and Tissues; Approved Guideline, 1997.
6. NCCLS (National Committee for Clinical Laboratory Standards, 711 East Lancaster Avenue, Villanova, PA 19085) Document # H3-#a Procedures for the Collection of Diagnostic Blood Specimens by Venipuncture – Third Edition; Approved Standard, 1991.

Solo Per Uso Diagntostico In Vitro

Fabbricato Da:

Spectral Medical Inc.
135 The West Mall
Toronto, ON, Canada M9C 1C2
+ 1.416.626.3233
FAX: + 1.416.626.7383
www.spectraldx.com

Rappresentante autorizzato per l'Europa:

Dr. Rolf Keck
Ritterstraße 25
D-77977 Rust/Baden, Germany
+ 49.782.286.5111
FAX: + 49.782.286.5113

Endotossina Activity Assay (EAA™) è un marchio registrato di Spectral Medical Inc.

Только для диагностики In Vitro

1. Применение

Анализ активности эндотоксина (EAA™) – это лабораторный экспресс-анализ цельной крови, который использует определенные моноклональные антитела, для измерения активности эндотоксина в пробах цельной крови с EDTA (ЭДТА).

Эта информация при использовании в сочетании с посевом на микрофлору и другими диагностическими тестами (ультразвуковое исследование, бронхоскопия или компьютерная томография) и клинические данные (клинические симптомы и/или результаты рентгенографического исследования) могут:

- помочь в оценке риска развития тяжелого сепсиса у пациентов;
- определить лечение для нейтрализации действия эндотоксина

2. Анализ активности эндотоксина – Введение

Анализ активности эндотоксина (EAA) проводится для оценки уровня активности эндотоксина в образце крови конкретного пациента полуколичественным методом; выделяется 3 группы активности эндотоксина – низкая, средняя и высокая).

Эндотоксин или липополисахарид А (ЛПС) является облигатным компонентом наружной клеточной мембраны грамотрицательных бактерий и первичным продуктом грамотрицательных бактерий, ответственным за септический шок[1-2]. Высокий уровень эндотоксина в крови вызывает изменения в экспрессии более 300 генов, активизацию макрофагов, нейтрофилов, эндотелиальных клеток, вызывает активацию системы свёртывания крови, приводящие, в итоге, к развитию септического процесса[3].

Этот анализ использует биологическую реакцию нейтрофилов в крови пациента на иммунологический комплекс эндотоксина и экзогенных антител как меру активности эндотоксина у пациента[4]. Реагент для анализа вступает в реакцию исключительно с молекулой липида А ЛПС грамотрицательных бактерий, но не взаимодействует с элементами клеточной мембраны грамположительных бактерий и иных микроорганизмов.

3. Содержание

3.1 Комплект для EAA™

Название	Кол-во
EAAST-20	1
Лотки EAA™	20
Лотки для контроля качества EAA™	1
Флаконы с раствором для EAA™	21
Инструкции по использованию	1

3.2 Лоток для EAA™

Название	Кол-во	Описание
Пробирка 1	2	Стабилизаторы и люминол-зимозан
Пробирка 2	2	Мышиные моноклональные антиэндотоксичные антитела, стабилизаторы и люминол-зимозан
Пробирка 3	2	Мышиные моноклональные антиэндотоксичные антитела, стабилизаторы и люминол-зимозан
Пробирка с избытком ЛПС	1	Эндотоксин (E. coli O55:B5) со стабилизаторами
Пробирка для образца	1	Пустая пробирка

RU

3.3 Лоток для контроля качества EAA™

Название	Кол-во	Описание
Пробирка 1	2	Стабилизаторы и люминол-зимозан
Пробирка с низким уровнем	1	Мышиные моноклональные антитела, стабилизаторы и люминол-зимозан
Пробирка с высоким уровнем	1	Мышиные моноклональные антиэндотоксичные антитела, стабилизаторы и люминол-зимозан
Пробирка 3	2	Мышиные моноклональные антиэндотоксичные антитела, стабилизаторы и люминол-зимозан
Пробирка с избытком ЛПС для контроля качества	1	Эндотоксин (E. coli “055:B5”) со стабилизаторами
Пробирка для образца для контроля качества	1	Пустая пробирка

3.4 Раствор для EAA™

Название	Кол-во	Описание
Раствор EAA™	8 мл/флакон	Сбалансированный солевой раствор Хенкса и гепариновый раствор

3.5 Принадлежности

Не входят в комплект.

- Комбинированный пипеточный дозатор
- Микропипетка 500 мкл
- Стерильные носики для комбипипеток емкостью 40 мкл и 1000 мкл
- Носики для микропипеток емкостью 1000 мкл
- Пробирки для сбора крови с ЭДТА
- Таймер
- Люминометр (Berthold Smartline TL Tube или эквивалентный)
- Инкубационный шейкер, выполняющий инкубацию образцов при температуре 37°C со скоростью перемешивания 100 об/мин
- Вортексный миксер

4. Меры предосторожности

- Используйте общие лабораторные меры предосторожности при работе и хранении запечатанных материалов для анализа.
- EAA™ содержит лиофилизированные гранулированные реагенты в боросиликатных стеклянных пробирках. При определенных условиях (при повреждении упаковки, неправильной герметизации мешочков или неправильном хранении) гранулы могут сжиматься из-за избытка влажности. Не используйте пробирки, содержащие сжатые гранулы, поскольку результаты анализа могут быть недостоверными.
- При ручном пипетировании проб и контролей используйте индивидуальные носики во избежание контаминации и появления осадка.
- Не используйте материалы после истечения срока годности, указанного на этикетке.
- Используйте рекомендуемые универсальные меры предосторожности[5] при обращении с использованными растворами и человеческими образцами.
- Обращайтесь с использованными растворами EAA™ и всеми препаратами человеческой крови и утилизируйте их, соблюдая универсальные меры предосторожности.
- Не пипетируйте растворы ртом.
- Пролитые реагенты следует как можно быстрее промыть 0,5% раствором гипохлорида.

5. Принцип

Измерение базальной активности (Tube 1 (Пробирка 1)) в отсутствие специфического антиэндотоксичного антитела определяет неспецифический оксидативный всплеск нейтрофилов больного. Дополнительное контрольное измерение, включая специфические

антиэндотоксичные антитела и избыток экзогенного эндотоксина (Tube 3 (Пробирка 3)), определяет максимальный оксидативный всплеск нейтрофилов больного. Аналитическое измерение (Tube 2 (Пробирка 2)) включает специфические антитела для определения фонового уровня активности эндотоксина. Результат EAA рассчитывается путем нормализации хемилюминесценции в тестируемом образце (Tube 2 (Пробирка 2)) относительно максимальной хемилюминесценции (Tube 3 (Пробирка 3)) с коррекцией обоих результатов по базальной активности хемилюминесцениии (Tube 1 (Пробирка 1)).

5.1 Подготовка и проведение анализа

Для сбора образцов цельной крови для этой процедуры используйте только пробирки для забора крови с антикоагулянтом ЭДТА. При сборе, транспортировке и обработке проб крови должны соблюдаться Указания NCCLS[6] (или иного регламентирующего органа, действующего в вашей стране). Образцы крови должны собираться в пробирки с антикоагулянтом ЭДТА венопункцией или через постоянный катетер. Если пробы отбираются через постоянную артериальную или венозную канюлю или катетер, промойте систему в соответствии с правилами вашего учреждения перед забором крови в пробирку с антикоагулянтом ЭДТА. Пробы крови перед анализом могут храниться в течение 180 минут при комнатной температуре (18-25 градусов Цельсия). Перед проведением анализа проба должна быть тщательно перемешана в течение 20-30 секунд аккуратными вращательными движениями.

Для выполнения анализа рекомендуется взять минимум 1,5 мл цельной крови пациента (требуется 1,0 мл). Для выполнения анализа для контроля качества рекомендуется взять минимум 1,5 мл цельной крови (требуется 1,0 мл). Для контроля качества анализа может быть взят отдельный образец цельной крови.

5.2 Инструкции по использованию люминометра Berthold Smartline TL Tube

Примечание: Перед началом анализа EAA™ или EAA™ QC убедитесь, что люминометр Berthold SmartLine TL Tube включен, и нагревательный шейкер-инкубатор включен и разогрет до 37°C ± 1,0°C.

RU

5.2.1 Процедура анализа EAA™

Согласно передовой практике лабораторных исследований EAA проводится дважды для каждого пациента. Среднее значение двух анализов используется для отчетности о результатах EAA™. Каждая лаборатория определить приемлемый уровень допустимого отклонения для повторных анализов. Вы можете проводить исследование до 3-х проб от разных пациентов одновременно.

1. Установите пробирки EAA™ с анализами для каждого пациента в штатив для пробирок и снимите крышки.
2. Используя комбипипетку 1,0 мл, поместите раствор EAA™ в пробирки 1, 2 и 3 (в двух экземплярах).
3. Осторожно перемешайте образец крови пациента, плавно покачивая пробирку, введите 0,5 мл образца непосредственно в пробирку для образца (Aliquote Tube) и в пробирку с избытком ЛПС (LPS MAX Tube). Перемешайте содержимое пробирки с избытком ЛПС (LPS MAX Tube) на вихревой мешалке.
4. Поместите штатив в термостат-шейкер, закройте крышку и инкубируйте в течение 10 минут.
5. После 10-минутной инкубации откройте крышку, перемешайте содержимое пробирки с образцом (Aliquote Tube) на вихревой мешалке и, используя стерильный наконечник комбинированной пипетки, введите 40 мкл крови в пробирки 1 и 2 (Tube 1 и Tube 2) и в их дубликаты.
6. Перемешайте содержимое пробирки с избытком ЛПС (LPS MAX Tube) на вихревой мешалке, затем, используя тот же наконечник комбинированной пипетки, введите 40 мкл крови в пробирку 3 (Tube 3) и в ее дубликат.
7. Перемешайте на вихревой мешалке пробирки 1, 2 и 3 (Tube 1, Tube 2 и Tube 3) и поместите их обратно в штатив, затем поставьте штатив в термостат-шейкер, закройте крышку, запустите мешалку (на 100 об/мин) и таймер (установленный на 14 минут).
8. Вставьте маркированную чип-карту EAA™ в люминометр и нажмите Пуск.

RU

9. После 14-минутной инкубации выполните инструкции, отображаемые на экране люминометра для того, чтобы получить данные для пробирок EAA в надлежащем порядке.
10. Осторожно перемешайте на вихревой мешалке каждую пробирку, откройте отсек для образца и поместите пробирку 1 (Tube 1) в держатель для образца, закройте отсек для образца, подождите пока считываются показания в единицах RLU. Повторите те же действия со следующими пробирками в порядке, указываемом на дисплее люминометра.
11. После считывания показаний со всех пробирок результаты EAA распечатываются автоматически на выводном печатающем устройстве.
12. При необходимости повторите процедуру для анализа крови другого пациента.

RU

5.2.1.1 Интерпретация результатов

Ассортимент (EAA™ результат)	Интерпретация результатов
0,0 - 0,39	Низкий уровень эндотоксина означает низкий риск развития тяжелого сепсиса.
0,40 - 0,59	средний уровень активности эндотоксина означает повышенный риск развития тяжелого сепсиса.
≥ 0,60	высокий уровень активности эндотоксина означает высокий риск развития тяжелого сепсиса.

5.2.2 Процедура анализа качества EAA™

Рекомендуется проводить один анализ качества на коробку для EAA™. Однако каждая лаборатория может разработать свой собственный режим тестирования QC согласно местным стандартам.

1. Установите пробирки QC в штатив для пробирок и снимите крышки.
2. Используя комбипипетку 1,0 мл, поместите раствор EAA™ в каждую пробирку.

3. Введите 0,5 мл образца крови непосредственно в пробирку для образца (QC Aliquot Tube) в пробирку с избытком ЛПС для теста контроля качества (QC LPS MAX Tube). Перемешайте содержимое пробирки с избытком ЛПС для теста контроля качества (QC LPS MAX Tube) на вихревой мешалке.
4. Поместите штатив в термостат-шейкер, закройте крышку и инкубируйте в течение 10 минут.
5. После 10-минутной инкубации откройте крышку, перемешайте содержимое пробирки для образца (QC Aliquot Tube) на вихревой мешалке и, используя стерильный наконечник комбинированной пипетки, введите 40 мкл крови в обе пробирки 1 (Tube 1) и контрольную пробирку с низким уровнем (Low Control Tube).
6. Перемешайте содержимое пробирки с избытком ЛПС для теста контроля качества (QC LPS MAX Tube) на вихревой мешалке, затем, используя стерильный наконечник комбинированной пипетки, введите 40 мкл крови в контрольную пробирку с высоким уровнем (High Control Tube), а затем в обе пробирки 3 (Tube 3).
7. Перемешайте на вихревой мешалке все пробирки и поместите их обратно в штатив, затем поставьте штатив в термостат-шейкер, запустите мешалку (на 100 об/мин) и таймер (установленный на 14 минут).
8. Вставьте маркированную чип-карту EAA™ QC в люминометр и нажмите Пуск.
9. После 14-минутной инкубации выполните инструкции, отображаемые на экране люминометра для того, чтобы получить данные для пробирок EAA QC в надлежащем порядке.
10. Осторожно перемешайте на вихревой мешалке каждую пробирку, откройте отсек для образца и поместите пробирку в держатель для образца, закройте отсек для образца, подождите пока считываются показания в единицах RLU. Повторите те же действия с другой пробиркой.
11. После считывания показаний со всех пробирок результаты EAA™ QC распечатываются автоматически .

RU

5.2.2.1 Интерпретация результатов

Качество раствора EAA™ QC подтверждается следующими результатами:

Интерпретация результатов	Результат раствора для EAA™
Низкий EAA QC	0,00 – 0,20
Высокий EAA QC	0,80 – 1,50

Если результаты QC теста для Low (низкого) и High (высокого) контрольных уровней не соответствуют ожидаемым результатам, то тест необходимо повторить. Если повторный QC тест также даст результаты за пределами ожидаемых величин, то данные анализа EAA™ должны быть подвергнуты сомнению. Пожалуйста, немедленно сообщите об этом производителю или его представителю.

5.3 Ограничения

5.3.1 Аналитические

- Необходима точность выполнения протоколов работы со всеми иммунологическими наборами и иммунохимическими реакциями.
- Пользователь должен быть внимателен, поскольку на результаты могут влиять как медицинские препараты, так и неизвестные эндогенные вещества. Влияние веществ рассматривается далее.

5.3.2 Клинические

- Результаты EAA™ следует рассматривать в контексте все лабораторных исследований и общего клинического состояния пациента. В случае, если лабораторные данные не соответствуют клинической картине или истории заболевания, следует выполнить дополнительное тестирование.
- Выявление позитивных результатов активности эндотоксина при отсутствии тяжелого сепсиса отмечались ранее в опубликованных материалах.
- Результаты , полученные на пациентов вне интенсивной терапии следует интерпретировать с осторожностью, так как они не могут иметь существенное клиническое значение .

5.4 Клиническая эффективность

Совместно с 10 институтами Европы и Северной Америки с отделениями интенсивной терапии было проведено мульти-центровое исследование по определению активности эндотоксина при тяжелых состояниях (MEDIC). Наличие эндотоксемии было оценено на первый день нахождения пациентов в ОРИТ для определения риска развития тяжелого сепсиса в первые 24 часа в условиях интенсивной терапии. Целевая группа для исследования по оценке риска включала всех возможных пациентов, вовлеченных в MEDIC на первые сутки после направления в палату интенсивной терапии с поддающимися оценке значениями, N=857. Демография пациентов представлена в следующей таблице.

Демографические и основные особенности обследуемых пациентов.

Характеристика	
Возраст: средняя медиана (SD)	60 (±17) 62 [49, 74]
Пол (% мужской)	58,9%
Раса (% не белый)	15,8%
APACHE II: среднее значение (SD)	15,2 (±9,5) 14 [8, 21]
Дней в палате интенсивной терапии: среднее (SD)	5,4 (±10,9) 2,0 [1, 5]
Дней в госпитале: среднее (SD)	23,3 (±27,0) 14,0 [7, 29]
Смертность в отделении интенсивной терапии	13,3%
Смертность в госпитале	19,9%

Тестирование EAA™ лучше всего проводить у людей, находящихся в критическом состоянии. Именно для пациентов в критическом состоянии был создан метод тестирования EAA™. Соотношение между уровнем эндотоксина и риском развития тяжелого сепсиса у пациентов в критическом состоянии помогает дополнить имеющуюся клиническую информацию для определения риска развития тяжелого сепсиса на первый день поступления в палату интенсивной терапии.

RU

5.5 Качественные показатели

5.5.1 Взаимодействие веществ

- Триглицериды при уровне вплоть до 1000 мг/дЛ не влияют на измерения EAA. At При концентрациях триглицерида бьлее 1500 мг/дл наблюдалось снижение результатов EAA. При измерении EAA™ не следует использовать пробы крови с высоким содержанием жира.
- Гемоглобин в дозе 100 мг/дл не влияет на показатели EAA, но при 500 мг/дл снижаются показатели низкого уровня EAA™ менее чем на 0,03 результата EAA и снижаются повышенные показатели EAA в среднем на 15%. Не используйте макроскопически гемолизированные образцы для EAA™.
- Экзогенное введение пациентам не содержащего эндотоксин альбумина в количестве, повышающем уровень альбумина в плазме более чем на 10 г/л относительно верхней нормы, может снижать уровень EAA. При заборе крови у пациентов с высокобелковыми аномалиями или получающими альбуминовую терапию следует проводить процедуры удаления белков, для уверенности в том, что уровень удаленного альбумина не является источником искажения значений EAA™.

5.5.2 Точность

Были проанализированы пробы цельной крови, без примесей и пробы с добавленным эндотоксином на трех приборах в восьми повторах. Согласно процедуре NCCLS в EP5-A были посчитаны общая точность и повторяемость результатов.

Проба	N	Среднее EAA™	В рамках измерения*		Общая точность*	
			1SD	%CV	1SD	%CV
1	24	0,11	0,015	14	0,023	22
2	24	0,20	0,030	15	0,029	14
3	24	0,30	0,043	15	0,042	14

Проба	N	Среднее EAA™	В рамках измерения*		Общая точность*	
4	24	0,50	0,059	12	0,064	13
5	24	0,52	0,036	7	0,034	6
6	24	0,59	0,050	8	0,046	8

5.5.3 Чувствительность

Для оценки чувствительности метода EAA использовался образец пациента с низким показателем EAA. 24 измерения EAA™ были сделаны для одного пациента с низким уровнем EAA™, на 3 приборах по восемь измерений для каждой пробы. Среднее значение уровня EAA™ пациента было 0,11, стандартное отклонение составило 0,023. Таким образом, чувствительность методом 2 стандартных отклонений предполагает результат EAA, равный 0,046.

5.5.4 Специфичность

Эндотоксин из грамотрицательных бактерий, E. coli O55:B5, P. aeruginosa, K. pneumoniae, S. enterides, E. coli 0127:B8 и S. marcescens, и S. flexnevi приводит к увеличению уровней EAA™ с 0,10 до 0,52, когда 100 пг/мл эндотоксина добавляют к пробе крови с результатом EAA 0,33. Кроме того, препарат V. cholerae LPS приводит к статистически достоверному увеличению результатов EAA. Не отмечено влияния экстракта липотейхоевой кислоты вплоть до 2000 пг/мл, полученной от штаммов грамм-позитивных бактерий: S. mutans, S. pyogenes, S. sanguis, S. aureus и B. subtilis. Также не отмечена реакционная способность на маннаны дрожжей вплоть до 2000пг/мл и экстракты клеточной стенки C. Albicans и A. Fumagatis.

5.5.5 Линейность результатов

Реактив был оптимизирован для высокой чувствительности при низком уровне эндотоксина и устойчивости к хук-эффекту при высокой дозе, вызванной значительно повышенным уровнем эндотоксина. Поэтому кривая имеет нелинейную форму. Когда пробы пациентов содержат низкий уровень эндотоксина, его незначительное увеличение

соответствует значительному росту EAA уровня. Для проб с высоким уровнем EAA не требуется разведение.

6. Хранение и стабильность

Анализ активности эндотоксина (EAA™) является зарегистрированной торговой маркой компании Spectral Medical Inc.

7. Дополнительная литература:

1. Van Deventer SJH, et al. Endotoxemia: an early predictor of septicemia in febrile patients. The Lancet (March 19) 1988; 605-609.
2. Parrillo JE, Parker MM, Natanson C, et al. Septic shock in humans: advances in the understanding of pathogenesis, cardiovascular dysfunction, and therapy. Ann Int Med 1990; 113: 227-242.
3. Zhao B, Bowden RAS, Stavchansky SA, et al. Human endothelial cell response to gram-negative lipopolysaccharide assessed with cDNA microarrays. Am J Cell Physiol 2001; 281: C1587-C1595.
4. Romaschin AD, et al. A rapid assay of endotoxin in whole blood using autologous neutrophil dependent chemiluminescence. J. of Immunology. Methods 1998; 212: 169-185.
5. NCCLS (National Committee for Clinical Laboratory Standards, 711 East Lancaster Avenue, Villanova, PA 19085) Document # M29-A Protection of Laboratory Worker from Instrument Biohazards and Infectious Disease Transmitted by Blood, Body Fluids and Tissues; Approved Guideline, 1997.
6. NCCLS (National Committee for Clinical Laboratory Standards, 711 East Lancaster Avenue, Villanova, PA 19085) Document # H3-#a Procedures for the Collection of Diagnostic Blood Specimens by Venipuncture – Third Edition; Approved Standard, 1991.

RU

Только для диагностики In Vitro

CE

Изготовитель:

Spectral Medical Inc.
135 The West Mall
Toronto, ON, Canada
M9C 1C2
+ 1.416.626.3233
ФАКС: + 1.416.626.7383
www.spectraldx.com

Уполномоченный представитель в Европе:

Dr. Rolf Keck
Ritterstraße 25
D-77977 Rust/Baden
Germany
+ 49.782.286.5111
ФАКС: + 49.782.286.5113

Анализ активности эндотоксина (EAA™) является зарегистрированной торговой маркой компании Spectral Medical Inc.

RU

Endotoksin Aktivite Tahlili (EAA™)

Yalnızca In Vitro Teşhis Kullanımı İçindir

1. Kullanım Amacı

EAA™, özel monoklonal antikordan faydalanarak, EDTA i tüm kan örneğinin içindeki endotoksin aktivitesini ölçebilmek için hızlı bir in vitro teşhis testidir. Mikrobik kültürler ve diğer ilgili teşhis testleri (ultrason, bronkoskopi veya CT Tarama gibi) ile birlikte kullanıldığında (klinik belirtiler ve/veya radyagrafik test sonuçları) testin tavsiye edilen kullanımı:

- Hastaların şiddetli sepsis progresyonunun risk değerlendirmesine yardımcı olur.
- Hastaları spesifik, anti-endotoksin tedavisinden yararlanmaya yönlendirin.

2. Endotoksin Aktivite Testi

EAA tahlili, belirli bir hastanın ait kan numunesindeki endotoksin seviyesini yarı kantitatif şekilde ölçer; bunu yaparken söz konusu aktiviteyi düşük, orta ve yüksek olmak üzere 3 gruba ayırır.

TR

Endotoksin veya Lipopolisakkaritler (LPS), Gram negatif bakterilerin hücre duvarında bulunan önemli bir bileşeni ve birincil Gram negatif bakteriyel ürünü olup ağır sepsisin sorumlusudur.[1-2] Yükselmiş olan endotoksin seviyeleri, 300'den fazla genin ifadesinde değişikliğe ve aktive edilmiş makrofajları, nötrofiller, endotelyal hücreleri ve ardarda koagülasyon ile sepsisin kademeli olarak ilerlemesine neden olur.[3]

Bu tahlil, hastanın kanındaki nötrofillerin endotoksin ve eksojen antikorun immünolojik kompleksine verdiği biyolojik yanıtı hastadaki endotoksin aktivitesinin ölçüsü olarak kullanır.[4] Tahlil, spesifik olarak Gram negatif bakterilerin Lipid A parçasıyla reaksiyona girer ve Gram pozitif bakterilerin hücre duvarı bileşenleriyle ve diğer mikroorganizamalarla çapraz reaksiyona girmez.

3. İçindekiler

3.1 EAA™ Kiti

Adı	Miktarı
EAAST-20	1
EAA™ Tepsileri	20
EAA™ Kalite Kontrol Tepsileri	1
EAA™ Çözelti Flakonları	21
Kullanma Kılavuzu	1

3.2 EAA™ Tepsileri

Adı	Miktarı	Tanımı
Tüp 1	2	Stabilizatörler ve luminol-zymosan
Tüp 2	2	Murin monoklonal anti-endotoksin antikoru, stabilizatörler ve luminol-zymosan
Tüp 3	2	Murin monoklonal anti-endotoksin antikoru, stabilizörler ve luminol-zymosan
LPS Max Tüpü	1	Stabilizatör içeren Endotoksin (E. coli O55:B5)
Sıvı Bölüntü Tüpü	1	Boş tüp

3.3 EAA™ Kalite Kontrol Tepsileri

Adı	Miktarı	Tanımı
Tüp 1	2	Stabilizatörler ve luminol-zymosan
Düşük Kontrol Tüpü	1	Murin monoklonal antikoru, stabilizörler ve luminol-zymosan
Yüksek Kontrol Tüpü	1	Murin monoklonal anti-endotoksin antikoru, stabilizatörler ve luminol-zymosan
Tüp 3	2	Murin monoklonal anti-endotoksin antikoru, stabilizatörler ve luminol-zymosan
QC LPS Max Tüpü	1	Stabilizör içeren Endotoksin (E. coli O55:B5)
QC Sıvı Bölüntü Tüpü	1	Boş tüp

TR

3.4 EAA™ Çözeltisi

Adı	Miktarı	Tanımı
EAA™ Çözeltisi	8 mL/flakon	HBSS ve Heparin Çözeltisi

3.5 Aksesuarlar

Kit ile birlikte temin edilmez.

- Bileşik pipet
- 500 µL Mikropipet
- 40ul ve 1000ul dağıtım kapasitesine sahip steril bileşik pipet uçları
- 1000uL mikropipet uçları
- EDTA kan alma tüpleri
- Zamanlayıcı
- Luminometre (Berthold Smartline veya muadili)
- Numuneleri 37°C'de inkübe etme ve 100 rpm'de çalkalama kapasitesine sahip çalkalayıcı inkübatör
- Vorteksleme cihazı

4. Önlemler

TR

- Açılmamış tahlil materyallerini ellerken ve muhafaza ederken genel laboratuvar tedbirlerini alınız.
- EAA™ borosilikat cam test tüplerinde liyofilize boncuk şeklinde reaktifler içerir. Belirli koşullar altında (hasarlı paket, poşetlerin hatalı kapanması veya uygunsuz depolama) bu boncuklar aşırı nem varlığında küçülebilirler. Hatalı test sonuçlarından kaçınmak için içerdiği boncukları küçülmüş tüpleri kullanmayın.
- Örnekleri manuel pipetle alırken ve kontrolleri için, bir pipetten diğer pipete nakli önlemek için ayrı ayrı pipet uçları kullanın..
- Etiketin üzerinde belirtilen son kullanma tarihi geçmiş ürünleri kullanmayınız.
- Kullanılmış çözeltiler ve insanlardan alınan numuneler için genelde tavsiye edilen önlemleri[5] alınız.
- Kullanılmış EAA™ çözeltisi ve tüm insan kanı ürünlerini evrensel önlemleri alarak elleyin veya imha edin
- Solusyonları ağzınızla pipetlemeyin.
- Dökülenleri anında % 0.5 Sodyum Hipoklorit solüsyonu ile temizleyin.

5. Prensip

Spesifik anti-endotoksin antikorların mevcut olmaması halinde temel aktivite ölçümü (tüp 1), hasta nötrofillerinde belirli olmayan oksidatif faaliyeti belirler. Spesifik anti-endotoksin antikoru ve ekzojen endotoksin (tüp 3) fazlalığı içeren ilave kontrol ölçümleri, hastanın nötrofillerinin maksimum oksidatif faaliyet seviyesini ölçer. Test ölçümü (tüp 2), temiz endotoksin seviyesini ölçmek için spesifik antikorları içerir.. EAA sonuçları, test tüpündeki (tüp 2) deki kemiluminesansı, maksimum kemiluminesansla (tüp 3) normalleştirerek her iki ölçümü bazal kemiluminesans (tüp 1) için doğrultarak hesaplanır.

5.1 Numune Toplanışı ve Hazırlanışı

Bu prosedür için tam kan numunelerini almak için sadece EDTA anti-koagüle edilmiş kan alma tüplerini kullanınız. Hasta numunelerinin toplanışı , taşıması ve tahlili sırasında NCCLS[6] tarafından önerilen yönergeler takip edilmelidir. Numuneler, kalıcı kateter veya venipunktür yoluyla alınır ve EDTA anti-koagülan içeren kan toplama tüplerinde toplanır. r. Eğer numune alma işlemi kalıcı arteriyel veya venöz kanül ya da kateter ile yapılıyorsa, kanı EDTA anti-koagüle tüpe almadan önce hatları tesisinizin politikasına uygun şekilde temizleyiniz. Kan numuneleri, analizden önce 18°C – 25°C aralığındaki ortam sıcaklığında 180 dakikaya kadar muhafaza edilebilir. Kan, analizden hemen önce, en az 20-30 saniye boyunca yavaşça ters düz edilerek iyice karıştırılmalıdır.

Hastanın tahlilinin yapılabilmesi için hastanın tam kanından minimum 1.5 ml'nin alınması önerilmektedir (1.0 ml gereklidir). KK tahlininin yapılabilmesi için minimum 1.5 ml tam kanın alınması önerilmektedir (1.0 ml gereklidir). KK tahlili için gereken kan tam kan numunesinden bağımsız olabilir.

TR

5.2 Berthold Smartline Lüminometre Kullanım Talimatı

NOT: EAA™ veya EAA™ KK Tahliline başlamadan önce, Berthold SmartLine lüminometrenin "AÇIK" ve ısıtıcı inkübatör çalkalayıcısının "AÇIK" konumda olduğundan ve 37°C ± 1.0°C 'ye kadar ısınmış olduğundan emin olun.

5.2.1 EAA™ Test Prosedürü

İyi laboratuar uygulaması EAA tahlilinin her hasta için iki kez yapılmasını önermektedir. Yapılan iki tahlilinn ortalaması EAA™ sonucunu bildirmek için kullanılır. Her laboratuar bu tahlillerin geçerliliğinn kabulü için kabul edebilecek CV (belirsizlik katsayısını) kendileri geliştirip kullanmalıdır. Her tahlilde 3 adet hasta örneğine kadar analiz yapılabilir.

1. Her hasta numunesi için tutucuda istiflenmiş olan EAA™ tüplerinin kapaklarını ayırarak hazırlayınız.
2. 1.0 ml EAA™ Çözeltisini Tüp 1, 2 ve 3 içine bileşik pipet ile pipetle alınız (iki numune halinde).
3. Tüpleri ters çevirerek hastanın kanının karışmasını sağladıktan sonra, kalan miktardan 0.5 mL miktarı Aliquot Tüpüne ve LPS MAX etiketli tüplere pipetleyiniz. LPS MAX etiketli tüpü vorteksleyiniz.
4. Tutucuyu(lar) etüv sallayıcısına yerleştirip, kapağını kapattıktan sonra 10 dakika süre ile kuluçkaya yatırınız.
5. 10 Dakikalık kuluçlama süresini müteakipen kapağı açınız, Aliquot tüpünü vorteksleyiniz ve 40 µL miktardaki kanı 1 nolu ve 2 nolu (eş tüpleri de dahil) Tüplere steril bileşik pipetucu kullanarak pipetleyiniz.
6. LPS Max Tüpünü vorteksleyip, 40 µL miktardaki kanı 3 nolu (eş tüpü de dahil) Tüpe steril bileşik pipetucu kullanarak pipetleyiniz.
7. 1, 2 ve 3 nolu tüpleri vorteksleyin ve tekrar etüv sallayıcısının içindeki tutucuya(lara) yerleştirin, kapağı kapatın ve sallayıcıyı (100 devir/dakikaya ayarlanmış olarak) ve zamanlayıcıyı (14 dakikaya ayarlanmış olarak) başlatınız.
8. EAA etiketli akıllı kardı luminometer'e sokup Başlat düğmesine basınız.
9. 14 dakikalık kuluçlanma süresine müteakipen, EAA™ tüplerinin neticelerini sırası ile okuyabilmek için luminometer'ın göstergesindeki talimatları takip ediniz.
10. Her tüpü hafifçe vorteksleyerek, numune çekmecesini açarak tüpü numune tutucusuna yerleştiriniz, numune çekmecesini kapayarak RLU (relative light unit - bağıl ışık birimi) neticesini okuyabilmek için bekleyiniz. Aynı yöntemi diğer tüp için tekrarlayınız.
11. Tüm tüplerin değerleri okunduktan sonra EAA™ sonuçları bağlantılı olan yazıcıdan otomatik basılı olarak alınır.
12. Gerekiyorsa her hasta numunesi için bu yöntemi tekrarlayınız.

5.2.1.1 Sonuçların Yorumlanması

Aralık (EAA™ sonucu)	Sonuçların Yorumu
0.0 için 0.39	Düşük Endotoksin Seviyesi şiddetli sepsise doğru ilerleme riskinin düşük olduğunu gösterir.
0.40 için 0.59	orta derecede endotoksin aktivite seviyesi şiddetli sepsis'e doğru ilerleme riskinin arttığını gösterir.
≥ 0.60	yüksek derecede endotoksin aktivite seviyesinin mevcudiyeti şiddetli sepsis'in mevcudiyeti riskinin yüksek olduğunu gösterir.

5.2.2 EAA™ KK Test Prosedürü

KK testinin her EAA™ kit kutusu için bir kez yapılmasını önerir. Bununla beraber her laboratuar yerel standartlara bağlı olarak kendi kalite kontrol yöntemlerini geliştirebilir.

1. Kalite Kontrolü (KK) için kullanılacak tüpleri tutucuya yerleştiriniz ve kapaklarını açınız.
2. Her bir tüpün içerisine 1.0 ml EAA™ Çözeltisi kombipipetleyin.
3. 0.5 mL miktarında kalmış kanı KK Aliquot ve KK LPS MAX etiketli tüplere pipetleyiniz. LPS MAX etiketli tübü vorteksleyiniz.
4. Tutucuyu(lar) etüv sallayıcısına yerleştirin, kapağını kapadıktan sonre 10 dakika süre ile kuluçkaya yatırınız.
5. 10 Dakikalık kuluçlama süresini müteakipen kapağı açınız, KK Aliquot tübünü vorteksleyiniz ve 40 µL miktardaki kanı her iki 1 nolu ve Alt Kontrol Limiti Tüpüne steril bileşik pipetucu kullanarak pipetleyiniz.

TR

6. LPS Max Tübünü vorteksleyip, 40 µL miktardaki kanı once Üst Kontrol Limiti tüpüne daha sonra 3 nolu Tüpe steril bileşik pipetucu kullanarak pipetleyiniz.
7. Tüm tüpleri vorteksleyin ve tekrar etüv sallayıcısının içindeki tutucuya(lara) yerleştirin, ve sallayıcıyı (100 devir/dakikaya ayarlanmış olarak) ve zamanlayıcıyı (14 dakikaya ayarlanmış olarak) başlatınız.
8. EAA QC etiketli akıllı kardı luminometer'e sokup Başlat düğmesine basınız.
9. 14 dakikalık kuluçlanma süresine müteakipen, EAA QC tüplerinin neticelerini sırası ile okuyabilmek için luminometer'ın göstergesindeki talimatları takip ediniz.
10. Her tüpü hafifçe vorteksleyerek, numune çekmecesini açarak tüpü numune tutucusuna yerleştiriniz, numune çekmecesini kapayarak RLU (relative light unit - bağıl ışık birimi) neticesini okuyabilmek için bekleyiniz. Aynı yöntemi diğer tüp için tekrarlayınız.
11. Tüm tüplerin değerleri okunduktan sonra EAA™ QC sonuçları bağlantılı olan yazıcıdan otomatik olarak basılı olarak alınır.

TR

5.2.2.1 Sonuçların Yorumlanması

EAA™ Çözeltisi KK'ü aşağıdaki sonuçlarla doğrulanmıştır:

Sonuçların yorumlanması	EAA™ sonucu
EAA Alt QC	0.00 – 0.20
EAA Üst QC	0.80 – 1.50

Eğer Alt ve Üst Kontrol limitleri ile ilgili KK test sonuçları arzu edilen sonuçları vermiyor ise testleri tekrar ediniz. Eğer yeniden yapılan KK testleri sonuçları kabul edilen sınırlar dışında kalırsa müteakip EAA sonuçları geçersiz olabilir. O zaman lütfen, hemen EAA™ temsilcisi ile temasa geçiniz.

5.3 Sınırlamalar

5.3.1 Analitik

- Tüm immunoanaliz veya immunokimyasal reaksiyonlarında dikkatli bir teknik gereklidir.
- Kullanıcı, ilaçlar veya bilinmeyen ekzojen maddeler tarafından potansiyel etkileşimler sonucu ters etkilerin farkında olmalıdır. Aşağıdaki bağlantılı maddelere bakınız.

5.3.2 Klinik

- EAA™ sonuçları hastanın bütün klinik durumu ve tüm laboratuvar bulguları ile birlikte değerlendirilmelidir. Laboratuvar testlerinin klinik tabloya veya geçmişe uymadığı durumlarda, ek testler uygulanmalıdır.
- Daha önceki yayınlanmış çalışmalarda, ağır sepsisi olmayan hastalarda pozitif endotoksin bulgularına rastlandığı rapor edilmiştir.
- Onlar signficant klinik değeri olmayabilir olarak yoğun bakım ortamının dışında hastalarda elde edilen sonuçlar dikkatle yorumlanmalıdır.

TR

5.4 Klinik Performans

Kritik Hastalık (MEDIC) deneyinde Çok Merkezli i Endotoksin Saptaması, çok-merkezli prospektif gözlemsel çalışma Kuzey Amerika ve Avrupa'da ki akademik hastaneler kapsamında 10 Yoğun Bakım Ünitesinde yapılmıştır. Endotokseminin varlığı hastanın yoğun bakıma alınışını müteakipen 24 saati içinde ağır sepsisin gelişme ihtimalini belilemek için hastalarının ilk gününde değerlendirilmiştir. MEDIC deneyinde, risk değerlendirme çalışması için tüm uygun hastaları içeren hedef popülasyonda ki hastaların, yoğun bakıma girilen ilk günde değerlendirilebilir örneklere sahip olanları kaydedilmiştir N=857. Hasta demografileri aşağıdaki tabloda gösterilmiştir.

İncelenen Hastanın Demografik ve Bazal Özellikleri

Merkmal	
Yaş: ortalama (SD) ortanca	60 (±17) 62 [49, 74]
Cinsiyet: (%erkek)	58.9%
Irk (% kafkas olmayan)	15.8%
Apache II: ortalama (SD)	15.2 (±9,5) 14 [8, 21]
Yoğun bakımdaki gün: ortalama (SD)	5.4 (±10,9) 2.0 [1, 5]
Hastanedeki gün: ortalama	23.3 (±27,0) 14.0 [7, 29]
Yoğun bakım ölüm oranı	13.3%
Hastane ölüm oranı	19.9%

EAA™ testi için yoğun bakım hasta popülasyonu en uygun klinik ortamdır. EAA™ testinin benimsenmesi sadece yoğun bakım popülasyonu için ispatlanmıştır. Kritik hastalardaki, endotoksin seviyeleri ve ağır sepsisin gelişimi ihtimali arasındaki ilişki, yoğun bakımda yatan hastaların ilk günlerinde ağır sepsis riskini kanıtlamak için o anda mevcut bilgilere ilave olarak kullanılmıştır.

TR

5.5 Performans Özellikleri

5.5.1 Birbirlerine Aykırı Maddeler

- EAA ölçümleri ile 1000 mg/dL'ye kadar olan Trigliserid seviyeleri birbirleri ile aykırı düşmez. 1500 mg/dl'nin üzerindeki trigliserid konsantrasyonlarında EAA sonuçlarında bir düşüş olduğu görülmüştür. Fazlasıyla lipemik örneklerin EAA™ kullanımından kaçınması gerekir.
- 100 mg/dl seviyesindeki hemoglobin, EAA olçümlerini etkilemezken 500 mg/dl seviyesinde düşük EAA™ ölçümlerini 0.03'ten daha düşük EAA sonucuyla atenüe etmis ve yükselmiş EAA okuma degerlerini de ortalama yüzde 15 atenüe etmiştir. Ağır şekilde hemolize edilmiş numuneler EAA™'de kullanılmamalıdır.
- Hastalara, plasma albumini 10g/L den arttıran endotoksinsiz albüminin ekzojen infüzyonunu, EAA™ değerlerini düşürebilir. Hiper-protein anormalliğine sahip veya albümin tedavisine maruz görülen hastalardan numune alınmadan önce, yüksek seviyelerdeki albumin seviyelerinin EAA sonuçlarını saptırabilecek bir düzeyde olmaması için hastalar proteyn seviyelerini kontrol etmelidirler.

5.5.2 Hassasiyet

Tüm kan numuneleri, toplu ve eklenmiş ekzojen endotoksin ile, her üç alet üzerinde sekiz kez analiz edilmiştir. İşlem içi ve toplam numulerdeki tahlil hassasiyeti, EP5-A'da NCCLS prosedürü doğrultusunda hesaplanmıştır.

Probe	N	Ortalama EAA™	Içinde koşma		Topu kesinlik	
			1SD	%CV	1SD	%CV
1	24	0.11	0.015	14	0.023	22
2	24	0.20	0.030	15	0.029	14
3	24	0.30	0.043	15	0.042	14
4	24	0.50	0.059	12	0.064	13
5	24	0.52	0.036	7	0.034	6
6	24	0.59	0.050	8	0.046	8

5.5.3 Analitik Hassasiyet

Düşük EAA hasta numunesi EAA yönteminin hassasiyetini hesaplamak için kullanılmıştır. Yirmi dört saatlik EAA tayini, düşük EAA seviyeli tek bir hasta üzerinde yapılmıştır; her üç cihaz üzerinde sekiz ölçüm. Hastanın ortalama EAA seviyesi 0.11 ile 0.023 Standard Sapma arasındadır. u nedenle, iki standart sapma yöntemine göre hassasiyet, 0.046 EAA sonucu olarak hesaplanmıştır.

TR

5.5.4 Analitik Özellik

Gram negatif bakterilere ait endotoksin, E. coli O55:B5, P. aeruginosa, K. pneumoniae, S. enterides, E. coli 0127:B8 ve S. marcescens ve S. flexnevi EAA™ seviyelerinde 0.10 - 0.52'lik artışı tetiklemiştir kan numunesine 100 pg/mL endotoksin ilave edildiğinde 0.33 EAA sonucu elde edilmiştir. Ayrıca, V. cholerae LPS preparatları EAA sonuçlarında istatistiksel olarak anlamlı artışlar sağlamıştır. .. 2000 pg/mLye kadar lipoteikoik asit özünde, takip eden Gram pozitif soylarında reaktivite görülmemiştir: S.mutans, S.pyogenes, S.sanguis, S.faecalis, S.aureus ve B.subtilis. Benzer olarak, 2000 pg/mL'ye kadar mayalarda ve C.albicans ve A.fumagatis'in hücre duvarı özlerinde reaktivite gözlenmemiştir.

5.5.5 Sonuçların Doğruluğu

Tahlil düşük endotoksin seviyelerine karşı yüksek hassasiyet gösterir ve fazla yüksek endotoksin seviyesi tarafından teşvik edilmiş yüksek dozda çekme etkisine karşı dayanıklıdır. Bundan dolayı, Eğri, eğrisel bir şekil çizmektedir. Hasta numunelerinin düşük seviyede endotoksin içerdiği hallerde, endotoksin seviyesinde küçük artışlar EAA sonuçlarında anlamlı artışlara yol açacaktır. Yüksek EAA sonuçlarına neden olan numuneler için dilüsyon gerekmez.

6. Saklama ve Stabilite

EAA™ kitini ve bileşenlerini (Orijinal kapall pakette) 2-25°C'de muhafaza ediniz. Son kullanım tarihi geçen EAA™ kitlerini kullanmayınız.

7. Referanslar:

1. Van Deventer SJH, et al. Endotoxemia: an early predictor of septicemia in febrile patients. The Lancet (March 19) 1988; 605-609.
2. Parrillo JE, Parker MM, Natanson C, et al. Septic shock in humans: advances in the understanding of pathogenesis, cardiovascular dysfunction, and therapy. Ann Int Med 1990; 113: 227-242.
3. Zhao B, Bowden RAS, Stavchansky SA, et al. Human endothelial cell response to gram-negative lipopolysaccharide assessed with cDNA microarrays. Am J Cell Physiol 2001; 281: C1587-C1595.
4. Romaschin AD, et al. A rapid assay of endotoxin in whole blood using autologous neutrophil dependent chemiluminescence. J. of Immunology. Methods 1998; 212: 169-185.
5. NCCLS (National Committee for Clinical Laboratory Standards, 711 East Lancaster Avenue, Villanova, PA 19085) Document # M29-A Protection of Laboratory Worker from Instrument Biohazards and Infectious Disease Transmitted by Blood, Body Fluids and Tissues; Approved Guideline, 1997.
6. NCCLS (National Committee for Clinical Laboratory Standards, 711 East Lancaster Avenue, Villanova, PA 19085) Document # H3-#a Procedures for the Collection of Diagnostic Blood Specimens by Venipuncture – Third Edition; Approved Standard, 1991.

YALNIZCA IN VITRO TEŞHİS KULLANIMI İÇİNDİR

CE

Üretici:

Spectral Medical Inc.
135 The West Mall
Toronto, ON, Kanada M9C 1C2
+ 1.416.626.3233
FAKS: + 1.416.626.7383
www.spectraldx.com

Avrupa Yetkili Temsilcisi:

Dr. Rolf Keck
Ritterstraße 25
D-77977 Rust/Baden
Almanya
+ 49.782.286.5111
FAKS: + 49.782.286.5113

Endotoksin Aktivite Tahlili (EAA™) iSpectral Medical Inc.'in tescilli ticari markasıdır.

TR

EAA™

ENDOTOXIN ACTIVITY ASSAY

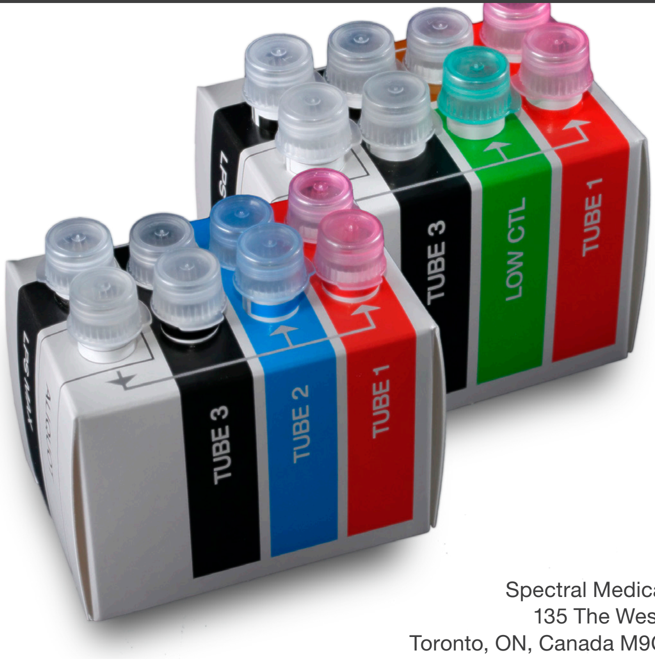

SPECTRAL Medical INC

Spectral Medical Inc.
135 The West Mall
Toronto, ON, Canada M9C 1C2
+ 1.416.626.3233
FAKS: + 1.416.626.7383
www.spectraldx.com

EAASTv1-03-091-3123_ML
