# Supplementary material for: Endotoxemia and circulating bacteriome in severe COVID-19 patients
Source: Intensive Care Med Exp. 2020 Dec 7;8:72. doi: 10.1186/s40635-020-00362-8 (PMC7719737; doi:10.1186/s40635-020-00362-8)

Assay for (1→3)-β-D-Glucan in Serum

FUNGITELL® ASSAY

Instructions For Use

ASSOCIATES OF CAPE COD INCORPORATED
124 Bernard E. Saint Jean Drive • E. Falmouth, MA 02536 USA

Telephone: (508) 540-3444
Toll-Free: (888) 395-2221
Fax: (508) 540-8680
Technical Support: (800) 848-3248
Customer Service: (800) 525-8378

Σ 42

CE

PN001268-en Rev10 2020-02-11

Visit Fungitell.com for instructions for use in your language.

INTENDED USE

The **Fungitell**® assay is a protease zymogen-based colorimetric assay for the qualitative detection of (1→3)-β-D-glucan in the serum of patients with symptoms of, or medical conditions predisposing the patient to, invasive fungal infection. The serum concentration of (1→3)-β-D-glucan, a major cell-wall component of various medically important fungi[1], can be used as an aid in the diagnosis of deep-seated mycoses and fungemias[2]. A positive result does not indicate which genus of fungi may be causing infection.

(1→3)-β-D-glucan titers should be used in conjunction with other diagnostic procedures, such as microbiological culture, histological examination of biopsy samples and radiological examination.

> **IMPORTANT Provide this information to the requesting physician:** Certain fungi, such as the genus *Cryptococcus* which produces very low levels of (1→3)-β-D-glucan, may not result in serum (1→3)-β-D-glucan sufficiently elevated as to be detected by the assay[3,4]. Infections with fungi of the Order Mucorales such as *Absidia, Mucor* and *Rhizopus*[1,4] which are not known to produce (1→3)-β-D-glucan, are also observed to yield low serum (1→3)-β-D-glucan titers. In addition, the yeast phase of *Blastomyces dermatitidis* produces little (1→3)-β-D-glucan and may not be detected by the assay[5].
>
> **Include this statement when reporting the Fungitell® assay test results.**

SUMMARY AND EXPLANATION

There is an increasing incidence of fungal infections by opportunistic pathogens, especially in immuno-compromised patients[6,7,8]. Invasive fungal diseases, as opportunistic infections, are common among hematological malignancy and AIDS patients and account for a growing number of nosocomial infections, particularly among organ transplant recipients and other patients receiving immunosuppressive treatments[9,10]. Many fungal diseases are acquired by inhaling fungal spores originating from the soil, plant detritus, air-handling systems and/or exposed surfaces. Some opportunistic fungi are present in/on human skin, the intestinal tract, and mucous membranes[11,12]. Diagnosis of invasive mycoses and fungemias is usually based on non-specific diagnostic or radiological techniques. Recently, biological markers of fungal infection have been added to the available diagnostic methods[2].

Opportunistic fungal pathogens include *Candida spp., Aspergillus spp., Fusarium spp., Trichosporon spp., Saccharomyces cerevisiae, Acremonium spp., Coccidioides immitis, Histoplasma capsulatum, Sporothrix schenckii, Exserohilum rostratum,* and *Pneumocystis jirovecii.* The (1→3)-β-D-glucan produced by these organisms, and others, can be detected by the Fungitell® assay[1,8,13,14].

PRINCIPLE OF THE PROCEDURE

The Fungitell® assay measures (1→3)-β-D-glucan. The assay is based upon a modification of the *Limulus* Amebocyte Lysate (LAL) pathway[15,16,17,18], Figure 1. The Fungitell® reagent is modified to eliminate bacterial endotoxin reactivity and, thus, to only react to (1→3)-β-D-glucan, through the Factor G-mediated side of the pathway. (1→3)-β-D-glucan activates Factor G, a serine protease zymogen. The activated Factor G converts the inactive pro-clotting enzyme to the active clotting enzyme, which in turn cleaves para-nitroanilide (pNA) from the chromogenic peptide substrate, Boc-Leu-Gly-Arg-pNA, creating a chromophore, para-nitroaniline, that absorbs at 405 nm. The Fungitell® kinetic assay, described below, is based upon the determination of the rate of optical density increase produced by a sample. This rate is interpreted against a standard curve to produce estimates of (1→3)-β-D-glucan concentration in the sample.

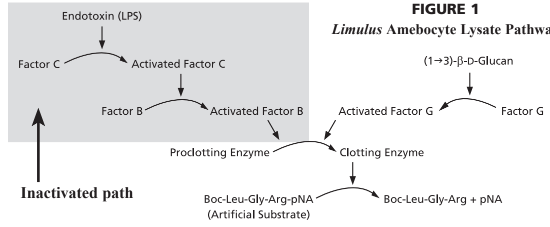

FIGURE 1
***Limulus* Amebocyte Lysate Pathway**

MATERIALS SUPPLIED WITH THE FUNGITELL® KIT

The Fungitell® kit is for in vitro diagnostic use. The following materials supplied with each kit are sufficient to assay 110 wells on two microtiter plates (55 wells on each):

1. Fungitell® Reagent, a lyophilized (1→3)-β-D-glucan specific LAL (two vials)
2. Pyrosol® Reconstitution Buffer (two vials). Additional vials of Pyrosol® Reconstitution Buffer (catalog number BC051) may be purchased separately.
3. Glucan standard, with the (1→3)-β-D-glucan content stated on the label (two vials)
4. Reagent Grade Water (RGW) (two bottles)
6. Alkaline Pre-treatment Solution (two vials)

All of the above, with the exception of the standard, are free of interfering levels of (1→3)-β-D-glucan.

MATERIALS REQUIRED BUT NOT SUPPLIED

All materials must be free of interfering glucan. Glassware must be dry-heat depyrogenated for at least 7 hours at a minimum of 235°C (or a validated equivalent) to be considered suitable for use.

1. Pipette tips* (250 µL - Cat# PPT25, 1000 µL - Cat# PPT10)
2. Pipettes capable of delivering 5-25 µL and 100-1000 µL volumes
3. Repeating pipette with syringe tips capable of delivering 100 µL
4. Test tubes* for standard series (calibration curve) preparation and combining serum treatment reagents. (12 x 75 mm - Cat# TB240 or 13 x 100 mm - Cat# TB013)
5. Incubating (37°C) plate reader capable of reading at 405 nm (preferably capable of dual wavelength monitoring at both 405 and 490 nm) with a dynamic range up to, at least 2.0 Absorbance Units, coupled with appropriate computer-based kinetic assay software.
6. Sterile, glucan-free, screw-cap storage tubes for aliquotting samples (most tubes that are certified to be RNAse, DNAse, and pyrogen-free are free of interfering levels of (1→3)-β-D-glucan).
7. Parafilm®
8. 96 well Microplates* Note: The Fungitell® assay requirements have has been validated with plates that have the following characteristics: Polystyrene, sterile, uncoated, flat-bottomed, no interfering beta glucan to ACC specifications and individually wrapped.

* These products, supplied by Associates of Cape Cod, Inc. (ACC), are certified free of interfering glucans

Caution - glass pipettes with cotton plugs and micro-pipette tips with cellulosic filters are potential sources of glucan contamination.

WARNINGS AND PRECAUTIONS

This product is for* IN VITRO *DIAGNOSTIC USE.

> The Fungitell® assay requires rigorous attention to technique and the testing environment. Thorough training of the technician in the assay method and in the avoidance of contamination is critical for the effectiveness of the assay.

1. Certain fungal species produce very low levels of (1→3)-β-D-glucan and are not usually detected by the Fungitell® assay. These include the genus *Cryptococcus*[3,4] as well as *Mucorales* such as *Absidia, Mucor* and *Rhizopus*[1,4]. In addition, *Blastomyces dermatididis*, in its yeast form, produces low levels of (1→3)-β-D-glucan and is therefore not usually detected by the Fungitell® assay[5].
2. Do not pipette any material by mouth. Do not smoke, eat or drink in areas where specimens or kit reagents are handled. Follow institution and local safety regulations.
3. Establish a clean environment in which to perform the assay. Use materials and reagents that are certified to be free of detectable background levels of (1→3)-β-D-glucan. Note that glucan as well as fungal contamination from the human body, clothes, containers, water and airborne dust may cause interference with the Fungitell® assay. Cellulosic materials such as gauze, paper wipes, and cardboard may contribute (1→3)-β-D-glucan to the environment where the assay is performed.
4. Do not use reagents beyond their expiry date.
5. Off-color or turbid samples such as those that are grossly hemolyzed, lipemic, or contain excessive bilirubin may cause optical interference with the assay. If such samples are tested, test results should be examined for evidence of optical interference and/or unusual kinetic patterns.
6. Use suitable protective clothing and powder free gloves when handling patient specimens.
7. The serum of hemodialysis patients may contain high levels of (1→3)-β-D-glucan when certain cellulose dialysis membranes are used[19,20,38]. Hemodialysis with cellulose triacetate, polysulfone membrane or polymethyl methacrylate membranes does not appear to interfere with the assay.
8. Surgical gauzes and sponges can leach high levels of (1→3)-β-D-glucan that may contribute to a contamination-based transient positive result for the Fungitell® assay as has been observed in post-surgical patients[21,22].
9. Blood fractionation products such as intravenous immunoglobulin and albumin may also have burdens of (1→3)-β-D-glucan which, if injected or infused, will elevate serum (1→3)-β-D-glucan titers for a number of days[23].
10. Kits with damaged contents should not be used.
11. Materials exposed to potentially contaminated (pathogen-containing) fluids must be disposed of in a manner consistent with local regulation.

REAGENT STORAGE

Store all reagents, as supplied, at 2-8°C in the dark. Reconstituted Fungitell® reagent should be stored at 2-8°C and used within 2 hours. Alternatively, reconstituted Fungitell® reagent can be frozen at -20°C for up to 20 days, thawed once and used.

SPECIMEN HANDLING

1. Specimen Collection: Blood samples may be collected in sterile serum preparation tubes or serum separator tubes (SST) for the preparation of serum.
2. Specimen Storage: Serum samples can be stored temporarily at 2-8°C before assay, or frozen at -20°C or colder for longer term storage.
3. Specimen Labeling: Specimens should be clearly labeled according to the approved practices of the institution.

PROCEDURE

Note: Settings may vary with different instruments and software. In general, the following will apply: Set the platereader software to collect data in the ^{V}mean mode. Check the software manual for the proper settings to ensure that the value calculated is the mean rate of optical density change for all of the data points gathered. Set the detector read interval to the minimum allowed by the software/instrument over the 40 minute period of the test. The software wavelength settings should be 405 nm minus the background at 490 nm. If dual wavelength reading is not available, read the test at 405 nm. The incubation temperature is to be set at 37°C. Set mixing/plate shaking to occur for 5 – 10 seconds prior to the commencement of reading. Select the curve fit setting to "linear/linear" or equivalent. Reading should commence without any lag time.

1. Preparation of glucan standard provided in the kit.
   a. Dissolve one vial of the glucan standard with the volume of RGW stated on the vial, to make a 100 pg/mL solution. Vortex at least 30 seconds at medium to medium-high speed to reconstitute the standard (solution 1). The glucan solution should be stored at 2-8°C and used within three days. Steps b - e listed below illustrate an example of a standard curve preparation scheme.
   b. Prepare 50 pg/mL standard (solution 2) by mixing 500 µL RGW and 500 µL of solution 1 in a glucan-free tube (solution 2). Vortex for at least 10 seconds.
   c. Prepare 25 pg/mL standard (solution 3) by mixing 500 µL RGW and 500 µL of solution 2 in a glucan-free tube (solution 3). Vortex for at least 10 seconds.
   d. Prepare 12.5 pg/mL standard (solution 4) by mixing 500 µl RGW and 500 µl of solution 3 in a glucan-free tube (solution 4). Vortex for at least 10 seconds.
   e. Prepare 6.25 pg/mL standard (solution 5) by mixing 500 µl RGW and 500 µL of solution 4 in a glucan-free tube (solution 5). Vortex for at least 10 seconds.
2. Open the Alkaline Pretreatment Solution. The Alkaline Pretreatment Solution converts triple-helix glucans into single-stranded glucans[17,18] which are more reactive in the assay.Additionally, the alkaline pH serves to inactivate serum proteases and inhibitors that can interfere with the assay[24].

   Discard the vial (in accordance with laboratory procedures) unless it is going to be used in a subsequent test, in which case, cover it with Parafilm, using the side of the Parafilm that faced the paper backing.
3. Enter the standard concentrations into the software settings as 500, 250, 125, 62.5, and 31 pg/mL, respectively.
   Note that the standard concentrations entered are five times greater than those prepared in item 1. above. This is because the volume of standard used in the assay is 25 µL per well, which is five times the volume of the serum sample used (see item 4.b. below).
   Set up the microtiter plate layout in the software, with the standards (St), negative controls (Neg) and 21 unknowns (Uk) each assayed in duplicate as follows:
   Note 1: The outside wells may be used, if it has been demonstrated that the performance of the outside wells is comparable to that of the internal wells.
   Note 2: To avoid accidental contamination, replace the cover on the microplate after adding samples and reagents to the wells. Remove the cover before placing the plate in the reader to avoid optical interference from condensation.
   Note 3: The negative controls are not utilized in the standard curve.
4. Serum and Alkaline Pretreatment Solution addition.
   a. Thaw frozen serum samples at room temperature. Vortex all samples well – for at least 30 seconds on medium to medium-high speed setting.
   b. Transfer 5 µl of the serum sample to each of its designated wells (Uk) in at least duplicate. Repeat for each serum sample.
   c. Add 20 µl of the Alkaline Pretreatment Solution to each well containing serum. Ensure that the serum and the pre-treatment droplets come in contact with one another.
   Note: Steps b and c can be conducted in reverse order according to technician preference.
   d. Agitate the plate for 5 – 10 seconds to mix the well contents (the reader's plate agitation function may be used) then incubate for 10 minutes at 37°C in the incubating plate reader.
5. Reconstitution of Fungitell® reagent. Note: This may be conveniently performed while the pre-treatment incubation is in progress. Consistency of reconstitution timing will enhance reproducibility as the Fungitell® reaction begins upon reconstitution, albeit at a low level.
   a. Reconstitute one vial of Fungitell® reagent by adding 2.8 mL of RGW and then adding 2.8 mL of Pyrosol Reconstitution buffer using the 1000 µL pipette. Cover the vial with Parafilm using the side of Parafilm that faced the paper backing. Swirl the vial gently to dissolve completely – do not vortex.
6. Addition of negative controls and glucan standards. At the end of serum pre-treatment incubation (step 3. d), remove the plate from the incubating plate reader and add the standards and negative controls to the plate. Recommended standard concentration pattern:
   a. Add 25 µL of RGW to wells G2 and G3.
   b. Add 25 µL of the 6.25 pg/mL standard solution 5 to wells F2 and F3.
   c. Add 25 µL of the 12.5 pg/mL standard solution 4 to wells E2 and E3.
   d. Add 25 µL of the 25 pg/mL standard solution 3 to wells D2 and D3.
   e. Add 25 µL of the 50 pg/mL standard solution 2 to wells C2 and C3.
   f. Add 25 µL of the 100 pg/mL standard solution 1 to wells B2 and B3.
7. Fungitell® reagent addition and plate incubation procedure.
   a. Add 100 µL of Fungitell® reagent to each well (containing negative controls, standards, and samples) using the stepper (repeater) pipette.
   b. Insert the plate into the microplate reader (equilibrated to 37°C) with the lid on and shake for 5 – 10 seconds. Read the plate without the lid at 405 nm minus 490 nm, for 40 minutes at 37°C. Note: If the instrument does not allow time to take the lid off between shaking and reading, shake with lid off to ensure reading **without the lid** on. If background subtraction (at 490 nm) is unavailable, it is acceptable to read at 405 nm. If a plate shaking function is unavailable with the microplate reader, an external microplate shaker may be used.
   c. Collect the data and analyze as follows: Examine optical density plots of test samples and check for kinetic patterns other than a smooth increase comparable to those of standards. Invalidate plots indicating optical interference (e.g. their kinetic patterns do not follow those of the standards). Calculate the mean rate of optical density change (milli-absorbance units per minute) for all points between 0 and 40 minutes (performed by the software). Interpolate the sample (1→3)-β-D-glucan concentrations from the standard curve (performed by the software).

INTERPRETATION OF RESULTS

The Fungitell® test results should be used as an aid in the diagnosis of invasive fungal infection. The results are expressed in pg/mL of serum and range from non-detectable (<31 pg/mL) to >500 pg/mL and are printed out by the software or read from the standard curve. Accurate values above 500 pg/mL require that the sample be diluted with RGW and retested.

The laboratory performing the test should inform the ordering physician that not all fungal infections result in elevated levels of serum (1→3)-β-D-glucan. Some fungi, such as the genus *Cryptococcus*[3,4], produce very low levels of (1→3)-β-D-glucan. *Mucorales,* such as *Absidia*, *Mucor* and *Rhizopus*[1,4] are not known to produce (1→3)-β-D-glucan. Similarly, *Blastomyces dermatitidis*, in its yeast phase, produces little (1→3)-β-D-glucan, and blastomycosis patients usually have undetectable levels of (1→3)-β-D-glucan in the Fungitell® assay[5].

NEGATIVE RESULT

(1→3)-β-D-glucan values <60 pg/mL are interpreted as negative results.

INDETERMINATE RESULT

Values from 60 to 79 pg/mL suggest a possible fungal infection. Additional sampling and testing of sera is recommended. Frequent sampling and testing improves the utility for diagnosis.

POSITIVE RESULT

A positive result means that (1→3)-β-D-glucan values are ≥ 80 pg/mL are interpreted as a positive result. A positive result means that (1→3)-β-D-glucan was detected. A positive result does not define the presence of disease and should be used in conjunction with other clinical findings to establish a diagnosis.

QUALITY CONTROL

- The correlation coefficient (r) of the standard curve (linear vs. linear) should be ≥ 0.980.
- The wells with 25 µL of RGW are the negative controls. Negative controls should have actual optical density rate (milli-absorbance units per minute) values less than 50% of the lowest standard. If not, the assay should be repeated using all new reagents.
- Handling problem samples. If the analyst observes unusual optical density kinetics in a test of a sample that is cloudy, off-color, or turbid (such as those that are grossly hemolyzed, lipemic or contain excessive bilirubin), the sample must be diluted with RGW and retested. The dilution must be accounted for in the reporting of results by multiplying the result by the dilution factor. Typically, the dilution factor is entered in the software setup for the sample and the correction is automatically applied.
- Control samples, at cut-off and highly positive levels, may be run to verify that the reagents and the assay are performing properly. Each user of the test should establish a quality control program to assure proficiency in the performance of the test in accordance with the regulations applicable to their location.

LIMITATIONS OF THE TEST

1. The tissue locations of fungal infection (10), encapsulation, and the amount of (1→3)-β-D-glucan produced by certain fungi may affect the serum concentration of this analyte. Reduced ability to contribute (1→3)-β-D-glucan to the bloodstream can reduce the ability to detect certain fungal infections. *Cryptococcus spp.* produce low levels of (1→3)-β-Dglucan[1,4]. *Mucorales*, including *Absidia spp*., *Mucor spp*. and *Rhizopus spp*. are not known to produce (1→3)-β-D-glucan[1,4]. *Blastomyces dermatitidis*, in its yeast phase, produces little (1→3)-β-D-glucan, and test results are usually negative[5].

2. Some individuals have elevated levels of (1→3)-β-D-glucan that fall into the indeterminate zone. In such cases, additional surveillance testing is recommended.

3. The frequency of patient testing will depend upon the relative risk of fungal infection. Sampling rates of at least two to three times per week are recommended for at risk patients.

4. Positive results have been found in hemodialysis patients[19,20], subjects treated with certain fractionated blood products such as serum albumin and immunoglobulins[25] and in specimens or subjects exposed to glucan-containing gauze and surgical sponges. Patients require 3 – 4 days for the restoration of baseline levels of serum (1→3)-β-D-glucan, after surgical exposure to (1→3)-β-D-glucan containing sponges and gauze[21,22]. Accordingly, the timing of sampling of surgical patients should take this into account.

5. Samples obtained by heel or finger stick methods are unacceptable as the alcohol-soaked gauze used to prepare the site (and, potentially, the skin surface-pooling of blood) has been shown to contaminate the specimens. In studies to date, no differences have been observed between samples obtained by line draws or venipuncture[26,27].

6. Test levels were established in adult subjects. Infant and pediatric normal and cut-off levels are under investigation[28,29].

7. The reportable range of the assay is 31 pg/mL to 500 pg/mL. Values below 31 pg/mL are to be reported as <31 pg/mL. Values greater than 500 pg/mL are to be reported as >500 pg/mL, unless the sample has been diluted.

INTERFERING SUBSTANCES

The following sample conditions can interfere with an accurate Fungitell® assay result:

- Hemolysis
- Sample turbidity caused by lipemia
- The presence of visually apparent bilirubin
- Turbid serum
- Elevated levels of Immunoglobulin G, such as may exist in the serum due to multiple melanoma, may result in precipitation in the reaction mixture upon the addition of Fungitell® to the pre-treated serum[30].

EXPECTED VALUES

Beta glucan values are elevated in a variety of fungal infections. When signs and symptoms are present at the 80 pg/mL level or greater, the predictive value that the subject is positive for a fungal infection ranges from 74.4 to 91.7%. In the absence of signs and symptoms at less than 60 pg/mL, the negative predictive values ranged from 65.1% to 85.1%.

PERFORMANCE CHARACTERISTICS

Comparison Testing

A multi-center, prospective study to validate the performance characteristics of the Fungitell® assay was conducted[31]. The test was compared to other standard methods of detection, (i.e., blood culture, histopathological examination of biopsy specimen and radiological signs) for mycoses and fungemias.

Three hundred and fifty-nine (359) subjects were tested by the assay. A single sample was obtained from each subject. The low risk subjects included apparently healthy individuals and those at the clinical sites who were admitted to hospitals for reasons other than fungal infections. Subject accrual was conducted at six clinical sites in the United States. Four of the clinical sites performed the assay and tested a total of 285 samples. ACC tested all 359 samples twice but only used the second set of results to determine the assay performance. The results of the second set of analyses were not statistically different from the first set.

The sensitivity for the entire subject population (359) including cryptococcosis patients was 65.0% ([0.1 - 70.0% 95% Confidence Interval (CI)]. The specificity was 81.1% (77.1 - 85.2% CI) (Table 1).

Table 1 ACC Test Results at the 60-80 pg/mL Cutoff level by Site

Site	Proven/Probable **Sensitivity** >=80pg/mL			**Specificity** <60pg/mL			Equivocal 60<=X<80	Total
	Pos/ Clin. Pos	**Sensitivity**	Positive Predictive Value	Neg/ Clin. Neg	**Specificity**	Negative Predictive Value		
1	32/50	64.0	97.0	39/40	97.5	69.6	1	90
2	14/24	58.3	93.3	17/20	85.0	70.8	5	44
3	14/19	73.7	46.7	36/54	66.7	90.0	3	73
4	25/33	75.8	92.6	37/43	86.0	86.0	6	76
5	21/36	58.3	80.8	30/39	76.9	69.8	6	75
6	0/1	0.0	N/A	0/0	N/A	0.0	0	1
Total	106/163	65.0	80.9	159/196	81.1	76.8	21	359

When the results obtained by ACC (359 samples) and by the clinical sites (285 sample) are compared to clinical diagnosis, the sensitivity is 64.3% (58.8% - 69.9% CI) for ACC and 61.5% (55.9% - 67.2% CI) for the sites. The specificity is 86.6% (82.7% - 90.6% CI) for ACC versus 79.6% (74.9% - 84.3% CI) for the sites.

CANDIDIASIS

There were 107 subjects who were positively diagnosed with candidiasis in the prospective study. 83 of the 107 were positive by the Fungitell® assay.

One hundred seventy-five candidiasis library samples were furnished to Associates of Cape Cod, Inc. 145 of the 175 were positive by the assay.

ASPERGILLOSIS

A total of 10 subjects were positive for aspergillosis. 8 of the 10 were positive by the assay.

FUSARIOSIS

Three subjects were positive for fusariosis. 2 of the 3 were positive by the assay.

ANTI-FUNGAL DRUG THERAPY

The presence or absence of antifungal drug therapy had no statistically significant effect upon assay sensitivity. 118 subjects were proven positive for invasive fungal infection and on anti-fungal therapy. 82 were positive by the assay (sensitivity, 69.5%; 61.2% - 77.8% CI). In addition, twenty-four (24) subjects were proven positive, but not on any anti-fungal therapy. 18 were positive by the assay (sensitivity, 75%; 57.7% - 92.3% CI).

SPECIFICITY

A total of 170 subjects were negative for fungal infection and were apparently healthy individuals. The specificity was 86.5% with the assay (82.8% - 90.1% C.I.). When the additional 26 subjects who were negative for fungal infection but with other disorders were included, an 81.1% specificity was observed (77.1 - 85.2 % CI).

TEST CORRELATIONS

Four of the clinical sites assayed a total of 285 samples. The site test results correlated quantitatively at 96.4% with the Associates of Cape Cod, Inc. results. The Associates of Cape Cod, Inc. correlations with the different testing sites ranged from 90.6 to 99.2%.

PRECISION

In the Precision Studies, ten (10) different samples were each tested by three testing sites, on three different days. The intra-assay variation ranged from 0.9 to 28.9%. The Inter Assay values ranged from 3.9 to 23.8%. The four (4) negative samples were excluded from both analyses.

META-ANALYSES

In addition, numerous peer-reviewed studies have been published on the subject of serum (1→3)-β-D-glucan-based support for invasive fungal disease diagnosis including meta-analyses of diagnostic performance[32,33,34,35,36,37].

SYMBOLS LEGEND

	"Use By"		"Temperature Limitation"
Σ N	"Contains Sufficient For 'N' Tests"		"Manufacturer"
LOT	"Batch Code"		"Consult Instructions For Use"
IVD	"In Vitro Diagnostic Medical Device"	EC REP	"Authorised Representative"
REF	"Catalogue No."	CE	"CE Mark"

Rx only "For prescription use only"

Associates of Cape Cod Europe GmbH, Opelstrasse 14, D-64546 Mörfelden-Walldorf, Germany

Australian Sponsor: Emergo Australia, Level 20, Tower II, Darling Park, 201 Sussex Street Sydney, NSW 2000, Australia

REFERENCES

1. Odabasi, Z., Paetznick, V., Rodriguez, J., Chen, E., McGinnis, M., and Ostrosky-Zeichner, L. 2006. Differences in beta-glucan levels of culture supernatants of a variety of fungi. Medical Mycology 44: 267-272.
2. De Pauw, B., Walsh, T.J., Donnelly, J.P. et al. 2008. Revised definitions of invasive fungal disease from the European Organization for Research and Treatment of Cancer/Invasive Fungal Infections Cooperative Group and the National Institutes of Allergy and Infectious disease Mycosis Study Group (EORTC/MSG) Concensus Group. Clin. Inf. Dis. 46: 1813-1821.
3. Miyazaki, T., Kohno, S., Mitutake, K., Maesaki, S., Tanaka, K-I., Ishikawa, N., and Hara, K. 1995. Plasma (1→3)-ß-D-Glucan and fungal antigenemia in patients with candidemia, aspergillosis, and cryptococcosis. J. Clinical Microbiol. 33: 3115-3118.
4. Binder, U., Maurer, E., and Lass-Florl, C. 2014. Mucormycosis – from the pathogens to the disease. Lin. Microbiol. Infect. 20 (Suppl.6): 60-66.
5. Girouard, G., Lachance, C., and Pelletier, R. 2007. Observations of (1→3)-ß-D-Glucan detection as a diagnostic tool in endemic mycosis caused by *Histoplasma* or *Blastomyces*. J. Med. Mycology 56: 1001-1002.
6. Walsh, T.J., Groll, A.H. Emerging fungal pathogens: evolving challenges to immunocompromised patients for the twenty-first century. Transpl. Infectious Dis. 1999: 1:247-261.
7. Fishman, J.A., Rubin, R.H. Infection in organ-transplant recipients. New England Journal of Medicine. 1998: 338:1741-1751.
8. Obayashi, T., Yoshida, M., Mori, T., Goto, H. Yasuoka, A., Iwasaki, H., Teshima, H., Kohno, S., Horichi, A., Ito, A., Yamaguchi, H., Shimada, K., and Kawai, T. 1995. Plasma (1→3)-ß-D-Glucan measurement in diagnosis of invasive deep mycosis and fungal febrile episodes. Lancet. 345: 17-20.
9. Fridkin, S.K. and Jarvis, W.R. 1996. Epidemiology of nosocomial fungal infections. Clin. Micro. Rev. 9: 499-511.
10. Alexander, B., Diagnosis of fungal infection: new technologies for the mycology laboratory. Transpl. Infectious Dis. 2002: 4 (Suppl. 3):32-37
11. Lass-Florl, C. 2009. The changing face of epidemiology of invasive fungal disease in Europe. Mycoses. 52: 197-205.
12. Nucci, M. and Anaissie, E. 2009. Fungal infections in hematopoietic stem cell transplantation and solid organ transplantation - Focus on aspergillosis. Clin. Chest Med. 30: 295-306.
13. Litvintseva, A.P., Lindsley, M.D., Gade, L., Smith, R., Chiller, T., Lyons, J.L., Thakur, K.T., Zhang, S.X., Grgurich, D.E., Kerkering, T.M., Brandt, M.E., and Park, B,J. Utility of (1-3)-β-D-glucan testing for diagnostics and monitoring response to treatment during the multistate outbreak of fungal meningitis and other infections. J. Clin. Microbiol. 2015; 53:618-25.
14. Odabasi, Z., Mattiuzzi, G., Estey, E., Kantarijian, H., Saeki, F., Ridge, R., Ketchum, P., Finkelman, M., Rex, J., and Ostrosky-Zeichner, L. 2004. ß-Glucan as a diagnostic adjunct for invasive fungal infections: Validation, cut-off development, and performance in patients with Acute Myelogenous Leukemia and Myelodysplastic Syndrome. CID 39: 199-205.
15. Iwanaga, S., Miyata, T., Tokunaga, F., and Muta, T. 1992. Molecular mechanism of hemolymph clotting system in *Limulus*. Thrombosis Res. 68: 1-32.
16. Tanaka, S., Aketagawa, J., Takahashi, S., Tsumuraya, Y., and Hashimoto, Y. 1991. Activation of a *Limulus* coagulation factor G by (1→3)-ß-D-Glucans. Carbohydrate Res. 218:167-174.
17. Saito, H., Yoshioka, Y., Uehara, N., Aketagawa, J., Tanaka, S., and Shibata, Y. 1991. Relationship between conformation and biological response for (1→3)-ß-D-Glucans in the activation of coagulation factor G from *Limulus* amebocyte lysate and host-mediated antitumor activity. Demonstration of single-helix conformation as a stimulant. Carbohydrate Res. 217:181-190.
18. Aketagawa, J., Tanaka, S., Tamura, H., Shibata, Y., and Saito, H. 1993. Activation of *Limulus* coagulation factor G by several (1→3)-ß-D-Glucans: Comparison of the potency of glucans with identical degree of polymerization but different conformations. J. Biochem 113:683-686.
19. Kanda, H., Kubo, K., Hamasaki, K., Kanda, Y., Nakao, A., Kitamura, T., Fujita, T., Yamamoto, K., and Mimura, T. 2001. Influence of various hemodialysis membranes on the plasma (1→3)-ß-D-Glucan level. Kidney International 60: 319-323.
20. Kato, A., Takita, T, Furuhashi, M., Takahashi, T., Maruyama, Y., and Hishida, A. 2001. Elevation of blood (1→3)-ß-D-Glucan concentrations in hemodialysis patients. Nephron 89:15-19.
21. Kanamori, H., Kanemitsu, K., Miyasaka, T., Ameku, K., Endo, S., Aoyagi, T., Inden, K., Hatta, M., Yamamoto, N., Kunishima, H., Yano, H., Kaku, K., Hirakat, Y., and Kaku, M. 2009. Measurement of (1→3)-ß-D-Glucan derived from different gauze types. Tohoku J. Exp. Med. 217: 117-121.
22. Mohr, J., Paetznick, V., Rodriguez, J., Finkelman, M., Cocanour, C., Rex, J., and Ostrosky-Zeichner, L. 2005. A prospective pilot survey of ß-glucan (BG) seropositivity and its relationship to invasive candidiasis (IC) in the surgical ICU (SICU) ICAAC Poster #M-168.
23. Held J, Wagner D.β-d-Glucan kinetics for the assessment of treatment response in *Pneumocystis jirovecii* pneumonia. Clin Microbiol Infect. 2011;17:1118-22.
24. Tamura, H., Arimoto, Y., Tanaka, S., Yoshida, M., Obayashi, T, and Kawai, T. 1994. Automated kinetic assay for endotoxin and (1→3)-ß-D-Glucan in human blood. Clin. Chim. Acta 226: 109-112.
25. Ogawa, M., Hori, H., Niiguchi, S., Azuma, E., and Komada, Y. 2004. False positive plasma (1→3)-ß-D-Glucan following immunoglobulin product replacement in adult bone marrow recipient. Int. J. Hematol. 80: 97-98.
26. Racil, Z., Kocmanova, I., Lengerova, M., Weinbergerova, B., Buresova, L., Toskova, M., Winterova, J., Timilsina, S., Rodriguez, I., and Mayer, J. Difficulties in using 1,3-{beta}-D-glucan as the screening test for the early diagnosis of invasive fungal infections in patients with haematological malignancies--high frequency of false-positive results and their analysis. J. Med. Microbiol. 2010; 59:1016-22.
27. Posteraro B., De Pascale, G., Tumbarello, M., Torelli, R., Pennisi,M.A., Bello, G., Maviglia, R., Fadda, G., Sanguinetti, M., and Antonelli, M. 2011 Early diagnosis of candidemia in intensive care unit patients with sepsis: a prospective comparison of (1→3)-β-D-glucan assay, *Candida* score, and colonization index. Crit Care.15: R249.
28. Smith, P.B., Benjamin, D.K., Alexander, B.D., Johnson, M.D., Finkelman, M.A., and Steinbach, W.J. 2007. (1→3)-ß-D-Glucan levels in pediatric patients: Preliminary data for the use of the beta-glucan test in children. Clin. Vaccine Immunol. 14: 924-925.
29. Goudjil. S., Kongolo, G., Dusol, L., Imestouren, F., Cornu, M., Leke, A., and Chouaki, T. 2013. (1→3)-β-D-glucan levels in candidiasis infections in the critically ill neonate. J. of Matenernal-Fetal and Neonatal Med. 26: 44-48.
30. Issa, N.C., Koo, S., Lynch, R.C., Gay, C., Hammond, S.P., Baden, L.R., Ghobrial, I.M., Finkelman, M.A., and Marty, F.M... 2012 Serum galactomannan and (1->3)-β-D-glucan assays for patients with multiple myeloma and Waldenstrom's macroglobulinemia. J.Clin. Microbiol. 50:1054-6.
31. Ostrosky-Zeichner, L., Alexander, B.D., Kett, D.H., Vazquez, J., Pappas, P.G., Saeki, F., Ketchum, P.A., Wingard, J., Schiff, R., Tamura, H., Finkelman, M.A., Rex, J.H. 2005. Multicenter clinical evaluation of the (1→3)-ß-D-Glucan assay as an aid to diagnosis of fungal infections in humans. Clin. Inf. Dis. 41: 299-305.
32. Karageorgopoulos DE, Vouloumanou EK, Ntziora F, Michalopoulos A, Rafailidis PI, Falagas ME. β-D-glucan assay for the diagnosis of invasive fungal infections: a meta-analysis. Clin Infect Dis. 2011; 52:750-70.
33. Hou TY, Wang SH, Liang SX, Jiang WX, Luo DD, Huang DH. The Screening Performance of Serum 1,3-Beta-D-Glucan in Patients with Invasive Fungal Diseases: A Meta-Analysis of Prospective Cohort Studies. PLoS One. 2015 Jul 6;10:e0131602.
34. Lamoth F, Cruciani M, Mengoli C, Castagnola E, Lortholary O, Richardson M, Marchetti O. β-Glucan antigenemia assay for the diagnosis of invasive fungal infections in patients with hematological malignancies: a systematic review and meta-analysis of cohort studies from the Third European Conference on Infections in Leukemia (ECIL-3). Clin Infect Dis. 2012; 54:633-43.
35. Onishi A1, Sugiyama D, Kogata Y, Saegusa J, Sugimoto T, Kawano S, Morinobu A, Nishimura K, Kumagai S. Diagnostic accuracy of serum 1,3-β-D-glucan for *Pneumocystis jirovecii* pneumonia, invasive candidiasis, and invasive aspergillosis: systematic review and meta-analysis. J Clin Microbiol. 2012; 50:7-15.
36. Karageorgopoulos DE, Qu JM, Korbila IP, Zhu YG, Vasileiou VA, Falagas ME. Accuracy of β-D-glucan for the diagnosis of *Pneumocystis jirovecii* pneumonia: a meta-analysis. Clin Microbiol Infect. 2013; 19:39-49.
37. He S1, Hang JP2, Zhang L2, Wang F2, Zhang DC3, Gong FH4 A systematic review and meta-analysis of diagnostic accuracy of serum 1,3-β-d-glucan for invasive fungal infection: Focus on cutoff levels. J Microbiol Immunol Infect; 2015 Aug;48:351-61.
38. Wong J, Zhang Y, Patidar A, Vilar E, Finkelman M, Farrington K. Is Endotoxemia in Stable Hemodialysis Patients an Artefact? Limitations of the *Limulus* Amebocyte Lysate Assay and Role of (1→3)-β-D Glucan. PLoS One. 2016 Oct 20;11(10):e0164978. doi: 10.1371/journal.pone.0164978. eCollection 2016.

Additional reference can be found on our website at Fungitell.com.

FUNGITELL® ASSAY TEST PROCEDURE QUICK GUIDE OUTLINE

SET-UP

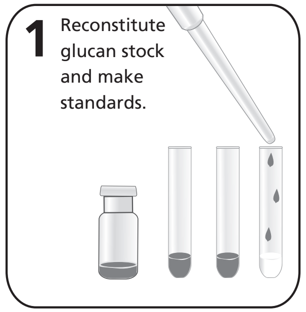

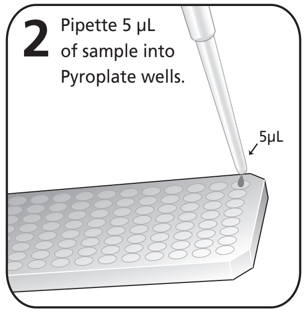

TEST SAMPLES

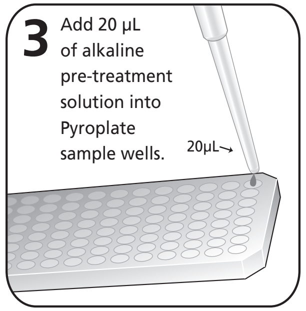

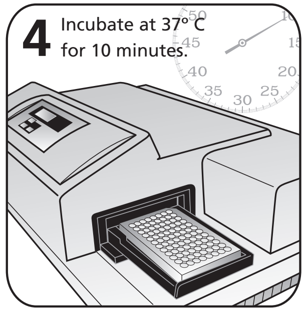

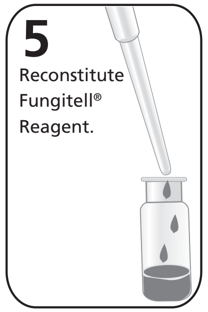

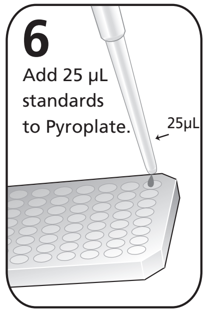

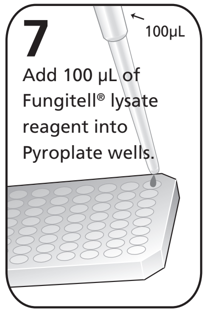

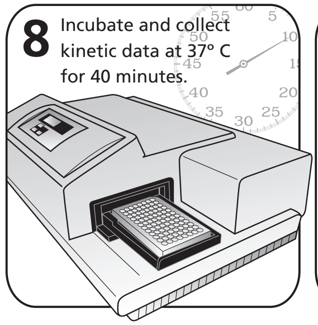

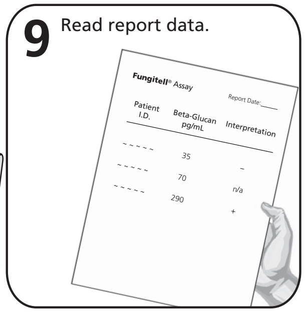

Supplement: Supplementary file 2 — Additional file 2. BG laboratory specification manual. [file 40635_2020_362_MOESM2_ESM.pdf]
