# Supplementary material for: Endotoxemia and circulating bacteriome in severe COVID-19 patients
Source: Intensive Care Med Exp. 2020 Dec 7;8:72. doi: 10.1186/s40635-020-00362-8 (PMC7719737; doi:10.1186/s40635-020-00362-8)
Supplement: Supplementary file 3 — Additional file 3: Table S1. Clinical characteristics of COVID-19 pneumonia. Table S2. Cytokines level on day 1, day 3, and day 7 stratified by ICU admission status. [file 40635_2020_362_MOESM3_ESM.docx]

**Supplementary materials**

**Supplemental Tables**

**Table S1.** Clinical characteristics of COVID-19 pneumonia

**Table S2.** Cytokines level on day 1, day 3, and day 7 stratified by ICU admission status

**Supplemental Figures**

**Figure S1.** Rarefaction curve of pass-filter reads obtained from each sample.

**Figure S2.** Dynamic bacterial community profiles on day 1, day 3, and day 7

**Figure S3.** Dynamic bacterial community profiles at the phylum level in COVID-19 patients.

**Figure S4.** Shannon diversity index (A) and Chao1 richness (B) representing alpha diversity of bacterial community profiles in COVID-19 patients on day 1, 3, and 7. The Shannon diversity index was not significant differences among the date of onsets whereas the Chao1 richness was significant (P<0.05) between the first and third date of onsets

**Figure S5.** LEfSe analysis of bacteria on day 1 and day 3

**Figure S6.** Wilcoxon matched pairs test differential abundant of bacterial genera in each COVID-19 patient between day 1 and day 3. The bacterial genera including *Sphingomonas* and *Sediminibacterium* were significantly (P<0.05) higher in the third date compared to the first of onset whereas *Comamonas, Acinetobacter and Pseudomonas* were significantly(P<0.05) decreased during the third date of onsets.

**Figure S7.** Comparison of EAA level (Figure S7a) and BG (Figure S7b) between COVID-19 and other type of infections using data from our previous studies in 136 severe sepsis or septic shock patients.

.

**Table S1.** Clinical characteristics of COVID-19 pneumonia patients

|  | 1 | 2 | 3 | 4 | 5 | 6 | 7 | 8 | 9 | 10 | 11 | 12 | 13 | 14 | 15 | 16 | 17 | 18 | 19 |
| --- | --- | --- | --- | --- | --- | --- | --- | --- | --- | --- | --- | --- | --- | --- | --- | --- | --- | --- | --- |
| Age | 50 | 76 | 59 | 72 | 48 | 41 | 54 | 59 | 43 | 38 | 50 | 38 | 57 | 57 | 24 | 48 | 58 | 64 | 58 |
| Sex | M | M | M | M | M | F | M | M | M | M | M | M | M | F | F | M | M | M | F |
| U/D | DMT2 | none | DMT2 | DMT2, HT, DLP | none | HBV cirrhosis | none | HT, gout | DMT2 | none | DMT2, HT, DLP | none | DCM | none | none | none | KT,DMT2, HT, DLP | MM, DMT2, HT, DLP | HIV infection |
| Fever day before enrollment | 9 | 14 | 9 | 9 | 9 | 0 | 9 | 15 | 12 | 3 | 9 | 8 | 11 | 9 | 11 | 13 | 11 | 3 | 11 |
| ICU admission | Y | Y | Y | Y | Y | Y | N | N | N | N | Y | Y | Y | Y | N | N | Y | Y | Y |
| APACHE II score | 17 | 9 | 10 | 7 | 3 | 5 | none | none | none | none | 8 | 4 | 4 | 4 | none | none | 12 | 22 | 11 |
| PaO2/FiO2 ratio at enrollment | 186 | 337 | 246 | 246 | 117 | 293 | no data | no data | no data | no data | 184 | 233 | 190 | 261 | no data | no data | 212.5 | 145 | 281 |
| EAA at day 1 | 0.86 | 0.89 | 0.81 | 0.14 | 0.86 | 0.78 | 0.42 | 0.91 | 0.25 | 0.68 | 0.45 | 0.57 | 0.47 | 0.57 | 2.32 | 0.57 | 0.54 | 0.58 | 0.48 |
| Peak EAA | 0.95 | 0.96 | 2.79 | 0.46 | 0.86 | 2.76 | 0.42 | 0.91 | 1.33 | 0.68 | 0.47 | 0.57 | 0.7 | 1.0 | 2.32 | 0.62 | 0.84 | 0.58 | 2.58 |
| IL-6 (pg/mL) at day 1 | 3237 | 8437 | 495.9 | 13.79 | 100.7 | 12.61 | 11.96 | 39.46 | 7.61 | 3.18 | 2887 | 41.84 | 65.58 | 16.85 | 20.65 | 65.69 | 15.53 | 437.8 | 868.5 |
| Peak IL-6 (pg/mL) | 3237 | 8437 | 549.1 | 13.79 | 100.7 | 37.53 | 11.96 | 39.46 | 7.97 | 3.18 | 2887 | 164.4 | 65.58 | 16.85 | 20.65 | 65.69 | 455.5 | 620 | 1024 |
| BG (pg/mL) at day 1 | 13.4 | 33.6 | <7.8 | 9.9 | 9.6 | 8.67 | 78 | <7.8 | <7.8 | 28.4 | <7.8 | 41.1 | 106 | 14.3 | 40.9 | 25.3 | 134.1 | 72.1 | 14.5 |
| Peak BG (pg/mL) | >523.4 | 33.6 | 65 | 126 | 448 | 139 | 78 | <7.8 | >523 | 28.4 | 233 | 41.1 | >523.4 | 23.1 | 40.9 | 26.6 | >523.4 | 72.1 | 24.9 |
| Treatment |  |  |  |  |  |  |  |  |  |  |  |  |  |  |  |  |  |  |  |
| Darunavir+Ritonavir | Y | Y | Y | Y | Y | N | N | Y | N | N | N | N | Y | N | Y | N | N | N | Y |
| Antimalarial drug | Y | Y | Y | Y | Y | Y | Y | Y | Y | Y | Y | Y | Y | Y | Y | Y | N | Y | Y |
| Favipiravir | Y | Y | Y | Y | Y | Y | Y | N | Y | Y | Y | Y | Y | Y | Y | Y | Y | Y | Y |
| Azithromycin | Y | Y | Y | N | Y | Y | Y | N | Y | Y | Y | Y | N | Y | N | Y | N | Y | N |
| Corticosteroid | Y | Y | Y | N | N | N | N | N | N | N | N | N | N | N | N | N | Y | N | N |
| Antibiotics | Y | Y | Y | Y | Y | Y | N | N | N | N | Y | Y | Y | Y | Y | N | Y | Y | Y |
| HP therapy | oXiris | N | PMX | N | HA330 | HA330 | N | N | N | N | N | HA330 | PMX | N | N | N | PMX | PMX | PMX |
| Outcome at 28 day |  |  |  |  |  |  |  |  |  |  |  |  |  |  |  |  |  |  |  |
| Dead | N | N | N | N | N | N | N | N | N | N | N | N | N | N | N | N | N | N | N |
| ICU admission day | 28 | 12 | 28 | 1 | 13 | 6 | 0 | 0 | 0 | 0 | 28 | 5 | 14 | 3 | 0 | 0 | 2 | 28 | 10 |
| Mechanical ventilator day | 28 | 9 | 25 | 0 | 5 | 4 | 0 | 0 | 0 | 0 | 7 | 4 | 9 | 0 | 0 | 0 | 0 | 28 | 6 |
| Prone position | Y | N | Y | N | N | N | N | N | N | N | N | N | N | N | N | N | N | N | N |
| ECMO | Y | N | N | N | N | N | N | N | N | N | N | N | N | N | N | N | N | N | N |
| Bacterial infection |  |  |  |  |  |  |  |  |  |  |  |  |  |  |  |  |  |  |  |
| Site | Pneumonia | Pneumonia | Pneumonia | N | Pneumonia | N | N | N | N | N | Pneumonia | Pneumonia | N | Pneumonia | N | N | N | Pneumonia | Septicemia |
| Organisms | Acinetobacter baumannii | Acinetobacter baumannii | Acinetobacter baumannii | N | Klebsiella pneumoniae | N | N | N | N | N | Klebsiella pneumoniae | Pseudomonas aeruginosa | N | Klebsiella pneumoniae | N | N | N | Klebsiella pneumoniae | Acinetobacter baumannii |
| AKI | Y | N | Y | Y | N | N | N | N | N | N | N | Y | Y | N | N | N | Y | Y | Y |
| RRT | Y, renal recovery | N | Y, no renal recovery | N | N | N | N | N | N | N | N | N | N | N | N | N | N | Y, renal recovery | N |

AKI, acute kidney injury; APACHE II score, Acute Physiology and Chronic Health Evaluation II score; BG, (1→3)-β-D-Glucan; DCM, dilated cardiomyopathy; DLP, dyslipidemia; DMT2, diabetes mellitus type 2; EAA, endotoxin activity assay; ECMO, extracorporeal membrane oxygenation; F, female; FiO2, fractional inspired oxygen, HBV, hepatitis B virus; HIV, Human Immunodeficiency Virus; HP, hemoperfusion; HT, hypertension; ICU, intensive care unit; IL-6, Interleukin 6; KT, kidney transplantation; M, male; MM, multiple myeloma; N, no; RRT, renal replacement therapy; PaO2, partial pressure of arterial oxygen; PMX, Polymyxin B hemoperfusion; U/D, underlying disease; Y, yes.

**Table S2** Cytokines on day 1, day 3, and day 7 by ICU admission

| **Cytokines** | **Total (N=19)** | **Patients not in ICU^+^** | **Patients in ICU^++^** | **P value** |
| --- | --- | --- | --- | --- |
| IL-1β |  |  |  |  |
| Day 1 | 1.7 (1.7, 2.8) | 1.7 (1.7, 8.2) | 1.7 (1.7, 1.7) | 0.43 |
| Day 3 | 1.7 (1.7, 2.3) | 3.2 (1.7, 6.4) | 1.7 (1.7, 1.7) | 0.21 |
| Day 7 | 1.7 (1.7, 1.7) | 1.7 (1.7, 1.7) | 1.7 (1.7, 1.7) | 0.60 |
| IFN- α2 |  |  |  |  |
| Day 1 | 2.7 (1.9, 6.9) | 2.8 (1.9, 6.6) | 2.7 (1.9, 6.9) | 0.93 |
| Day 3 | 1.9 (1.9, 2.8) | 1.9 (1.9, 7.0) | 1.9 (1.9, 2.8) | 0.75 |
| Day 7 | 1.9 (1.9, 2.1) | 7.8 (7.8, 7.8) | 1.9 (1.9, 1.9) | 0.043 |
| IFN-y |  |  |  |  |
| Day 1 | 10 (10, 10) | 10 (10, 10) | 10 (10, 10) | 0.14 |
| Day 3 | 10 (10, 10) | 10 (10, 2529) | 10 (10, 10) | 0.071 |
| Day 7 | 10 (10, 10) | 4061 (4061, 4061) | 10 (10, 10) | <0.001 |
| TNF-a |  |  |  |  |
| Day 1 | 4.1 (4.1, 4.1) | 4.1 (4.1, 4.1) | 4.1 (4.1, 4.1) | 0.32 |
| Day 3 | 4.1 (4.1, 4.1) | 4.1 (4.1, 4.1) | 4.1 (4.1, 4.1) | 0.58 |
| Day 7 | 4.1 (4.1, 4.1) | 4.1 (4.1, 4.1) | 4.1 (4.1, 4.1) | NA |
| MCP-1 |  |  |  |  |
| Day 1 | 410 (286, 1053) | 285 (215, 300) | 848 (410, 1782) | 0.003 |
| Day 3 | 599 (333, 830) | 289 (244, 424) | 755 (410, 867) | 0.070 |
| Day 7 | 491 (261, 762) | 261 (261, 261) | 535 (261, 762) | 0.46 |
| IL-6 |  |  |  |  |
| Day 1 | 25 (11, 233) | 12 (7, 25) | 45 (16, 334) | 0.023 |
| Day 3 | 25 (12, 155) | 6 (4, 11) | 86 (14, 175) | 0.017 |
| Day 7 | 27 (10, 52) | 8 (8, 8) | 33 (13, 52) | 0.26 |
| IL-8 |  |  |  |  |
| Day 1 | 64 (34, 126) | 24 (20, 41) | 73 (47, 128) | 0.009 |
| Day 3 | 67 (33, 109) | 33 (29, 37) | 99 (57, 125) | 0.031 |
| Day 7 | 46 (29, 99) | 29 (29, 29) | 49 (32, 99) | 0.38 |
| IL-10 |  |  |  |  |
| Day 1 | 19 (9, 35) | 8 (2, 13) | 23 (19, 56) | 0.022 |
| Day 3 | 15 (6, 25) | 5 (3, 11) | 21 (11, 27) | 0.089 |
| Day 7 | 4 (2, 17) | 10 (10, 10) | 4 (2, 17) | 0.71 |
| IL-12p70 |  |  |  |  |
| Day 1 | 2.0 (1.4, 3.3) | 2.5 (1.4, 3.3) | 1.9 (1.4, 2.8) | 0.55 |
| Day 3 | 1.4 (1.4, 3.0) | 2.8 (2.0, 3.0) | 1.4 (1.4, 2.6) | 0.39 |
| Day 7 | 1.4 (1.4, 4.1) | 1.4 (1.4, 1.4) | 1.4 (1.4, 4.1) | 0.41 |
| IL-17A |  |  |  |  |
| Day 1 | 0.8 (0.8, 3.0) | 1.6 (0.8, 6.9) | 0.8 (0.8, 2.9) | 0.45 |
| Day 3 | 0.8 (0.8, 3.5) | 0.8 (0.8, 2.2) | 0.8 (0.8, 3.5) | 0.45 |
| Day 7 | 0.8 (0.8, 1.3) | 0.8 (0.8, 0.8) | 0.8 (0.8, 1.3) | 0.53 |
| IL-18 |  |  |  |  |
| Day 1 | 114 (90, 190) | 116 (102, 129) | 105 (85, 207) | 0.93 |
| Day 3 | 129 (72, 223) | 96 (61, 146) | 137 (106, 266) | 0.31 |
| Day 7 | 120 (55, 205) | 138 (138, 138) | 103 (55, 205) | 0.90 |
| IL-23 |  |  |  |  |
| Day 1 | 1.9 (1.2, 3.2) | 2.0 (1.2, 3.2) | 1.9 (1.4, 3.2) | 0.59 |
| Day 3 | 1.8 (1.3, 2.7) | 1.9 (1.4, 2.6) | 1.8 (1.2, 2.7) | 0.73 |
| Day 7 | 1.3 (1.2, 3.9) | 1.3 (1.3, 1.3) | 1.3 (1.2, 3.9) | 0.90 |
| IL-33 |  |  |  |  |
| Day 1 | 6.4 (2.9, 13.4) | 4.7 (2.5, 9.3) | 6.7 (5.4, 16.3) | 0.24 |
| Day 3 | 5.7 (3.9, 8.3) | 3.9 (3.5, 6.1) | 6.4 (5.2, 9.1) | 0.21 |
| Day 7 | 5.6 (3.3, 16.5) | 5.7 (5.7, 5.7) | 5.5 (3.3, 16.5) | 0.90 |

* Data are presented as median (IQR).

^+^ N=6 (Day 1), N = 4 (Day 3), N =1 (Day 7)

^++^ N=13 (Day 1), N= 13 (Day 3), N=13 (Day 7)
