# Supplementary material for: Endotoxemia and circulating bacteriome in severe COVID-19 patients
Source: Intensive Care Med Exp. 2020 Dec 7;8:72. doi: 10.1186/s40635-020-00362-8 (PMC7719737; doi:10.1186/s40635-020-00362-8)
Supplement: Supplementary file 4 — Additional file 4: Figure S1. Rarefaction curve of pass-filter reads obtained from each sample. Figure S2. Dynamic bacterial community profiles on day 1, day 3, and day 7. Figure S3. Dynamic bacterial community profiles at the phylum level in COVID-19 patients. Figure S4. Shannon diversity index (A) and Chao1 richness (B) representing alpha diversity of bacterial community profiles in COVID-19 patients on day 1, 3, and 7. The Shannon diversity index was not significant differences among the date of onsets whereas the Chao1 richness was significant (P < 0.05) between the first and third date of onsets Figure S5. LEfSe analysis of bacteria on day 1 and day 3. Figure S6. Wilcoxon matched pairs test differential abundant of bacterial genera in each COVID-19 patient between day 1 and day 3. The bacterial genera including Sphingomonas and Sediminibacterium were significantly (P < 0.05) higher in the third date compared to the first of onset whereas Comamonas, Acinetobacter and Pseudomonas were significantly (P < 0.05) decreased during the third date of onsets. Figure S7. Comparison of EAA level (Figure S7a) and BG (Figure S7b) between COVID-19 and other type of infections using data from our previous studies in 136 severe sepsis or septic shock patients. [file 40635_2020_362_MOESM4_ESM.pptx]

## Slide 1
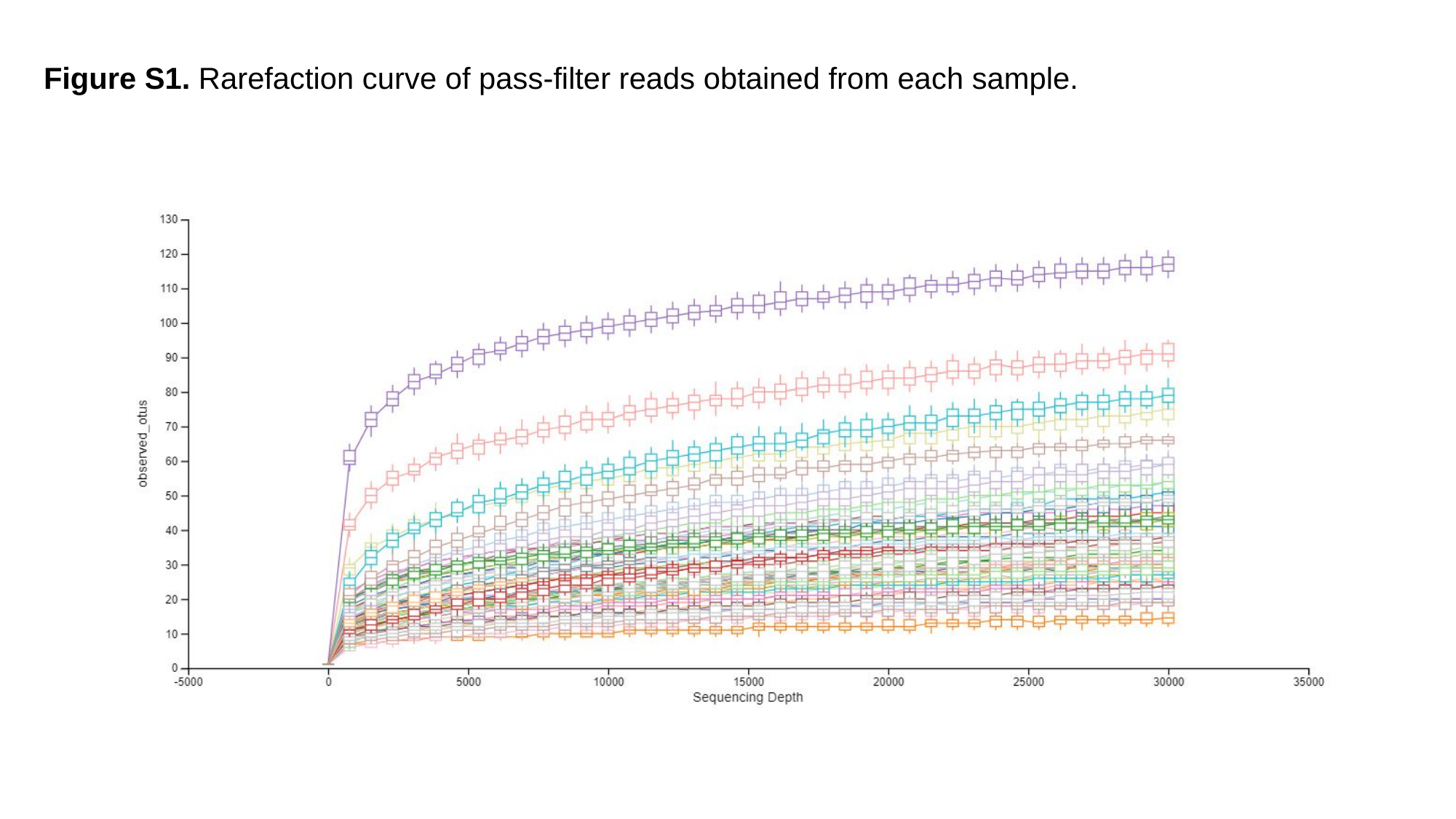

Figure S1. Rarefaction curve of pass-filter reads obtained from each sample.

## Slide 2
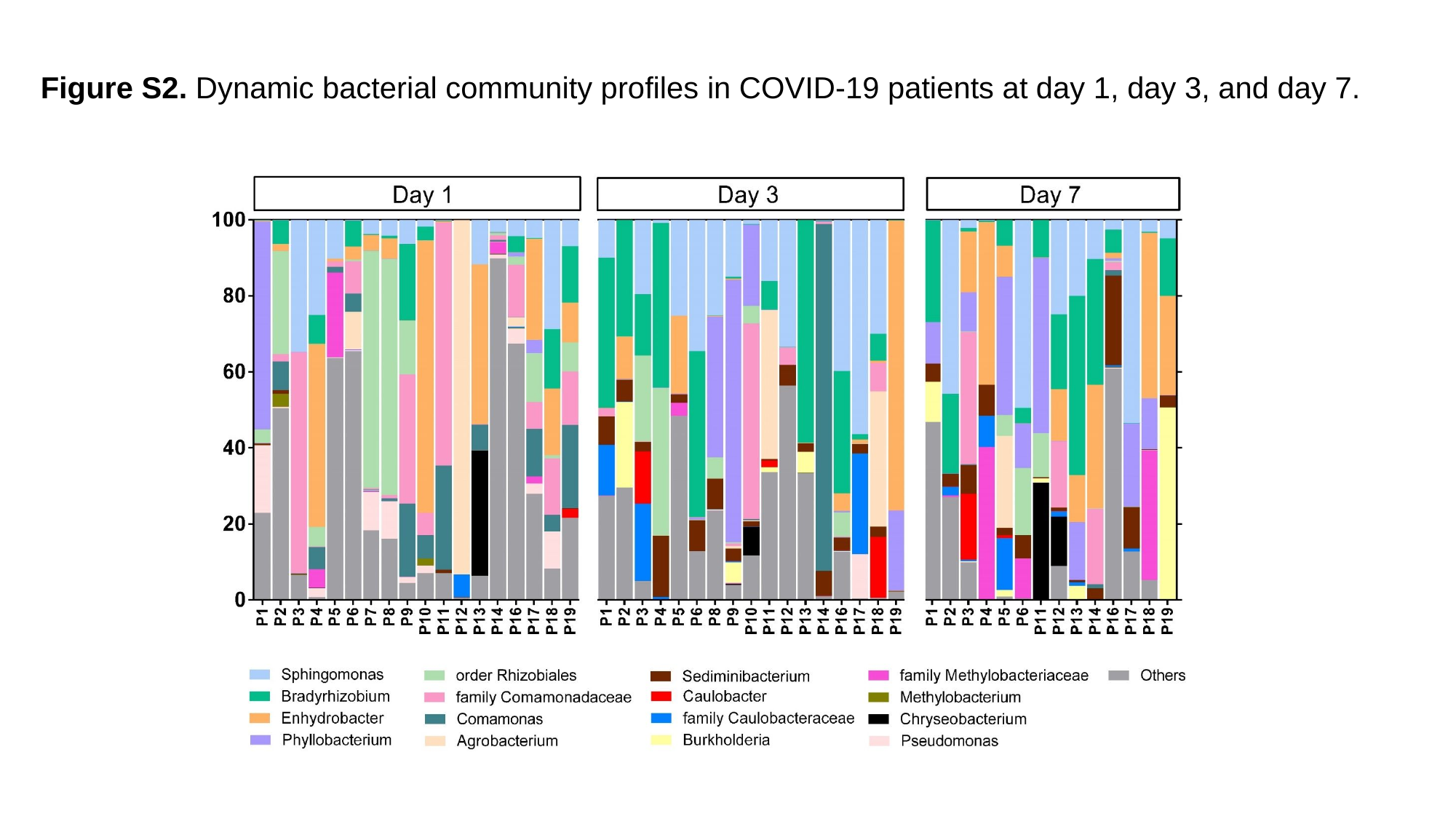

Figure S2. Dynamic bacterial community profiles in COVID-19 patients at day 1, day 3, and day 7.

## Slide 3
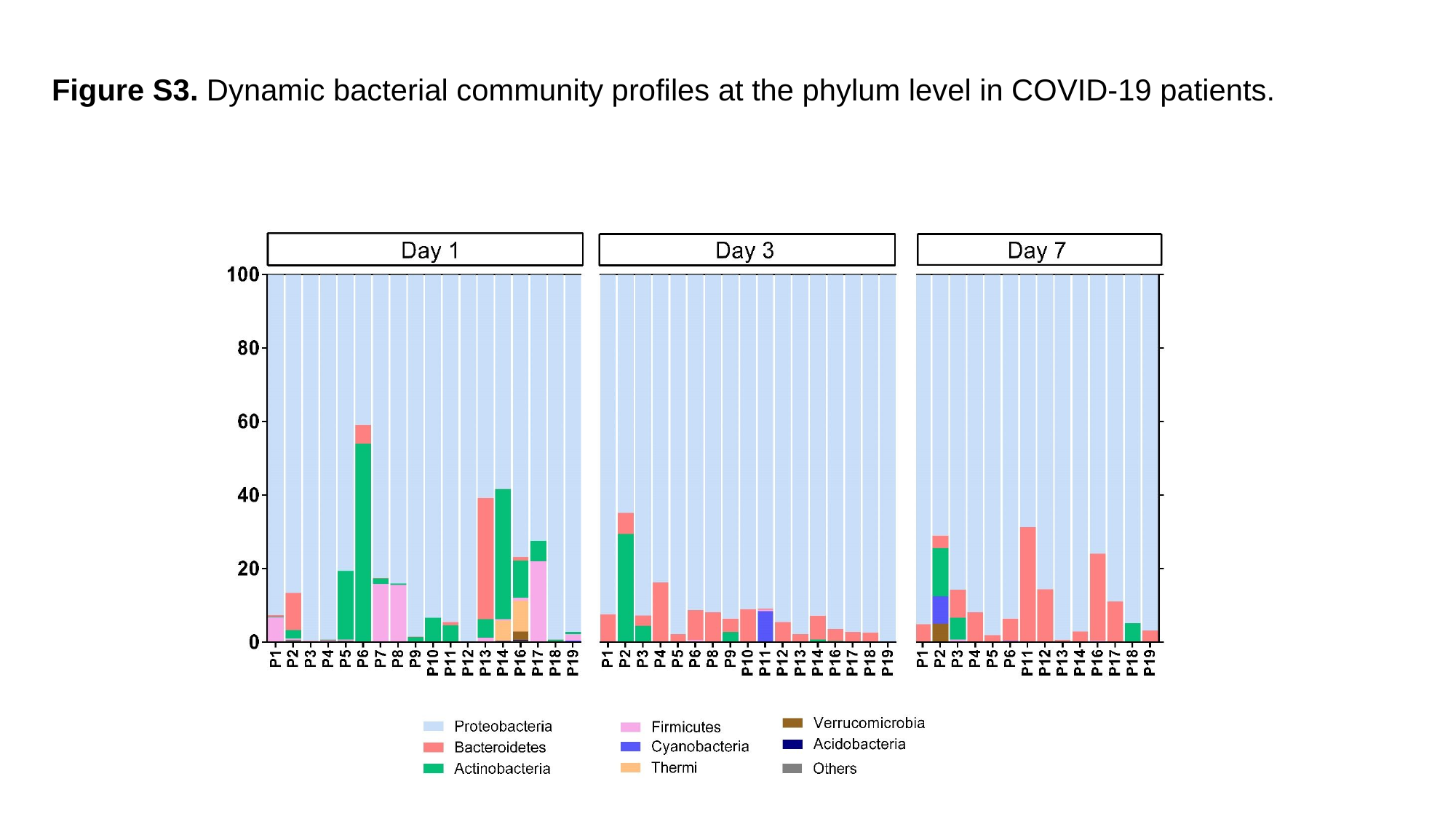

Figure S3. Dynamic bacterial community profiles at the phylum level in COVID-19 patients.

## Slide 4
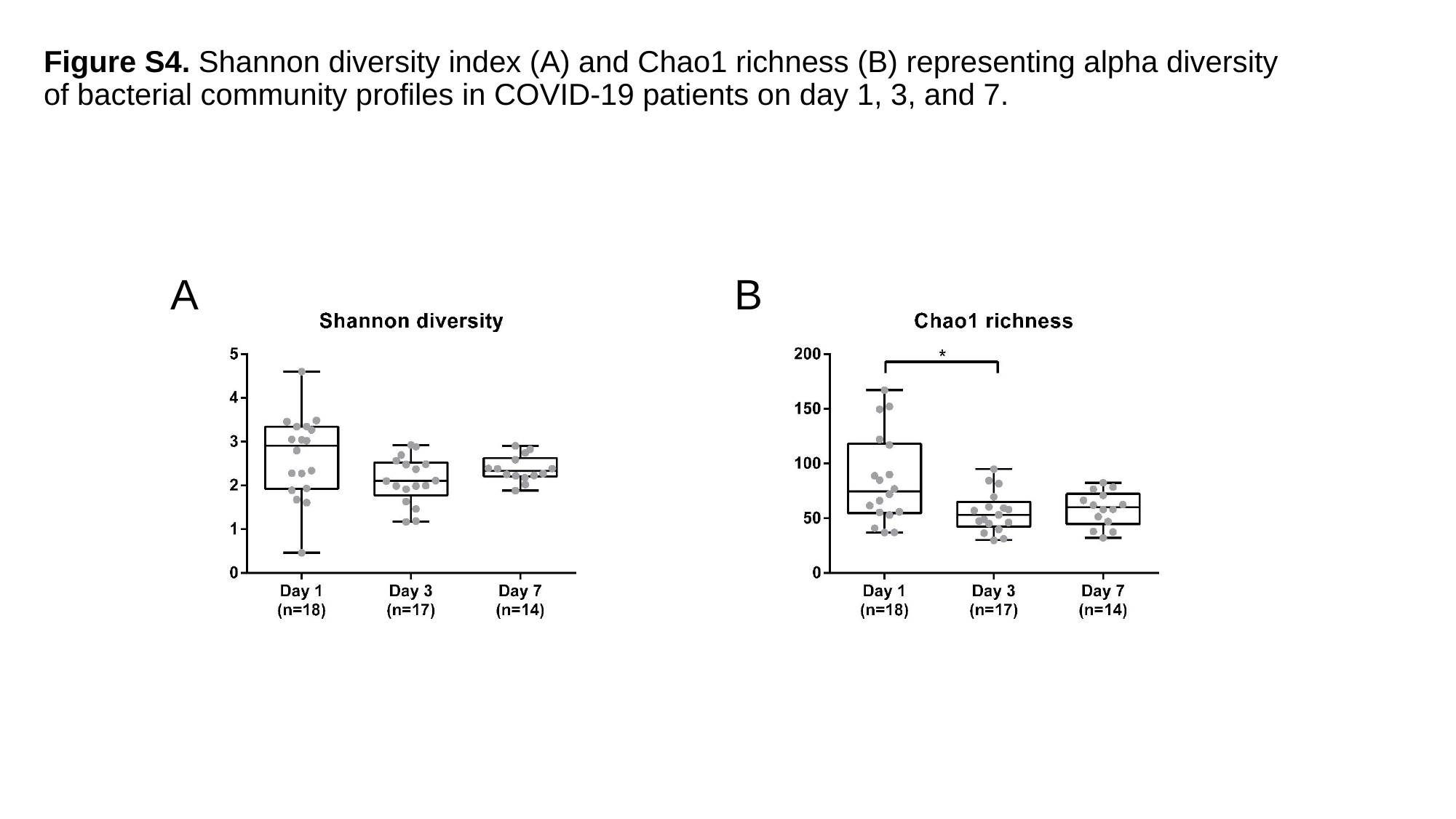

Figure S4. Shannon diversity index (A) and Chao1 richness (B) representing alpha diversity of bacterial community profiles in COVID-19 patients on day 1, 3, and 7.
A
B

## Slide 5
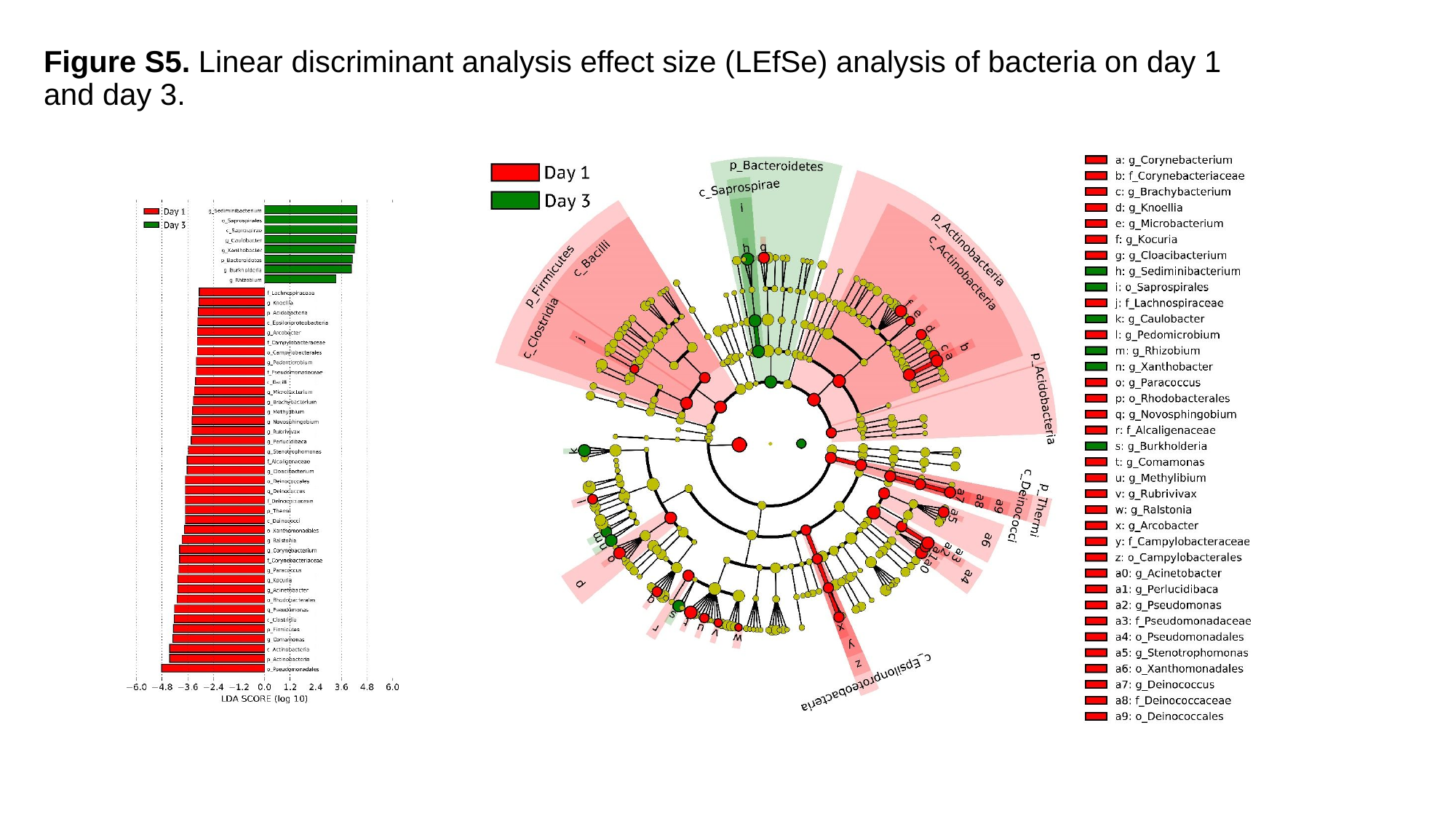

Figure S5. Linear discriminant analysis effect size (LEfSe) analysis of bacteria on day 1 and day 3.

## Slide 6
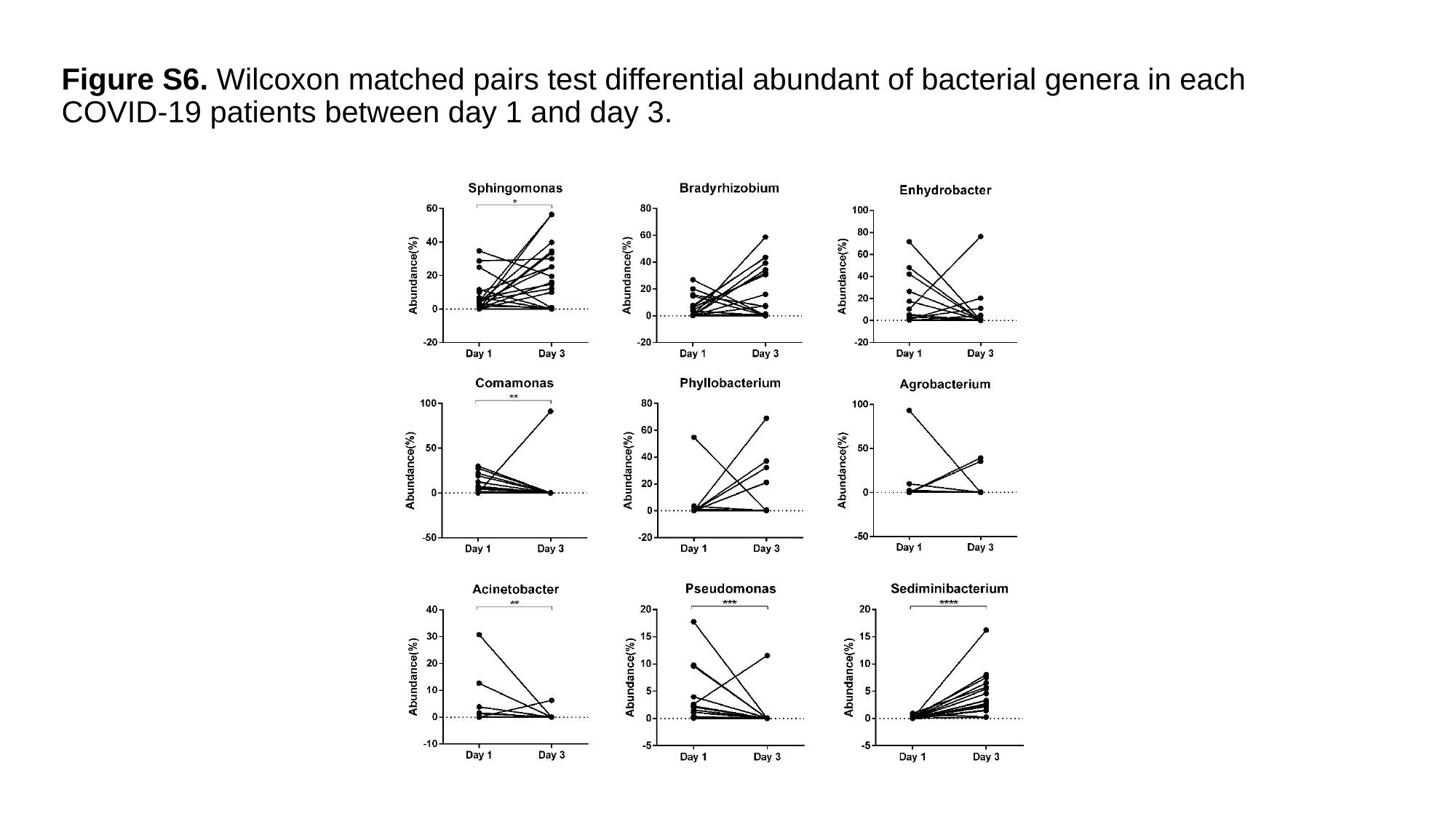

Figure S6. Wilcoxon matched pairs test differential abundant of bacterial genera in each
COVID-19 patients between day 1 and day 3.

## Slide 7
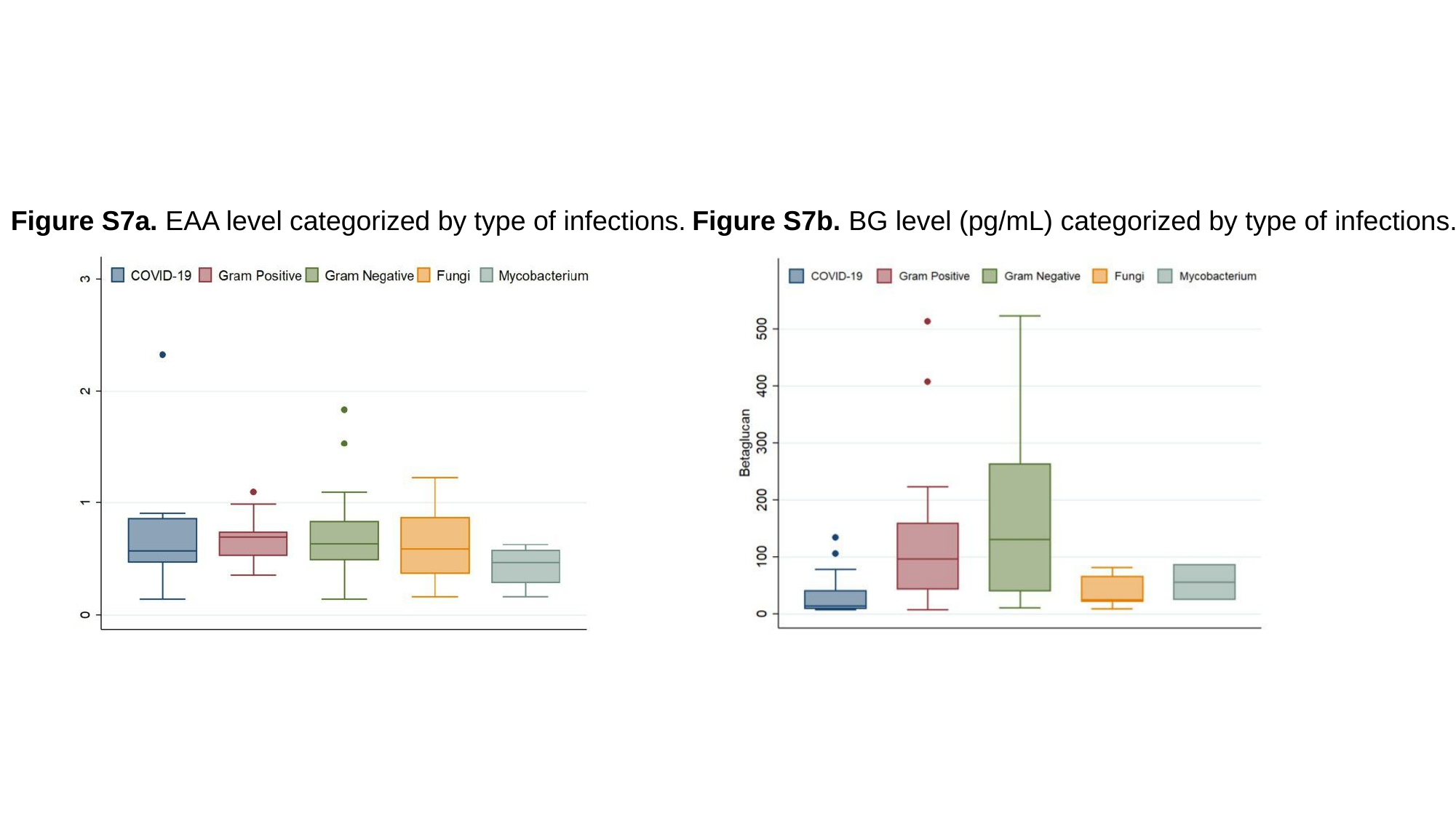

Figure S7a. EAA level categorized by type of infections.
Figure S7b. BG level (pg/mL) categorized by type of infections.
